# Managing free-roaming domestic dog populations using surgical sterilisation: a randomised controlled trial

**DOI:** 10.1038/s41598-025-98990-1

**Published:** 2025-04-24

**Authors:** H. R. Fielding, K. A. Fernandes, Amulya V.R., D. Belgayer, A. Misquita, R. Kenny, A. D. Gibson, L. Gamble, B. M. C. Bronsvoort, R. J. Mellanby, I. Handel, D. Rivett, K. Newman, R. King, I. Sayyed, A. Sayed, K. Lad, M. Yaraguda, A. D. Parate, M. K. Balagali, S. Mazeri

**Affiliations:** 1 The Roslin Institute and the Royal (Dick) School of Veterinary Studies (R(D)SVS), Easter Bush, Midlothian, EH25 9RG UK; 2 Worldwide Veterinary Service, Ooty, Tamil Nadu India; 3 Department of Animal Husbandry and Veterinary Services, Government of Goa and the Goa Veterinary Association, Pashusamwardhan Bhavan, Patto, Panaji, Goa 403401 India; 4 Worldwide Veterinary Service, 4 Castle Street, Cranborne, Dorset, BH21 5PZ UK; 5 School of Mathematics and Maxwell Institute for Mathematical Sciences, University of Edinburgh, Edinburgh, UK; 6 Biomathematics & Statistics Scotland, James Clerk Maxwell Building, Peter Guthrie Tait Road, The King’s Buildings, Edinburgh, EH9 3FD UK

**Keywords:** Canine, Fertility control, Free-ranging dog, Neuter, Population management, Stray, Population dynamics, Urban ecology

## Abstract

Free-roaming domestic dogs (FRDs) are among the most abundant carnivores on earth and have coexisted with humans for over 15,000 years, yet increases in negative interactions and the transmission of zoonotic diseases, precipitates calls for population management. Despite significant investment in FRD sterilisation in India, where rabies is endemic, there is limited evidence of its impact on reducing FRD population sizes. Therefore, robust evaluation of the effectiveness of fertility control programmes is necessary. To address this, we implemented a Before After Control Intervention (BACI) framework in the first multi-site randomised controlled trial for the sterilisation of FRDs. We conducted single intensive sterilisation campaigns in five areas, achieving female sterilisation coverages of 58–66%. We observed a decrease in puppies and lactating females and a reduction in residents’ reports of barking, a common problem associated with FRDs. There were no significant differences in adult FRD counts between intervention and control sites during the 2-year follow-up. However, unmeasured immigration into and emigration out of study areas may have confounded counts. One-off, albeit intense, sterilisation campaigns in open populations require substantial investment and are unlikely to reduce population size in isolation, though there may be some reduction in problematic behaviours and improved animal welfare.

**Supplementary Information:**

The online version contains supplementary material available at 10.1038/s41598-025-98990-1.

## Introduction

Human population expansion, climate change, and structural changes to the environment can force non-human animals to shift their habitats and behaviours, leading to increased contact with humans^1^. This increases the risks of pathogen transmission, disturbance of ecological processes, and public health concerns^2^. Conflicts arising from these interactions often prompt human attempts to manage animal populations^3^. Despite living successfully with humans for around 15,000 years with many mutual benefits, conflicts between domestic dogs (*Canis familiaris*) and humans persist and management of free-roaming dogs (FRD) is common^4^. Domestic dogs pose public health issues through injuries^5^ and zoonotic pathogens^6^ including rabies, which kills ~ 60,000 people annually, mostly in low to middle-income countries^7,8^. Dog bites and rabies primarily affect people of lower-economic status, and death rates are high in rural areas, where people are less able to access treatment and rabies prevention measures^5^. Fear of dog attacks can restrict people’s economic and lifestyle choices and FRDs can negatively impact local economies through reduced tourism^9^. Environmental concerns include nitrate pollution from canine excrement and noise pollution from barking, one of the most common problems associated with FRDs^9,10^. Predation, disease transmission, and resource competition further exacerbate conflicts in areas where wildlife and dog habitats overlap^11,12^.

There are multiple approaches to dog population management^13^. Lethal control methods, i.e. culling, can quickly reduce population size, yet often result in younger, naïve populations that are more mobile and more susceptible to disease, as demonstrated in badger populations^14^. Moreover, culling is ineffective in the control of rabies in many settings^15,16^ and the World Organisation for Animal Health (WOAH) does not consider it to be an effective or sustainable way to manage FRD populations^17^. Culling is also considered culturally unacceptable in many regions^18^. An alternative and more widely accepted management practice is a catch-neuter release (CNR) programme- where FRDs are captured, sterilised, then returned to the capture location. While more humane, CNR is costly, labour intensive, and lacks consistent empirical evidence of efficacy. For instance, sustained sterilisation in Jaipur, India, over 5 years led to a 28% reduction in dog numbers^19^. Yet, in six areas of Jodhpur, India, repeated sterilisation over 2 years resulted in reductions ranging from 3 to 51%^20^. Studies in Bangkok observed a 25% decrease in dog counts over 5 years with a mean sterilisation rate of 0.48 FRD per km of surveyed roads^21^. Conversely, FRD populations in Ukraine increased over 15-months in regions with 17–52% sterilisation coverage, but the authors acknowledged that a longer data collection and control regions were needed to fully interpret this observation^22^.

None of these studies recorded dog population trends in comparable control areas, leaving open the possibility that changes were due to external factors or natural fluctuations. In the only controlled intervention study known to the authors, over 80% sterilisation coverage in one site in Brazil showed no changes in abundance, survival, or recruitment compared to the control site over 14 months in two groups of less than 100 dogs^23^.

Theoretical studies have attempted to predict population changes post-sterilisation^20,24–28^, but accurately simulating real-world mechanisms such as movement between sub-populations and changes in survival and fecundity remains challenging due to limited empirical data^29,30^. These factors likely influence model predictions significantly^27^. Several reviews have concluded that data collection in multiple sites before and after the intervention and a non-intervention control site (i.e. BACI designs^31^) is necessary to accurately assess the impacts of sterilisation on FRD populations and provide more data for modelling scenarios^13,32,33^.

**Fig. 1:**
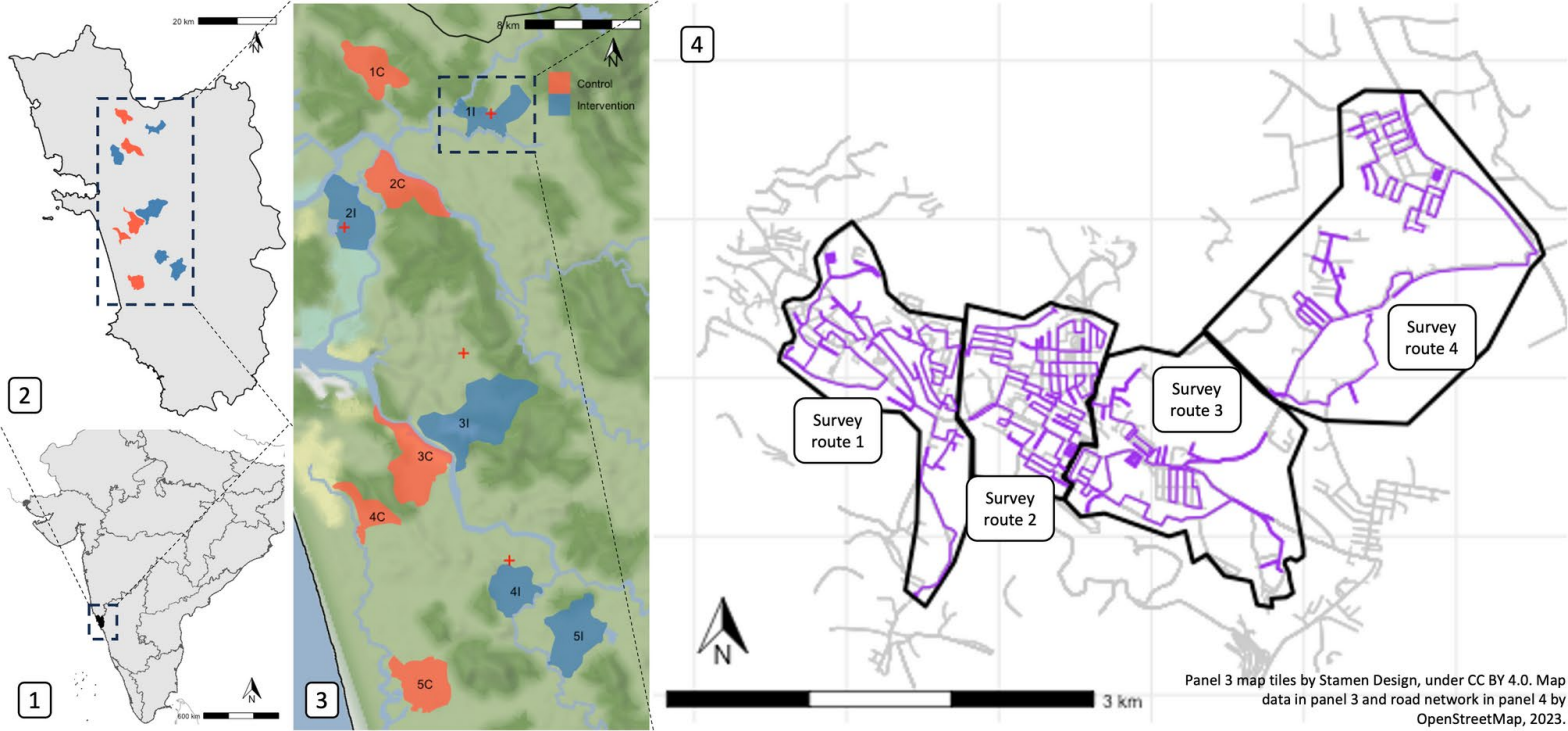
Study site location: Panel 1 shows partial map of India with Goa in black; panel 2 shows Goa state with intervention (blue) and control sites (red). Panel 3 shows study sites in context of elevation and waterways and panel 4 shows intervention site 1I with road network from OpenStreetMap under the Open Database License (ODbL; grey lines) and free-roaming dog survey routes (purple lines) within survey zones (black outline). State and country boundaries shown were obtained from rnaturalearth. Map tiles by Stamen Design, under CC BY 4.0. Data by OpenStreetMap, available under ODbL.

To address this need, we conducted the first randomised-controlled multi-site trial of sterilisation in FRDs. Our intense capture-sterilise-return program was implemented at five sites randomly selected from 10 paired sites in Goa, India (Fig. 1; map data from OpenStreetMap under the Open Database License). Using strict survey protocols, FRD counts and dog information (Table S1) were recorded by trained surveyors in all sites 7 months prior to and 26 months after the intervention (Fig. S1). We also recorded human perceptions of FRDs pre- and post-intervention to assess any change in the perceived ‘problem’ of FRDs. Sterilisation is considered by some as a panacea for FRD aggression towards humans, livestock, wildlife, other FRDs and pet dogs. However, the relationship between sterilisation and behaviour is complex, likely to vary between sexes and individuals, with one of the only randomized controlled trials showing no reduction of aggression in male FRDs after sterilisation^34^. Therefore, we asked the local community if they noticed changes in barking and aggression towards FRDs, humans, and pet dogs (livestock and wildlife were not common in our study areas therefore omitted). This paper describes the impacts of the sterilisation intervention on FRD population size and structure, dog health and community perceptions and discusses implications for dog population management programmes.

## Methods

The study had a randomised block design with repeated counts over time. There were five blocks and two experimental units per block (study sites) with one unit randomly selected (randomly sampled each pair of site IDs in R^35^) to receive the intervention (surgical sterilisation of dogs) and the other the control (no action; Fig. 1). Dog count surveys were carried out along specified routes during fourteen primary sampling periods starting in May 2020. Sterilisations in intervention sites started on a staggered basis in November 2020 after three primary sampling periods and were followed by at least 9 primary sampling periods until January 2023 (Fig. S1). Based on dog counts before the intervention, power calculations demonstrated that five blocks would provide > 80% power to detect a change in the dog population of at least 10% (using the power. BACI function in the R package, emon^36^). Ten sites were outlined based on local administrative boundaries in Goa state, India. Goa is a relatively small and affluent state on India’s south-west coast with a monsoon climate, relying heavily on tourism and fishing industries. It has a substantial FRD population that has been monitored via rabies vaccination programmes since 2013^37^ and high-quality veterinary infrastructure to facilitate large scale FRD sterilisation, therefore was an appropriate site for our study. Sites within blocks were based on similar human population ^38^, geographical size and location (Table 1). Sites had either a 2 km buffer (which encompassed the reported average home range of Indian FRDs at < 0.5 km^2^ and mean dispersal distance of 1.7 km^2^^39,40^) or major river separating them (Fig. 1) to reduce the risk of dog movements between control and intervention areas.

**Table 1:** Study site information: Geographic, dog demographic and human demographic information for each study site.

Site	Site area km^2^	Estimated FRD pop May–Nov 2022 (95% CI)	FRD per km^2^	Humans per km^2^	Human:dog ratio	Pre-int. mean SC % (SD)	Post-int. highest SC % (SD)
1C	9.6	1806 (1624–1989)	188.9	1437.4	7.6	30.0 (2.9)	34.4 (4.8)
1I	8.5	1363 (1190–1535)	159.9	1277.1	8.0	26.2 (3.4)	65.8 (5.5)
2C	12.1	1268 (1106–1430)	104.7	664.0	6.3	28.2 (7.8)	32.7 (4.4)
2I	11.9	1071 (949–1194)	90.2	750.5	8.3	16.0 (5.1)	61.8 (6.6)
3C	15.7	539 (355–724)	34.4	395.1	11.5	10.7 (3.8)	28.3 (11.3)
3I	24.8	1167 (976–1357)	47.0	423.7	9.0	12.1 (5.5)	64.1 (6.2)
4C	6.2	693 (570–816)	111.2	710.1	6.4	9.0 (2.7)	23.4 (6)
4I	8.9	291 (241–341)	32.9	1948.6	59.1	18.9 (1.6)	63.9 (9.2)
5C	11.7	644 (534–753)	54.8	295.1	5.4	9.4 (1.5)	22.1 (7.7)
5I	14.1	791 (676–906)	56.2	1180.6	20.9	11.9 (1.2)	57.6 (8.3)

Site area was calculated in QGIS v3.4 and road networks were obtained from OpenStreetMap (https://www.openstreetmap.org). Adult dog population estimates are based on mark-resight surveys in May to October 2022 (Chapman method, SI 1.5) and human population estimates from WorldPop data^38^.

C, control; I, intervention; FRD, free-roaming domestic dog; Int, intervention, SC, FRD count survey female sterilisation coverage, CI, confidence interval, SD, standard deviation.

### Count surveys

Each of the fourteen primary sampling periods consisted of secondary dog count surveys repeated along the same route approximately four times in the morning (07:00) and four times in the afternoon (15:30). Each secondary survey comprised 3–5 survey routes about 10–15 km long per study site (Fig. 1). Survey routes aimed to achieve maximum coverage of roads accessible throughout the year within 2–3 h on a two-wheeler motorised vehicle (110 cc), without excessive back-tracking to minimise the chances of recording the same dog twice as dogs were not individually identified. Surveyors drove at a walking pace no faster than 20 km per hour and stopped to record dogs when sighted. Counts from survey routes within a study site were summed to obtain a site survey count for each secondary sampling point. Only FRDs were counted, defined as domestic dogs ‘observed without human supervision on public property or on private property with immediate unrestrained access to public property’^41^. The probability of seeing dogs is unknown, however, we expect it to be a function of distance from the path and visibility. Due to the heterogeneous environments on our routes (e.g. urban, forest, open space), visibility varied substantially and we were not able to use distance-sampling techniques^42^. Surveyors regularly checked for any changes to sightability on a route, e.g. new road or buildings and no changes occurred during the study. Estimated age category (puppy: 0–3 months, juvenile: > 3–11 months, adult: >  = 12 months), sex, sterilisation status (defined by ear notch, as recommended in Animal Birth Control rules of India^43^ or absence of testicles in males), lactation status, body and skin condition, and location were recorded in a purpose-built smartphone application (Table S1, SI.1.2 & SI.5.1–3). Surveyors were blinded to control and intervention site types. Seven surveyors performed surveys, three of whom remained for the duration of the study. Surveyors had regular training and were shadowed by peers and management to ensure a consistent approach was used (see SI.1.2). Surveyors used a monocular to check details of dogs, e.g. ear notch or sex, but were encouraged to select ‘unknown’ if there was doubt about these details. All surveyors surveyed in all sites and routes on a rotation system, to offset any inter-observer bias.

The proportion of sterilised adult dogs was calculated for each secondary survey and the weighted mean of these was the sterilisation coverage estimate for that site in that primary period (SI.1.3). Juveniles were excluded to give a more consistent sterilisation coverage estimate, as their numbers fluctuated seasonally (Fig. S2). Mark-resight surveys were conducted at a single point (limited by resources and to minimize disruption of FRDs) to estimate FRD population size and evaluate the detection rate of count surveys (Table 1, SI.1.5).

**Fig. 2:**
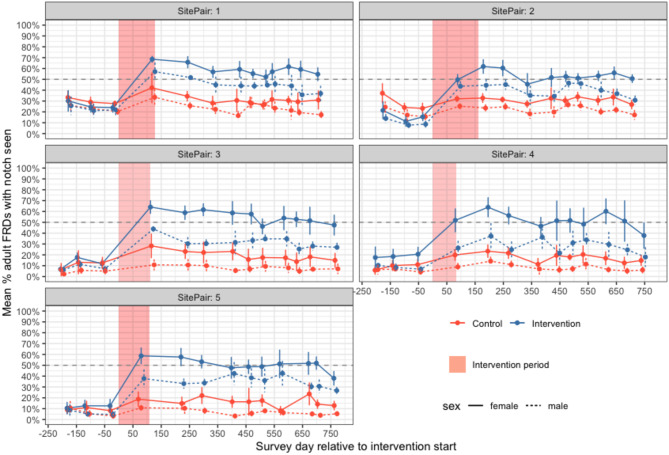
Sterilisation coverage of adult free-roaming dogs (female—solid line, male—dashed line) in paired study sites showing increases in coverage after the sterilisation intervention (red shading). Points and error bars show the weighted mean and standard deviation of secondary sampling surveys for each primary period with control sites in red and intervention sites in.

After three primary periods of surveys, sterilisation began on a staggered basis in November 2020 (Fig. 2, Fig. S1). Dogs were captured using butterfly nets or by hand and surgically sterilised (ovariohysterectomy or bilateral orchidectomy) and ear-notched under total intravenous anaesthesia (Propofol with Diazepam) at field clinics close to each study site and returned to their capture location when recovered (Fig. 1, further surgical details in SI.1.1).

### Statistical models for proportions and animal counts

A series of generalised linear mixed models (GLMM) with a Poisson distribution assessed FRD counts for adults, juveniles, puppies, unsterilised males and females, and sterilised males and females, using route length (kilometres) as an offset (Table S3, SI.1.4^44^). With dog counts as our response variable and the total dogs observed (of the same age group) as an offset, we used additional Poisson GLMMs to assess changes in the proportions of: female juveniles and adults, lactating females, underweight adults (BCS 1 or 2) and juveniles and adults with a skin condition (e.g. if female adult count was the response variable, total adult count was the offset, Table S3). To compare differences in control and intervention sites over time, all models included an interaction between time (days post-intervention) and site type (i.e. intervention or control). To allow the study sites to differ in their counts, we included random intercepts for each site. To allow the effect of intervention over time to differ between sites, random slopes were considered for all models but only included if their presence did not result in singularity errors (Table S3). If the data were overdispersed (dispersion ratio > 1), an observation-level random effect was added^45^.

In the interests of model parsimony and whilst retaining variables that were integral to answering our research question (i.e. site type and time) and random effects, we performed a manual forwards stepwise procedure to select from a subset of 4–5 biologically plausible covariates (time of day, month, rain, monsoon season, dog density when applicable; Table S3), using change in Bayesian information criterion (BIC) value to select variables. This information criterion approach is preferable to selecting variables based on statistical significance alone and we believe the limited number of candidate variables reduced noise sometimes associated with many indiscriminate variables^46^. We used the BIC value for model selection criteria to advantage more simple models, due to the already complex structure of the model, however in most cases, the Akaike information criterion and BIC values were similar. We acknowledge the potential risk of bias in using model selection, however, we believe the manual selection process, limited number of candidate variables, and the use of an information theoretic approach likely reduced the potential bias associated with this approach^47^. Estimates presented are the exponentiated coefficients, described as Incidence Rate Ratios (IRR). Due to the interaction over time with site type (control or intervention), IRR_intervention_ values represent the exponentiated coefficient over time compared to the control sites (IRR_control_).

Whilst GLMMs allowed easy interpretation and quantification of effects, we additionally used General Additive Mixed Models (GAMMs) to assess the non-linear effect of time on dog counts. The same covariates were used as in the equivalent GLMM, but the categorical variable for month was removed and replaced by a non-parametric smooth over the time variable^48^ (SI.1.4). To test the significance of the site type term (intervention/control sites), we compared the residual deviance of GAMMs with and without the site type variable using an analysis of variance (ANOVA). If the decrease in deviance after removal of the term was considered extreme for the Chi-squared distribution (*p* < 0.05), the term can be considered significant, as has been used to monitor populations of other species^49,50^.

### Community perceptions, cared-for dog surveys and statistical models

Residents in site pairs 1, 2, and 5 were questioned about their perceptions of FRDs via door-to-door surveys prior to the intervention (Q1, Fig. S1, sampling details in SI.1.6, questions in SI.5.4). Informed consent was obtained from all the participants. Participants consenting to a second post-intervention questionnaire (Q2) were telephoned 344–359 days after interventions (mean = 349 days). Questionnaires in control and intervention sites were performed at a similar time. Participants were asked about their opinions, feeding and sterilisation of FRDs, and if they perceived the FRD population had changed in terms of body condition, wounds, barking, number of puppies and total number of dogs. Participant’s responses were analysed with a series of proportional odds ordinal mixed regression models^51^ (SI.1.6). Covariates were tested as ordinal and nominal covariates and models were compared using the log likelihood. Similar log likelihood values showed no evidence that the proportional odds assumption was violated for our models^52^. As abandonment of cared-for dogs and puppies is thought to contribute to the FRD population, residents in all sites were questioned about dogs they cared-for; we recorded the age, sex, sterilisation and lactation status of dogs in the household after interventions (Fig. S1) and the proportion of sterilised cared-for dogs was calculated (SI.1.7). The R environment was used for all data curation and analysis^35^, additional package details in SI.1.

Ethical approval was obtained for the study from the Royal (Dick) School of Veterinary Studies Veterinary Ethical Review Committee (VERC 07.20) and the Human Ethics Review Committee (HERC 496–20, 511–20) at the Easter Bush Campus, University of Edinburgh. State government approval was obtained from the Government of Goa Department of Animal Husbandry and Veterinary Services and local approval was obtained from individual municipalities and panchayats. All procedures were performed in accordance with relevant local guidelines, regulations and recommended veterinary practice. The study is reported in accordance with the ARRIVE guidelines.

## Results

### Sterilisation coverage

Mean sterilisation coverage in study areas prior to the sterilisation intervention was 17.2% (SD = 8.1, Table 1). Unpublished state-wide data collected during rabies vaccination programmes^37^ suggests this is typical for Goan FRD populations. A total of 3222 dogs were sterilised in intervention sites (53.3% female; Table S2) over a mean intervention period of 119 days per intervention site (SD = 29 days). Of the female FRDs sterilised, 12.2% (95% confidence interval (CI) 10.9–13.7%) were in early pregnancy and 2.0% (95% CI 1.5–2.8%) were in oestrus. After the interventions, sterilised female adults were 2.53 times more likely to be seen (per unsterilised female) in intervention sites compared to control sites (95% CI 2.08–3.08, Fig. S3, Table S4). The highest adult female sterilisation coverage observed in intervention sites in the first or second period post-sterilisation were 65.8%, 61.8%, 64.1%, 63.9% and 57.6% for intervention sites 1–5 respectively (Fig. 2, Table 1). Sterilised female adults per unsterilised female, a proxy for sterilisation coverage, declined by 13% over the study period in control sites (IRR_control_ = 0.87, CI 0.79–0.95, Table S4) and this did not differ from the rate of decline in intervention sites (IRR_intervention_ = 1.02, CI 0.90–1.15).

### Estimated animal counts distinguished by age, sex and sterilisation status

**Fig. 3:**
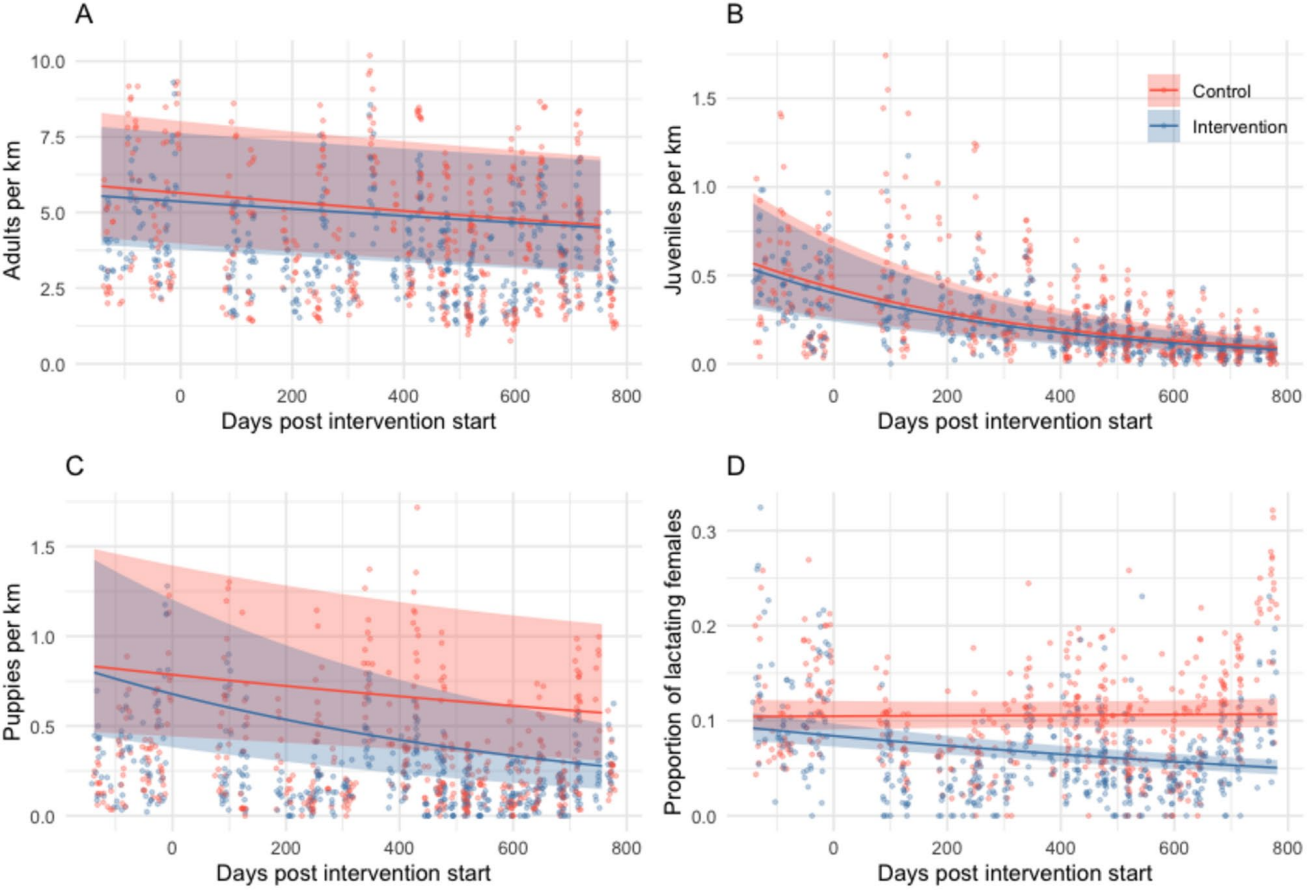
Free-roaming adult (**A**), juvenile (**B**) and puppy counts (**C**), and the proportion of lactating females (**D**), predicted from generalised linear mixed models with 95% confidence intervals (shaded areas) for sterilisation intervention (blue) and control sites (red) over time. Points show observed counts for each secondary survey with corresponding colours. Predictions are based on an average of all random effects for morning surveys, without rain or monsoon and in January (where those variables are present in the models, Table S3). Estimated coefficients in Table S5.

Over the study (comprising 3617 surveys), sightings of adults declined by 12% in control sites and by 10% in intervention sites with no significant difference between intervention and control sites (IRR_control_ = 0.88, CI 0.83–0.94; IRR_intervention_ = 1.02, CI 0.93–1.11; Fig. 3, Table S4). Sightings of juveniles per km of road surveyed substantially declined by 59% in control sites and by 60% in intervention sites over the study period with no significant difference between intervention and control sites (IRR_control_ = 0.41, CI 0.36–0.47, IRR_intervention_ = 0.97, CI 0.81–1.16, Fig. 3, Table S5). Puppies per km decreased by 17% in control sites (IRR_control_ = 0.83, CI 0.71–0.98) and by 40% in intervention sites (IRR_intervention_ = 0.72, CI 0.57–0.90, Fig. 3, Table S5). The number of lactating females per female adult (i.e. proportion of lactating females) significantly decreased in intervention sites by 26% over the study period but increased by 1% in control sites (IRR_control_ = 1.01, CI 0.94–1.09, IRR_intervention_ = 0.73, CI 0.66–0.82, Fig. 3, Table S5).

**Fig. 4:**
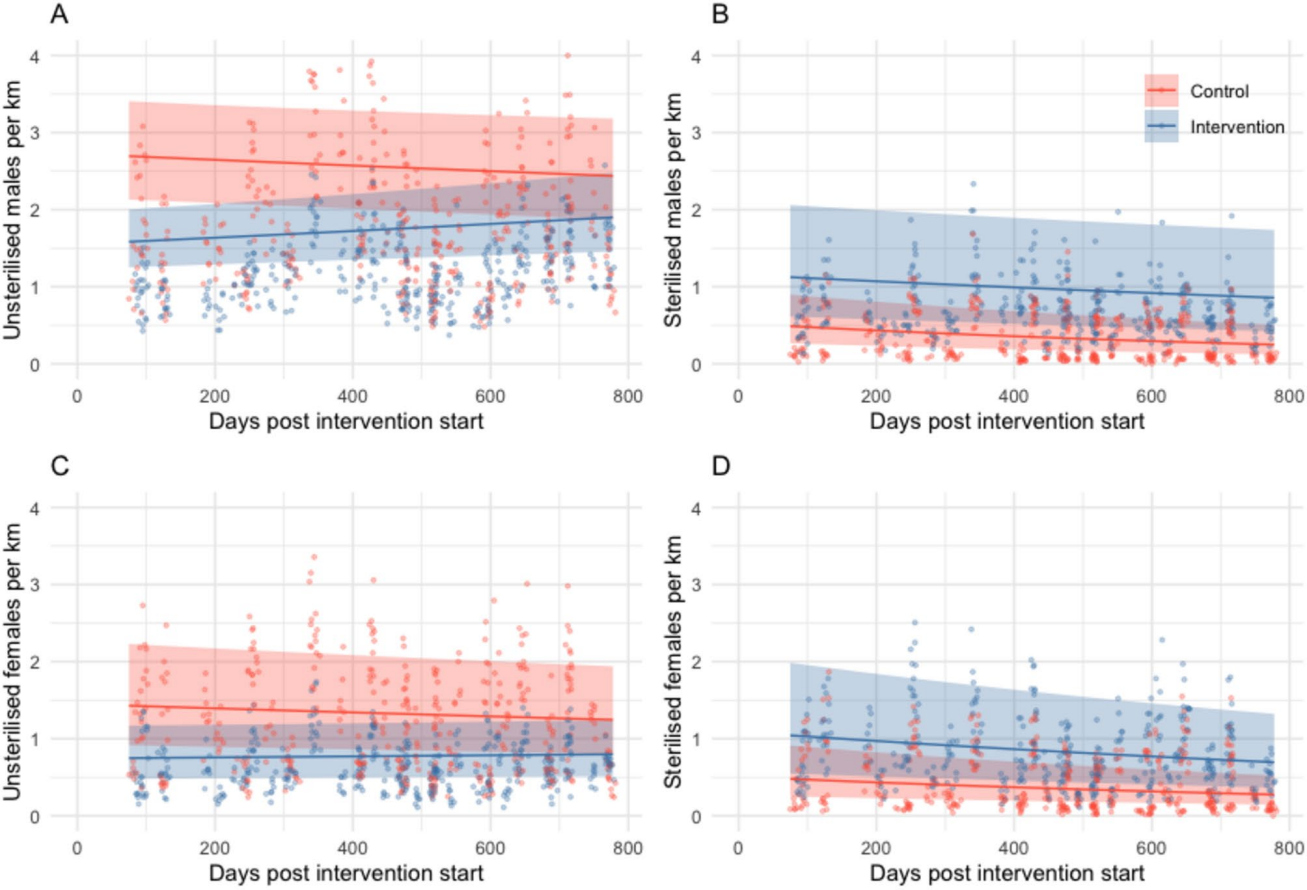
Adult free-roaming dog counts per km by sex and sterilisation status predicted from generalised linear mixed models with 95% confidence intervals (shaded areas) for sterilisation intervention (blue) and control sites (red) over time. Panels show (**A**) Unsterilised males, (**B**) sterilised males, (**C**) unsterilised females and (**D**) sterilised females. Points show observed counts for each secondary survey with corresponding colours. Predictions are based on an average of all random effects for morning surveys, without rain or monsoon and in January (where those variables are present in the models, Table S3). Estimated coefficients in Table S6.

Though absolute proportions of unsterilised dogs (1—sterilisation coverage) were consistently higher in control sites c.f. intervention sites (Fig. 2 & S3), there were differences between the trends in counts of unsterilised dogs post-intervention in the GLMM analysis. After the initial reduction in unsterilised adults in intervention sites (due to the intervention) there was a trend for significantly increasing counts of unsterilised males and females, compared to a decline seen in control sites during the same period (Fig. 4, Table S6). Unsterilised males per km decreased by 7% in control sites (IRR_control_ = 0.93, CI 0.87–1.00) and increased by 13% in intervention sites between the end of the intervention and the end of the follow-up period (IRR_intervention_ = 1.22, CI 1.11–1.35). Unsterilised females decreased by 9% in control sites (IRR_control_ = 0.91, CI 0.85–0.97) and increased by 5% in intervention sites between the end of the intervention and the end of the follow-up period (IRR_intervention_ = 1.15, CI 1.05–1.26). In the GAMM analysis, site type changed the model significantly only for unsterilised males.

Sterilised males in control sites declined by 38% (IRR_control_ = 0.62, CI 0.53–0.72; Fig. 4, Table S6) at a similar rate to sterilised females (33%, IRR_control_ = 0.67, CI 0.59–0.77). In intervention sites after the intervention, sterilised females declined by 26%, a rate not significantly different to that in control sites (IRR_intervention_ = 1.11, CI 0.93–1.32), whereas sterilised males declined by 18% in intervention sites (IRR_intervention_ = 1.33, CI 1.09–1.63), a significantly less steep decline than seen in control sites.

The GAMM analyses detected non-linear seasonal variation in the data (Fig. S12 and S13, Tables S9, S10). Including site type (intervention or control) in the adult, juvenile and unsterilised female and sterilised female models did not result in a significant change in residual deviance, therefore the term was considered not significant (Table S11). In the puppy, lactating female adult, unsterilised male, and sterilised male models the inclusion of the site type term resulted in significant change in residual deviance, suggesting a significant difference in FRD counts between intervention and control sites for these groups (Table S11). The GAMM and GLMM approach gave consistent results in all FRD counts measured except unsterilised female FRD counts, which were significantly different between intervention and control sites in the GLMM but not in the GAMM. Although we detected significant relationships within the data, the GLMMs had low conditional R^2^ values of less than 0.4 (Tables S4–S8), suggesting that the model selection process did not inflate this value substantially. The GAMMs had improved R^2^ scores of 0.72 to 0.90 (Table S9 and S10), which suggests that accounting for non-linear trends in FRD counts was beneficial.

### Population structure

Population structure was similar between all sites with adult dogs accounting for most of the population and a skew towards males in adult populations (mean proportion female = 0.38, SD = 0.02; Fig. S4). Extrapolating from count survey data and household survey responses (SI.1.7), 74% of the population was classed as free-living dogs, without a human guardian (SD = 14.1%), though this was lower in more rural sites (Fig. S5). The detection rate of adult FRDs on count surveys was 0.21 (SD = 0.08) based on adult population estimates from mark-resight surveys (Table 1) and this varied between sites and times of day (Fig. S6). Dog sightings had a seasonal pattern most evident in puppies (most likely to be observed December-January), then juveniles (increased December to April), and some seasonality was seen in adult dogs where there were more likely to be seen in hotter, drier months on morning surveys (Fig. S7).

Across all sites, we questioned 2670 households that cared for a dog. Most cared-for FRDs were adopted from the street in the local area (mean of all sites = 77.6%, SD = 6.5%) and 95.9% of these were adopted from the street when under one year old (SD = 3%). Some FRDs were imported from outside the local area, (the panchayat or municipal area; mean = 8.3%, SD = 4.1). For dogs with guardians, the mean sterilisation coverage post-intervention in control sites was 40.4% (SD = 4.3%) in confined dogs and 39.3% (SD = 20%) in roaming dogs and in intervention sites was 52.5% (SD = 17.5%) in confined dogs and 53.8 (SD = 11.9%) in roaming dogs. The difference in sterilisation coverage between intervention and control sites was only significant in pair 4 (Fig. S8). Further results in SI.2.6.

### Body condition

Surveyors reported ideal body condition in 87.63% of sightings (CI 87.47–87.79%, n = 144,493), with fewer sightings of emaciated (0.75%, CI 0.71–0.8, n = 1242), underweight (7.94%, CI 7.81–8.07, n = 13,090), overweight (3.57%, CI 3.48–3.66, n = 5887) or obese dogs (0.11%, CI 0.09–0.13, n = 179). The mean proportion of underweight dogs in control and intervention sites were similar prior to interventions and throughout the study (Fig. S14). The estimated proportion of underweight adults (underweight adults per total adult count) decreased from 9.11% (CI 7.48–11.1) to 3.57 (CI 2.97–4.29%) in control sites and from 8.52% (CI 6.93–10.47%) to 2.77% (CI 2.29–3.34%) in intervention sites over the study period (Model 10, Table S7, Fig. S14). This decline was not significant over time in control sites (IRR_control_ = 0.91, CI 0.76–1.08) but was slightly steeper in intervention sites (IRR_intervention_ = 0.91, CI 0.83–1.00). Surveyors were more likely to observe unsterilised dogs as underweight or emaciated compared to sterilised dogs. This difference was most apparent in the first year of the study but was statistically significant across the study years (year 1 difference = 11.28, 10.69–11.86; year 2 difference = 4.61, 4.23–4.97; year 3 difference = 3.89, 3.49–4.28; Fig. S9). Lactating adult female dogs were more likely to be observed as underweight or emaciated compared to non-lactating females, which was also most apparent in the first year (year 1 difference = 24.12, 22.05–26.19; year 2 difference = 13.99, 12.2–15.77; year 3 difference = 9.77, 8.08–11.46; Fig. S9).

### Community perceptions

**Fig. 5:**
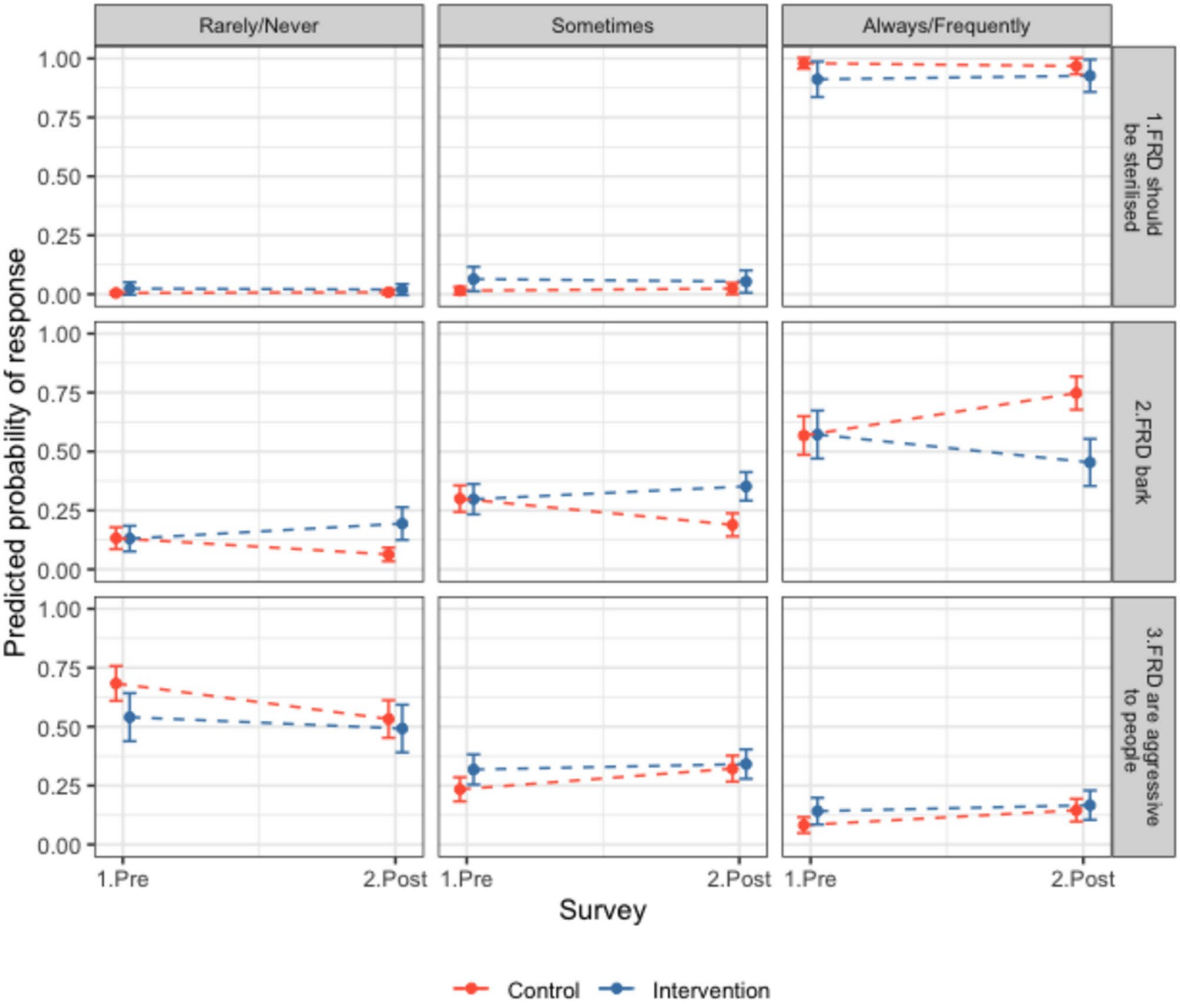
Community opinions of free-roaming dogs. Responses of residents in intervention (blue) and control (red) sites to questionnaires before and after the intervention (surgical sterilisation) regarding free-roaming dogs (FRDs) in their area. Participants were asked to choose between always, frequently, sometimes, rarely or never regarding the following statements; 1. ‘FRDs should be sterilised’ 2. ‘FRDs bark in my area’ 3. ‘FRDs are aggressive to people in my area’. The predicted probability of response from ordinal models for individual questions is shown, e.g. an increase in the predicted probability of response in the ‘always/frequently’ panel for statement 2 from pre to post can be interpreted as more participants answering that they thought FRDs barked always or frequently in their area. Questionnaire counts in Table S12 and model outputs in Table S14. Further results in SI.2.5.

Responses to questionnaires both before (Q1) and after sterilisations (Q2) were obtained for 334 households within site pairs 1, 2, and 5 (intervention sites n = 132, control sites n = 202, response rate SI.2.5). Consistently, most participants reported that FRDs should frequently or always be sterilised (91%, CI 88–93%, Fig. 5). In intervention sites in Q2, only 27% of respondents to community perceptions surveys said they were aware of sterilisation campaign (n = 36) and all either agreed or strongly agreed (44.4 and 55.5% respectively) that FRD should be sterilised in their area.

**Fig. 6:**
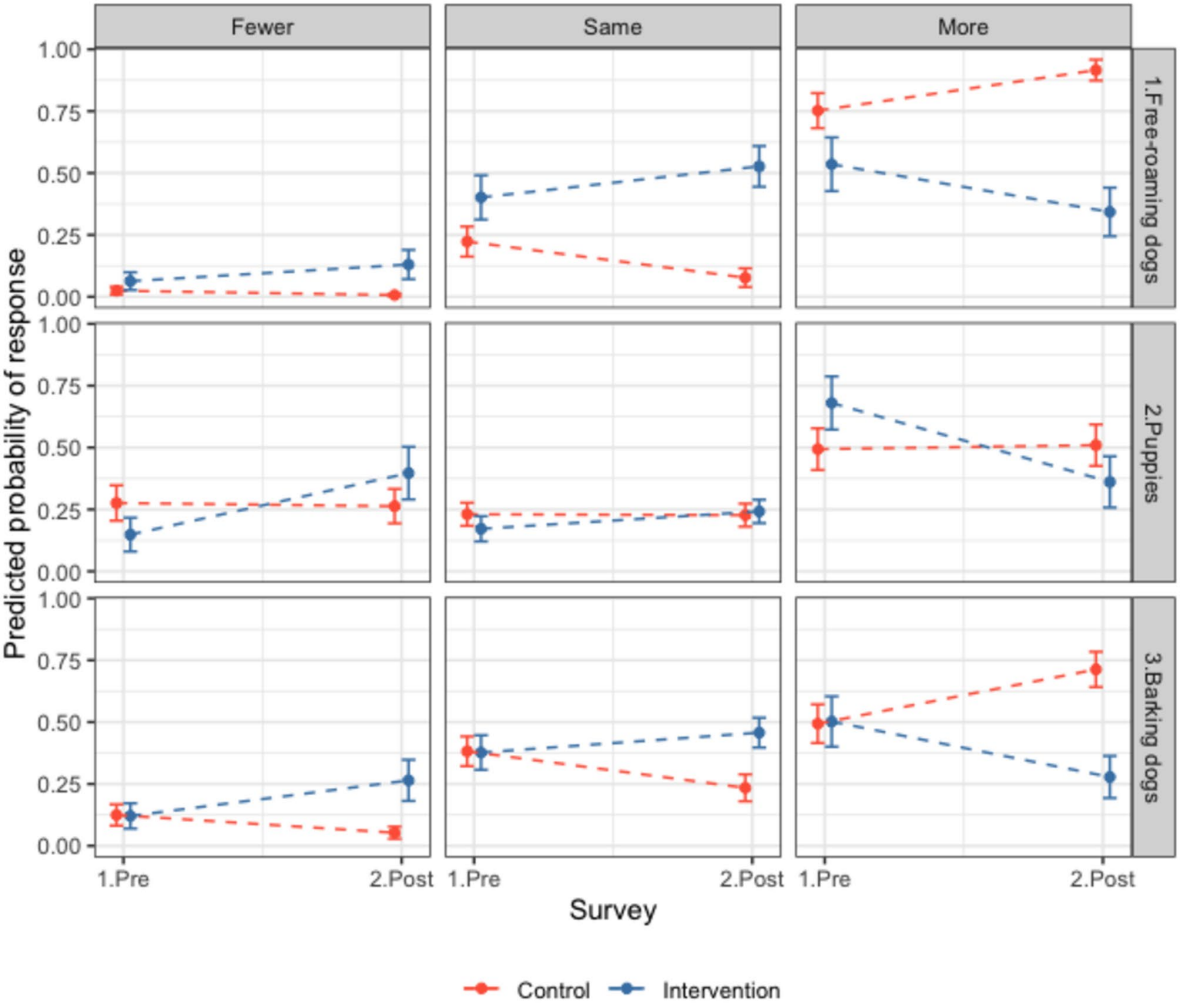
Community perceptions of free-roaming dog numbers. Responses of residents in intervention (blue) and control (red) sites to questionnaires before and after the intervention (surgical sterilisation) regarding free-roaming dogs (FRDs) in their area. Questions presented are ‘Compared to a year ago, have you noticed fewer, the same or more: 1. FRDs in your area? 2. Puppies in your area? 3. Barking FRDs in your area?’ The predicted probability of response from ordinal models for individual questions is shown, e.g. an increase in the predicted probability of response in the question 1 ‘more’ panel from pre to post can be interpreted as more participants answering that they noticed more FRDs. Questionnaire response counts in Table S13 and model outputs in Table S15. Further results in SI.2.5.

In Q2 in intervention sites (post-sterilisation) there was a trend towards observing fewer dogs; fewer participants reported seeing more puppies and dogs, and more participants said they saw fewer or the same number of puppies and dogs compared to the control group (OR all FRDs = 0.12, CI 0.05–0.28, OR puppies = 0.24, CI 0.11–0.53, Fig. 6, Table S15). In control sites in Q2, more participants reported seeing more FRDs (OR = 3.62, CI 2.03–6.46) but similar numbers of puppies (OR = 1.07, CI 0.69–1.65, Fig. 6). More participants in the intervention sites in Q2 said there were fewer barking FRDs and fewer participants said there were more barking FRDs compared to the previous year (OR 0.15, CI 0.07–0.3), and the opposite effect was seen in control sites (OR = 2.55, CI 1.65–3.94, Fig. 6). In addition, more participants reported FRDs barking rarely or never and fewer people reported them barking frequently or always in the intervention sites after sterilisations compared to the control sites (OR = 0.28, CI 0.14–0.55, Fig. 5, Table S14). Most responses in control and intervention sites reported FRDs being rarely or never aggressive to people (0.56, 95% CI 0.52–0.6, Fig. 5). Control participants were more likely to report aggressive behaviour happening frequently or always in Q2 compared to Q1 (1.9, CI 1.25–2.89), whereas intervention site participants’ responses were consistent between surveys (OR = 0.64, CI 0.33–1.24, Fig. 5). Participants reported that ‘Stray dogs in this area are a problem’ sometimes (41%), frequently (13%) or always (12%), and 35% reported stray dogs were rarely or never a problem (Table S12). There were no significant differences between the Q1 and Q2 or between control and intervention groups. Overall, most people reported being rarely or never scared of FRDs (proportion = 0.63, CI 0.59–0.66, Table S12). In Q2, participants from intervention sites were more likely to say they were frequently or always scared of FRDs (OR = 2.29, CI 1.15–4.55, Table S14) and control sites were less likely to report this (OR = 0.63, CI 0.4–0.99, Fig. S15). Further results reported in SI.2.5.

## Discussion

The unique design of this controlled study has allowed us for the first time to rigorously assess the real-world impacts of sterilisation of FRDs in multiple sites. We observed a reduced fertility rate in intervention sites but no reduction in FRD adult counts compared to control sites within the follow up period, despite achieving female sterilisation coverages of 58–66% during a one-off sterilisation campaign. Here, we discuss the possible reasons for these observations and how they might inform future dog population management strategies.

### Population dynamics

Statistically significant reductions in puppies and lactating females in intervention sites demonstrated that interventions reduced birth rates. Puppy numbers may have been affected soon after interventions due to 14% of female dogs that were in oestrus or pregnant at surgical sterilisation. However, the largest impact was expected in the first puppy season after interventions in December 2021/January 2022. These puppies would grow to adulthood by December 2022/January 2023 (our last survey point); therefore we expected to see the largest impact on the adult population by this time. Total adult dog counts across both control and intervention sites decreased over the study period, but differences between the two types of sites were not observed. We suggest that the study populations had low turnover because the sterilised fraction of the population remained relatively constant post-intervention. Low turnover is often associated with high juvenile mortality and limited recruitment into the adult population^53,54^, therefore despite a probable reduction in juvenile and puppy mortality, so few juveniles would have lived to become adults anyway that the reduction was not sufficient to alter adult recruitment.

Several control sites had plans for future sterilisations and project resources were limited, so monitoring past 26 months was not possible. We cannot rule out changes in the adult population in intervention sites after the study ended, however there was no indication in the data that this was likely. Whilst important, reducing FRDs is not the only aim of dog population management, and fewer females experiencing the high-risk times of mating, pregnancy and lactation and a reduction in deaths of young dogs is likely to have improved overall welfare of dogs in intervention sites. Reduced population turnover is of benefit to maximizing rabies control due to vaccinated dogs persisting in the population for longer, lower birth rates of unvaccinated puppies^55^, and fewer dispersing juveniles potentially spreading disease^39^. We found no significant change post-intervention in the number of participants that considered FRDs a problem, yet participants in the intervention sites reported less barking. Contrary to our expectations, intervention site participants were more likely to be fearful of dogs after the intervention, perhaps due to a raised profile of FRDs during sterilisations. FRDs additionally have substantial impacts on human health, livestock and wildlife yet they were out of the scope of this study and have been addressed elsewhere^32^.

The lack of adult population change due to sterilisation in the present study contrasts with mathematical models predicting population reduction^24,26,56^. Differences include control sites with low sterilisation levels and varying estimates of migration. Empirical data, such as realistic capture rates and population estimates from studies like this, may improve the accuracy of mathematical models.

### Dog migration, behaviour and health

Study populations were open to migration (SI.Eq. 1), which led to confounding issues in quantifying the effect of our interventions, yet represents a common real-world scenario. Post-intervention, there was an increase in unsterilised adult male FRDs in intervention sites compared to control sites and an increase in unsterilised females was detected in the GLMM (but not the GAMM). This rise is likely due to immigration, not recruitment, as we observed declining puppy counts, especially in intervention sites. Hypothetical explanations for increased immigration include reduced territorial defence by sterilised dogs due to loss of gonadal hormones. In support of this theory, participants reported less barking—a sign of territorial behaviour^57,58^—in intervention sites. As this is one of the most reported problem behaviours associated with FRDs^10^, this reduction is of particular value to the local community. The impact of gonadal hormone removal on territorial behaviour and movements, especially in male dogs, needs further investigation. Similar patterns of immigration were observed in fertility-controlled FRD populations in Italy and Ukraine^59^ and foxes were more likely to share territory once sterilised^60^. Sterilised animals might therefore increase carrying capacity by reducing aggressive interactions^61^. There have been lower rates of dogs with wounds reported in cities with sterilisation programmes^62^, however the rates of wounds reported by surveyors in this study were negligible in control and intervention sites suggesting our study methods were not sensitive to observation of wounds. Importation of dogs is an unlikely explanation as community surveys indicated low levels of external dog importation. Abandonment is another possible cause of increased unsterilised dogs^26,27^ however, rates were expected to be similar across sites. Compensatory recruitment seems unlikely due to declining juvenile numbers in control and intervention sites and only modest population decline. We suggest that migration hampered the efficacy of the sterilisation intervention^63^. Studies of free-roaming cats suggest this might be mitigated with larger, contiguous intervention areas^29^. Fertility control is reported to cause less perturbation than culling^53^, however our study suggests that we should still consider the impacts of perturbation when planning sterilisation programmes. Contrary to expectations due to their reported increased longevity^64^, sterilised dog counts declined similarly to unsterilised dogs, suggesting possible emigration due to loss of territory^65^ or unwanted increased attraction to unsterilised dogs^66^, though no direct evidence was found in this study. The higher decline of sterilised males in control sites might indicate behavioural disadvantages. Eighty-eight per cent of FRDs were observed to be in ideal condition and 86% showed no skin issues. This aligns with European FRD studies (73% ideal^22^) but contrasts with reports of higher malnutrition rates in Northern Indian cities (70%^67^ and 71–88% underweight^62^). The difference may be due to regional attitudes toward FRDs, Goa’s anti-rabies vaccination program^37^, or its status as a tourist destination. Survey observations were supported by body condition assessment at sterilisation by a veterinarian, suggesting that the difference is not related to method.

Unsterilised and lactating dogs were more prone to being underweight, as has been identified previously^67^, particularly during the first year of the study likely due to scarce resources during the lockdown. Body condition improved as resources became more available. The general good health of FRDs in this study likely contributed to higher fertility and lower mortality rates. In contrast, less healthy populations may struggle more with maintaining adult recruitment and could be more impacted by fertility-reducing interventions.

### Limitations

The juvenile dog population decreased significantly in both control and intervention sites, likely due to external factors, not the intervention itself. The study began in May 2020, shortly after a strict COVID-19 lockdown in India^68^, which limited food availability for FRDs^69^ and possibly caused initial misclassification of underweight adults as juveniles. As FRD body condition improved, fewer juveniles were seen, suggesting misclassification rather than genuine changes in juvenile numbers. Further monitoring of these populations may clarify the typical ratio of juveniles to adults to confirm this.

Despite best efforts to match sites based on studying the local area and site walk-throughs, human population density (as reported by WorldPop^48^) differed between some paired sites, creating higher human:dog ratios in intervention sites of pairs 4 and 5. Long-term monitoring in Goa indicates a stable FRD population at carrying capacity statewide, however, more humans in these sites may have supported optimal FRD survival due to unrestricted resources. This could make sterilisation the primary population-limiting factor. In contrast, control areas with lower human:dog ratios may have been constrained by carrying capacity. However, no significant differences between intervention and control sites suggest this had minimal impact on results. Individual dog identification could facilitate further research on these dynamics. Microchips were used to identify individual dogs in this study, however logistical and technical issues with dog capture and scanning microchips prevented this data being collected in many cases. Future studies should consider remote individual identification methods using novel devices or photo recognition to reduce capture/recapture bias, reduce animal stress, and boost resight rates. Inclusion of births, deaths, immigration, and emigration in analysis through individual identification is likely to increase the precision of results and enhance our understanding of sterilisation impacts on population dynamics.

The sterilisation coverage achieved in our study sites was the highest that could be achieved within the intervention period, but it was not as high as studies documenting sterilisation programs with similar capture protocols in other locations, typically large Indian cities^19,20,70^. This may be due to environmental topology, i.e. dogs are easier to capture in predominantly urban environments^71^, or simply the length of time of the intervention, i.e. over years rather than months. Further work should identify methods to achieve high sterilisation coverage in non-urban settings, especially important as these regions have limited access to healthcare and present greatest risk of human rabies deaths^5^.

Though we encouraged sterilisation of cared-for dogs in intervention sites, we were unable to limit sterilisation of cared-for dogs in control sites, as has been previously described^13^. Sterilisation coverage in cared-for dogs was similar or more in control sites compared to intervention sites. These levels of sterilisation coverage may have reduced numbers of abandoned puppies in control sites and could mask population impacts that might have been apparent comparing intervention sites to a control population with no sterilised FRDs. However, the levels of sterilisation coverage in cared-for dogs in study sites were consistent with the general dog population of Goa, therefore we believe the control sites were an accurate representation of areas with no active FRD sterilisation in the region.

## Conclusion

This study, utilising a novel BACI framework, demonstrated that sterilising 58–66% of female FRDs through a one-off intervention resulted in fewer puppies and lactating females, but had no detectable impact on adult dog counts across multiple sites in the 2-year follow-up period. Reduced lactating females and puppy deaths likely contributed positively to FRD welfare, and a decrease in reported barking may have improved local residents’ lived experience. The apparent lack of effect on population size could be the result of immigration into and emigration out study areas, which would confound the estimates of population change. Yet movement of FRDs represents an important barrier to the efficacy of sterilisation interventions in the real world. Future studies applying more intensive and geographically contiguous sterilisation efforts over sustained periods may mitigate these movements, as identified in free-roaming cat populations^29^. The results of our study suggest that surgical sterilisation was an appropriate tool to limit birth rate, yet attempting to manage FRDs solely by this technique requires substantial investment, and even with this, may effect little change. There are numerous other factors governing the FRD population size that sterilisation of FRDs does not address, e.g. abandonment from the pet dog population, migration of FRDs from other areas, and resource availability^27,72^. The results of our study provide evidence to support WOAH’s recommended approach, which states that dog population management should attempt to address multiple factors affecting the FRD population, not only reproductive control, but also responsible dog ownership, controlled dog movements and breeding, waste management, access to veterinary care and education^17^. Stakeholders must clearly define sterilisation goals to avoid unrealistic expectations and resource wastage.

## Electronic supplementary material

Below is the link to the electronic supplementary material.

Supplementary Material 1

## Data Availability

The datasets used and analysed and selected R code used during the current study are available at 10.7488/ds/7919.
