# Supplementary material for: Managing free-roaming domestic dog populations using surgical sterilisation: a randomised controlled trial
Source: Sci Rep. 2025 Apr 24;15:14221. doi: 10.1038/s41598-025-98990-1 (PMC12022150; doi:10.1038/s41598-025-98990-1)
Supplement: Supplementary file 1 — Supplementary Material 1 [file 41598_2025_98990_MOESM1_ESM.pdf]

# Supplementary information - Managing free-roaming domestic dog populations using surgical sterilisation: a randomised controlled trial

## 1. Supplementary methods

### 1.1 Capture and surgical sterilisation protocols

*Capture*

Each intervention site was split into catching zones and catching teams were directed each morning or evening session to specific zones using a purpose-built smartphone app. Dogs estimated as >5kg, without evidence of previous sterilisation were captured using butterfly nets or by hand where possible using methods from a previous study [1]. Lactating females with puppies estimated <8 weeks old or heavily pregnant females were not captured as per methods in a previous study [2]. Households within intervention sites were offered free sterilisation for their dog and were collected separately.

*Surgical sterilisation*

Dogs were sedated with Xylazine and Butorphanol (xylazine hydrochloride 23.33 mg per ml, dosage: 2 mg/kg IM; Indian Immunologicals Limited, Hyderabad, India, and Butorphanol tartrate 2 mg per ml; dosage: 0.2 mg/kg IM; Butodol-2, Neon Laboratories Limited, Mumbai, India) approximately 15 minutes before induction of general anaesthesia. Total intravenous anaesthesia was administered (Propofol 1% w/v at 10 mg per ml; dosage: 1 mg/kg IV; Neorof, Neon Laboratories Limited, Mumbai, India) alongside diazepam (Diazepam Injection

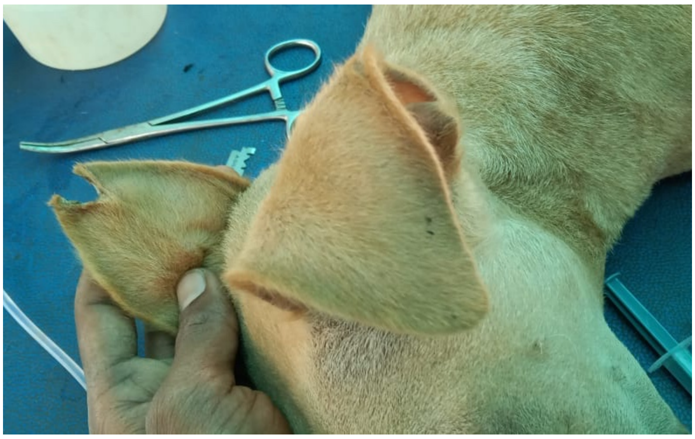

IP, contains: diazepam 1 mg per ml; dosage: 0.25 mg/kg IV, Lori, Neon Laboratories Limited, Mumbai, India). Ovariohysterectomy was performed to sterilise females with a ventral midline incision according to standard techniques and bilateral orchidectomy was performed to sterilise males with a midline prescrotal incision. All dogs had intradermal sutures. During anaesthesia, dogs had an ISO standard microchip implanted in the subcutaneous tissue along the dorsal midline between the scapula enabling individual identification and one ear was notched to indicate sterilisation (inset photo). Dogs were vaccinated against rabies (Nobivac Rabies, MSD Animal Health) and given ivermectin (0.2mg/kg SC, Embark Lifescience Pvt. Ltd., Rorkhee, India). A perioperative IV antibiotic

Amoxycillin and Salbactam was administered (contains: Amoxycillin 3000 mg and Sulbactam 1500 mg as 4.5 g per vial; dosage: 20 mg/kg IV; Vetoxy-sb 4.5 gm, Vet Science, Ahmedabad, India). Maintenance of the anaesthesia was achieved with a Propofol bolus (1 mg/kg IV) every 6–10 min to effect, based on jaw tone, eye position and palpebral reflexes. Some dogs were administered a combination of 1:1 Ketamine + Diazepam or diluted Xylaxine to control unstable anaesthesia if required (Ketamine Hydrochloride IP, 50 mg/ml, dosage: 5mg/kg; Aneket, Neon Laboratories Limited, Mumbai, India) (Figure 2). Post-operative antibiotics were not routinely administered, unless clinically indicated. Non-steroidal anti-inflammatory analgesia (Meloxicam injection IP, 0.1 mg/kg SC, Salcam, Vet Life Sciences, Ahmedabad, India) was given perioperatively, the day after surgery, and thereafter if necessary. At sterilisation the dog's health status (skin condition, body condition, wounds) and reproductive state (for females: pregnant, in oestrus, lactating) were recorded in a purpose-built smartphone app. If dogs were unsuitable for surgery (e.g., too old, ill-health), they were returned to their capture location or treated as necessary. Peri- and post-operative complications were monitored. Free-roaming dogs were kennelled after the procedure, monitored daily, and returned to their capture location when a veterinarian confirmed they were sufficiently recovered. Cared-for dogs were returned to their guardian after they had recovered from anaesthesia with meloxicam tablets (Metaflam chewable tablets 0.1-0.2 mg/kg, SAVA Healthcare Limited, Pune, India).

### 1.2 Count survey protocols

Surveys were performed concurrently in paired study sites to minimise differences in climatic and social factors. Seven surveyors completed all surveys, three remained for the duration of the study and there were four to five surveyors at any point in time. All surveyors were trained to a strict protocol based on protocols from International Companion Animal Management coalition [3, 4] with regular refresher training and shadowing by managers (materials in SI.5). Surveyors had vision tests to ensure optimal vision.

The survey routes that make up the site surveys were performed by different surveyors on the same day where possible and within a median of 1 day (interquartile range 0–2 days). Primary periods across a pair of sites typically spanned less than 5 days, but longer during periods of heavy rain, when sites were not accessible, or due to staff shortages (n = 30, 3.0 % of surveys). Each survey route was recorded (Strava Inc, 2022) and checked. A survey was deemed 'invalid' and removed from the study if the route deviated significantly from the designated route, if they were interrupted for more than 30 minutes (including interruptions by heavy rain), the start was delayed for over 30 minutes, or there were clear disturbances on the route, e.g. festivals or dog catching. All zones and survey routes were

mapped and measured in QGIS version 3.4 (QGIS Development team, 2019). Road networks were obtained from OpenStreetMap (https://www.openstreetmap.org).

Between May 2020 and January 2023, 3681 survey routes were completed and after removing invalid surveys, 3617 remained for analysis (Fig. S1). The first sampling period was removed from all analysis as this was likely to be less robust due to the inexperience of the surveyors. In the first two primary periods, we asked surveyors to primarily concentrate on major characteristics of dogs (sex, age, sterilisation status). This seemed to lead to abnormally low counts of skin conditions, so data from primary periods 1 and 2 were removed from the skin condition models to avoid bias of surveyor experience.

Body condition scores were dichotomised as underweight (scores 1 and 2) and ideal or overweight (scores 3, 4, 5) and the proportions of underweight dogs were compared between sterilised and unsterilised dogs and lactating and non-lactating females using the 2-sample test of equal proportions in R[6].

## 1.3 Sterilisation coverage estimation

The proportion of sterilised adult dogs was calculated for each secondary survey for males, females and both sexes combined. Using the stats weighted.mean() function in base R[6], the weighted mean sterilisation coverage was calculated from the proportion of sterilised dogs in secondary surveys within a primary period, weighted by the total number of that group that were sighted on each survey (see Code supplement).

## 1.4 Analysis of counts using GLMMs and GAMMs

We expected the dynamics in the study populations to approximate the following equation:

$$\text{(Eq. 1) } n_{\{after, A\}} = n_{\{before,A\}} - deaths_{\{A\}} + births_{\{A\}} - emigration_{\{A\}} + immigration_{\{A\}}$$

, where A denotes a study site designated as either control or intervention and

$$\text{(Eq. 2) } births_{\{A\}} = f_{\{A\}} * n_{\{adult\ females,\ A\}}$$

, where $f_{\{A\}}$ is birth rate for site A. Adult recruitment rate is defined as:

$$\text{(Eq. 3) } (n_{\{adults,\ A,t+1\}} - n_{\{adults,\ A,t\}}) / n_{total\ population\}}$$

This is the first randomised, controlled trial to monitor FRD population size longitudinally in five blocks, where sterilisation of FRDs is applied to the intervention site, the aim of which is to lower $f_{\{sterilisation\}}$ compared to $f_{\{control\}}$ in the same block.

Several Generalised linear mixed models (GLMMs) were fitted with dog counts as the response variable and varying offsets (Table S3) using the R package lme4 and the function glmer with a Poisson distribution and log link function [7]. The mean counts (on the log scale) are specified in the following equation:

$$E[\log(Adults_{\{i,j,t\}})] = \log(RouteLength_{\{i,j,t\}}) + Site_i' + ID_{\{i,j,t\}}' + \beta_1\, dp + \beta_2\, SiteType + \beta_3\, (dp*SiteType) + \beta_4\, am\_pm + \beta_5\, month + \beta_6\, (am\_pm * month) + \beta_7\, rain + \beta_8\, monsoon + \beta_9\, (rain*monsoon)$$

where:

- i denotes site
- j the group (intervention or control)
- t is the time of the survey
- Site is a random effect for the study site and ID is a random effect for the survey at a given site and time
- dp is the number of days post-intervention (for an intervention site or its paired control)
- SiteType is an indicator variable for the treatment (1 for intervention and 0 for control)
- am_pm, rain, and monsoon are indicator variables for afternoon survey, rain occurring, and monsoon season, respectively
- month is a categorical variable (Jan, Feb,...,Dec)
- coefficient $\beta_2$ is the main effect of sterilisation on counts on the log scale (with adjustments based on dp)

All model predictions are presented for an average of all intervention or control study sites, in January, morning surveys, not in monsoon season and without rain, unless otherwise indicated. Tables of results produced using sjPlot [8]. Percentage change in dog counts for intervention groups are calculated by multiplying the exponentiated coefficients for Days post intervention (control sites) and the exponentiated coefficents for the interaction between days post intervention and intervention sites.

In addition to the GLMMs, we used General Additive Mixed Models (GAMMs) to assess the non-linear effect of time (dp) on counts. The same covariates were used as in the equivalent GLMM, but the categorical variable for month was removed and replaced by a non-parametric smooth over the time variable (dp, number of days post-intervention). The model was specified according to the following equation:

$$E[\log(Adults_{\{i,j,t\}})] = \log(RouteLength_{\{i,j,t\}}) + Site_i' + \beta_1\, SiteType + \beta_2\, s(dp, by = SiteType) + \beta_3\, am\_pm + \beta_4\, month + \beta_5\, (am\_pm * month) + \beta_6\, rain + \beta_7\, monsoon + \beta_8\, (rain*monsoon),$$

where:

- i denotes site,
- j the group (intervention or control)

- t is the time of the survey
- Site is a random effect for the study site. s(dp, by = SiteType) indicates the smoothed term for the number of days post-intervention (for an intervention site or its paired control)
- SiteType is an indicator variable for the treatment (1 for intervention and 0 for control)
- am_pm, rain, and monsoon are indicator variables for afternoon survey, rain occurring, and monsoon season, respectively
- month is a categorical variable (Jan, Feb,...,Dec)
- coefficient $\beta_2$ is the main effect of intervention on counts on the log scale (with adjustments based on dp)

The level of smoothing of dp was chosen to best reflect long-term data trends rather than smaller fluctuations between observations, therefore lower degrees of freedom were preferred [9]. The Poisson GAMMs were overdispersed, therefore the standard errors were corrected using a quasi-GAMM model [10]. Smooth terms in GAMMs were estimated using automatic selection of penalised regression in the R package 'mgcv' [11].

## 1.5 Mark resight surveys

To estimate the population of dogs in each site and the detection rate of count surveys, we performed a mark-resight survey between May and November 2022 alongside routine vaccination activities. A hand vaccination team (1 bike, two vaccinators) vaccinated and marked FRDs (with non-toxic paint) in a designated zone on day 1, a net-catching vaccination team (1 truck, 3-4 animal handlers/vaccinators) covered the zone on day 2, all wore yellow polo shirts. On day 3 a surveyor from a different team, wearing plain clothes on a single bike (as per dog count surveys) surveyed the zone once in the morning and once in the afternoon. The Chapman method [12] was used to estimate population size. To use data from both surveys, a weighted estimate was calculated (E*), based on a linear combination of the two estimates, where the weights are inversely proportional to the variances:

$$w = V2/(V1+V2)$$
$$E^* = w * E1 + (1-w)*E2,$$

where E1 and E2 are the 2 Chapman estimates and V1 and V2 are the corresponding variances. It is possible that the disturbance of vaccination teams caused changes in the FRDs behaviour and marked dogs might be more likely to avoid the area of capture after being captured, this could result in overestimation of the population. To minimise the risk of this, we made sure surveys were performed at least 24 hours after marking, to allow time for the dogs to recover. Surveyors were different people to the capture teams, wore plain clothes and used different vehicles so they would blend in with local residents.

The weighted estimates were used as a denominator to calculate the survey detection rate (DR) for the two count surveys that occurred either side of the mark-resight survey.

$$DR = n_{adultR}/E^*,$$

where $n_{adultR}$ is the mean number of adults counted at the Rth survey round. A Welch two-sample t-test was used to compare detection rates between morning and afternoon surveys.

## 1.6 Community perceptions questionnaire protocols and ordinal models

To record community perceptions of FRDs, we sampled households using a 2-stage selection procedure. First, each site (except site pairs 3 and 4 as COVID-19 restrictions prevented surveys) was split up into multiple zones and sample zones were selected by random number generation. Selected zones were surveyed until 400 surveys had been completed or when no further time was available. All houses were called upon for perceptions surveys until sufficient surveys were collected.

Participants that gave their consent at the in-person questionnaire (Q1) were called back after the intervention for the second follow-up questionnaire (Q2), allowing us to directly match participants between questionnaires. The questions were the same between Q1 and 2 apart from omitting participant gender, age, education, and religion from Q2 as we did not expect these to change over time. Demographic differences between all Q1 respondents and respondents of both surveys and differences in the proportion of dogs with a human guardian between paired sites were compared with a test for equality of proportions.

The co-authors resident in Goa considered that 'stray' was a locally appropriate term to describe FRDs in the survey and the definition was clarified at the start of the survey: 'stray dog refers to any dog roaming on the street that is NOT on a leash or chain, whether it has an owner or not'.

*Community perceptions of FRDs: Ordinal models*

Responses on the ordinal scale were analysed using the clmm function from the R package 'ordinal' [13] according to the following model equation:

$$\text{logit}(P(Y_{i,j,t} \le k)) = \theta_k - \beta_1(\text{survey\_number}_i) - \beta_2(\text{SiteType}_i) - \beta_3(\text{survey\_number}_i \times \text{SiteType}_i) - \beta_4(\text{dog\_owner}_i) - u(\text{UID}_i)$$

$$i=1,...,n,\ j=1,...,J-1,$$

where

- i denotes Site
- j the group (intervention or control)
- t is the survey number (Q1 or Q2)
- $P(Y_{i,j,t} \le k)$ represents the probability that observation i,j,t falls into category k or below.

- $\theta_k$ is the threshold parameter for category k.
- $\beta_1$, $\beta_2$, and $\beta_4$ are coefficients for survey_number, SiteType, and whether participant is a dog owner respectively.
- $\beta_3$ is the coefficient for the interaction term between survey_number and SiteType.
- $u(UID_i)$ is the error term associated with the observation.

We assume that the individual participant (UID) effects are IID normal: $u(UID_i) \sim N(0,\sigma_u^2)$.

All models follow the same basic framework as the above equation, including survey_number (Q1 or Q2), SiteType (control or intervention) and their interaction and a participant-level random effect. Covariates describing whether the participant was a dog owner and whether the participant was aware of the intervention were added in a stepwise manner and the model with the lowest AIC was chosen.

### 1.7 Cared for dog questionnaires

To obtain information about cared-for dogs, we sampled households using the same 2-stage selection procedure as described above for community perceptions questionnaires (Q1, Appendix SI.5.4). As we were unable to question residents of site pairs 3 and 4 in Q1, we did another in-person survey after interventions in all sites and we present the results from this questionnaire regarding cared-for dogs.

*Estimating the proportion of cared-for dogs*

We assumed dogs with collars not placed by NGOs were cared-for. We asked in the survey if the dog they care for wore a collar to calculate the proportion of cared-for dogs wearing a collar.

$$prop_{caredfor\_collar} = n_{cared\text{-}for\ dogs\ with\ collar}/n_{cared\text{-}for\ dogs}$$

$$f = prop_{caredfor\_collar}/prop_{pop\_collar}$$

$$prop_{caredfor\_uncollared} = prop_{uncollared} / f$$

$$prop_{caredfor} = prop_{caredfor\_uncollared} + prop_{pop\_collar},$$

where $prop_{pop\_collar}$ is the proportion of dogs observed in the population with a collar.

## 2. Supplementary results

### 2.1 Surgical Sterilisation

Of 3237 captured dogs, 15 surgeries were not performed due to veterinarians classing the dogs as sick (n=9), already sterilised (n=1), too old (n=2), or too young (n=3). Fifteen dogs escaped from the clinics

and there was a 0.01% surgical complication rate (perioperative: n = 11, post-operative: n = 20) and the mortality rate of dogs in the clinic was 0.007% (n=23).

In intervention sites, sterilisation coverage in juveniles rose after sterilisations, then declined steeply within the next 6 months. Coverage fluctuated due to few sightings of juveniles, particularly in sites 4N and 5N, therefore no statistical analysis was performed (Fig. S11).

## 2.2 Count survey results: sex structure (Models 12 & 13)

Males consistently dominated adult populations but there was a 2% increase in the number of adult females per total adult count (i.e. proportion of females) over the study period from 0.36 (CI = 0.33-0.39) to 0.38 (CI = 0.35-0.41) in control sites and from 0.39 (CI = 0.36-0.42) to 0.41 (CI = 0.38-0.44) in intervention sites, based on predicted values from models. The intervention sites were not significantly different to control sites ($IRR_{intervention}$ = 0.99, CI = 0.97-1.02). In contrast, sex proportions were comparably even in juveniles with an increase in the number of juvenile females per total juveniles over the study period from 0.53 (CI = 0.49-0.58) to 0.61 (CI = 0.56-0.66) in control sites and from 0.52 (CI = 0.48-0.56) to 0.55 (CI = 0.5-0.6) in intervention sites, again, not significantly different between intervention and control sites ($IRR_{intervention}$ = 0.96, CI = 0.88-1.05; Table S8).

## 2.3 Count survey results: seasonality (Models 2-5, 10 & 11)

Adults were increasingly likely to be seen from June to a peak in December and January, then decreasingly likely to be seen until May and June (Fig. S7). Adults were less likely to be seen in the afternoon (IRR = 0.74, CI = 0.68-0.8, Table S5), but this effect was most pronounced in hotter months between October and May (outside the monsoon season; Fig. S7). Juveniles were more likely to be seen between November to April and puppies were most likely to be seen in December and January and least likely in May and June (Table S5, Fig. S7). Puppies and juveniles were also less likely to be seen in the afternoon ($IRR_{puppy}$ = 0.84, CI = 0.78-0.90, $IRR_{juvenile}$ = 0.76, CI = 0.72-0.81, Table S5). Adults and juveniles were more likely to be seen during surveys in the monsoon period when it wasn’t raining ($IRR_{adult}$ = 0.92, CI = 0.86-0.99, $IRR_{juvenile}$ = 0.69, CI = 0.55-0.86), but less likely to be seen if it was the monsoon period and it was raining during the survey ($IRR_{adult}$ = 1.10, CI = 1.04-1.16, $IRR_{juvenile}$ =1.71, CI = 1.44-2.02, Table S5).

Underweight dogs were more likely to be seen in May to November, with a peak just after the monsoon had finished (Fig. S13). Dogs with skin problems were more likely to be observed February to April, with a second rise in November (Fig. S13).

## 2.4 Count survey results: dog health indicators

Surveyors reported that on most observations adults and juveniles had no skin problems (86.49%, 95% CI 86.35–86.64, n = 180,501), 9.9% of observations reported a mild skin condition (95% CI 9.77–10.03, n = 20,656), 2.53% reported a moderate skin condition (95% CI 2.46–2.6, n = 5275) and only 1.08% reported a severe skin condition (95% CI 1.04–1.13, n = 2257). There were no differences in skin conditions between sterilised and unsterilised dogs and lactating and non-lactating females. Similar patterns of dog health were directly observed by veterinarians at sterilisation where 80.8% had a body condition of ideal and 88.6% had no skin problems (n = 2,899 entire dogs).

There were more underweight adults sighted between May and September (Table S7, Fig. S13). More underweight dogs were seen in sites with higher dog density (IRR = 1.15, CI = 1.06 - 1.26; Table S7). Lower densities were associated with predictions of 5.76% underweight adults (CI = 4.58–7.24%) and higher densities were associated with 9.68% underweight adults (CI = 7.53–12.44%) at the start of sterilisations.

Model 11 predicted the estimated proportion of juveniles and adults with skin problems stayed consistent starting at 10.69% (CI = 8.44–13.54) and ending at 11.04% (CI = 8.86–13.76%) in control sites and from 12.06% (CI = 9.4–15.49%) to 11.06% (CI = 8.78–13.94%) in intervention sites over the study period (Table S7, Fig. S16). This was consistent in control and intervention sites ($IRR_{control}$ = 1.02, CI = 0.93-1.11, $IRR_{intervention}$ = 0.93, CI = 0.83-1.06).

There was some non-linear variation detected in underweight and dogs with poor skin condition over time n GAMM analyses, but not on an annual cycle, suggesting this was more likely to be a long-term trend rather than a seasonal pattern (Fig. S16). Whilst the GLMM for underweight dogs did not detect a difference between the intervention and control groups, the GAMM comparison suggested that the intervention was significant in reducing dog counts. The pattern seems to show that underweight dogs in the intervention group declined faster than those in the control group, but that they were similar by the end of the study, as reflected in the GLMM results that did not show a significant difference over the entire study period.

## 2.5 Community perceptions questionnaire results

Of the 3056 in-person community surveys, 20% (n = 618) of participants shared their phone number for follow-up surveys and full survey responses were obtained for 54% of these (n = 334). Participants were not evenly distributed across sites and were grouped into intervention (n = 132) and control sites (n = 202) for analysis. The demographics of participants that completed both surveys were similar to

those that completed only the first survey in terms of age, religion, and whether they fed FRDs, but were more likely to be male (61.3% vs 42%, $p < 0.01$) and care for a dog (41.9% vs 18.4%, $p < 0.01$).

*Opinions on FRDs (Table S12, S14 & Fig. S15)*

Participants who were not sure if there was sterilisation ongoing in the area reported dogs being a problem less often (OR = 0.56, CI = 0.36–0.87, Table S14).

Overall, most people reported being rarely or never scared of FRDs (proportion = 0.63, CI 0.59–0.66), Table S12). In Q2, participants from intervention sites were more likely to say they were frequently or always scared of FRDs (OR = 2.29, CI = 1.15–4.55, Table S14) and control sites were less likely to report this (OR = 0.63, CI = 0.4–0.99, Fig. S15). Participants in control sites were more likely to report that they frequently or always liked FRDs in Q2 compared to Q1 (OR = 4.8 , CI = 3.15 - 7.3, Fig. S15), whereas responses of participants in intervention sites were consistent between surveys (OR = 0.26 , CI = 0.13–0.5, Fig. S15), with most participants rarely or never liking FRDs (Prop survey 1 = 0.45, survey 2 = 0.39, Table S14). Participants in the control sites were more likely to report playing with FRDs more often in Q2 compared to Q1 (OR = 2.11, CI = 1.34–3.31) but there was little change in responses of participants in the intervention sites in survey 2 (OR = 0.45, CI = 0.22–0.91). Most participants reported that FRDs were aggressive to each other sometimes (0.52, CI 0.48–0.56). The model for this question was not better than the null model, therefore we conclude that there was not a significant difference between surveys or between intervention and control groups. Most participants thought FRDs were rarely or never aggressive to pet dogs (0.73, CI 0.69–0.76), but this opinion did not differ between surveys or intervention and control sites (Fig. S15). Participants caring for dogs and those who reported being aware of sterilisation campaigns were more likely to frequently or always like FRDs ($OR_{dog\ owner}$ = 1.89, CI = 1.35–2.65, $OR_{aware}$ = 1.89, CI = 1.04–3.43) and frequently or always pet or play with FRDs ($OR_{dog\ owner}$ = 1.88, CI = 1.34–2.65, $OR_{aware}$ = 1.89, CI = 1.07–3.39). Most participants reported that they rarely or never petted or played with FRDs (proportion = 0.65, CI 0.61–0.69, Table S14).

*Changes in FRD numbers (Table S13, S15 & Fig. S16)*

Participants with dogs in their household were more likely to report more puppies (OR = 1.68, CI = 1.11–2.57, Table S15). There was no change in participants' reports of overweight dogs in the control sites (OR = 0.82, CI = 0.47–1.42, Table S15) but fewer people in intervention sites said there were fewer overweight dogs, and more people said there were the same or more overweight dogs since the previous year (OR = 9.85, CI = 2.09–46.31, Fig. S16). Participants caring for a dog were more likely to report fewer overweight FRDs since the previous year (OR = 0.54, CI = 0.31–0.93, Table S15). In the second survey, fewer participants said there were fewer underweight dogs and more participants said

there were more underweight dogs in intervention and control sites ($OR_{control}$ = 2.86, CI = 1.63–5.03, $OR_{intervention}$ = 1, CI = 0.35–2.86, Fig. S16). Participants tended to report FRDs with no wounds (Control = 52.27%, n = 207, Intervention = 39.57%, n = 91, Table S13). Since this response was not included in the ordinal models, there was a low sample size for the model describing changes in FRDs with wounds (Control n = 68, Intervention n = 76, Table S15) and the model was only marginally better than the null model based on the AIC (difference of -2), therefore we conclude that there were no significant changes to responses in the second survey or between intervention and control groups.

*Differences between count surveys and community perceptions*

Perceptions of FRD body condition from the community did not consistently align with results from count surveys. In all sites, residents reported more underweight dogs in the second survey, contrary to the decline seen on surveys. However, in intervention sites, other participants reported more overweight dogs, possibly suggesting that the improvement in condition documented on surveys was detectable by residents in intervention sites. Community surveys did not include visual definitions of 'underweight' or 'overweight' for dogs, thus estimation of trends in body condition by untrained individuals are likely to be less reliable than trained surveyors that classify each dog. Community survey participants reported the decrease in puppies in intervention sites that surveys demonstrated, but an overall decrease in dogs in intervention sites only, whereas surveys suggested that this occurred in all sites. Residents' opinions are likely to be subject to more bias than regular, consistent surveys performed by trained individuals, however we can't rule out that citizens may be detecting changes in dog ecology at different times or spaces than our surveyors recorded.

## 2.6 Cared-for dog questionnaire results

Of the cared for dogs, 84.9% were adults, 8.4% juveniles and 5.7% were puppies under 3 months old. Male dogs were more common than females (72.3%). Of these cared-for dogs, 42.4% were allowed to roam outside the property of their human guardian, 54.9% roamed during the day and night, 13.4% roamed for some or all the day and 16.9% roamed in the evening or night. Of those that were roaming at any time (across all sites), 31.8% were sterilised. Dogs adopted from the street are mostly allowed to roam by their new carers and are almost all local breed 'Indy' type dogs, rather than pedigree breeds. Indy dogs are adopted locally, 68% of Indy dogs come from within 1km, whereas pedigree dogs are more likely to come from within the state of Goa (58% of pedigree dogs).

## 3. Supplementary Figures

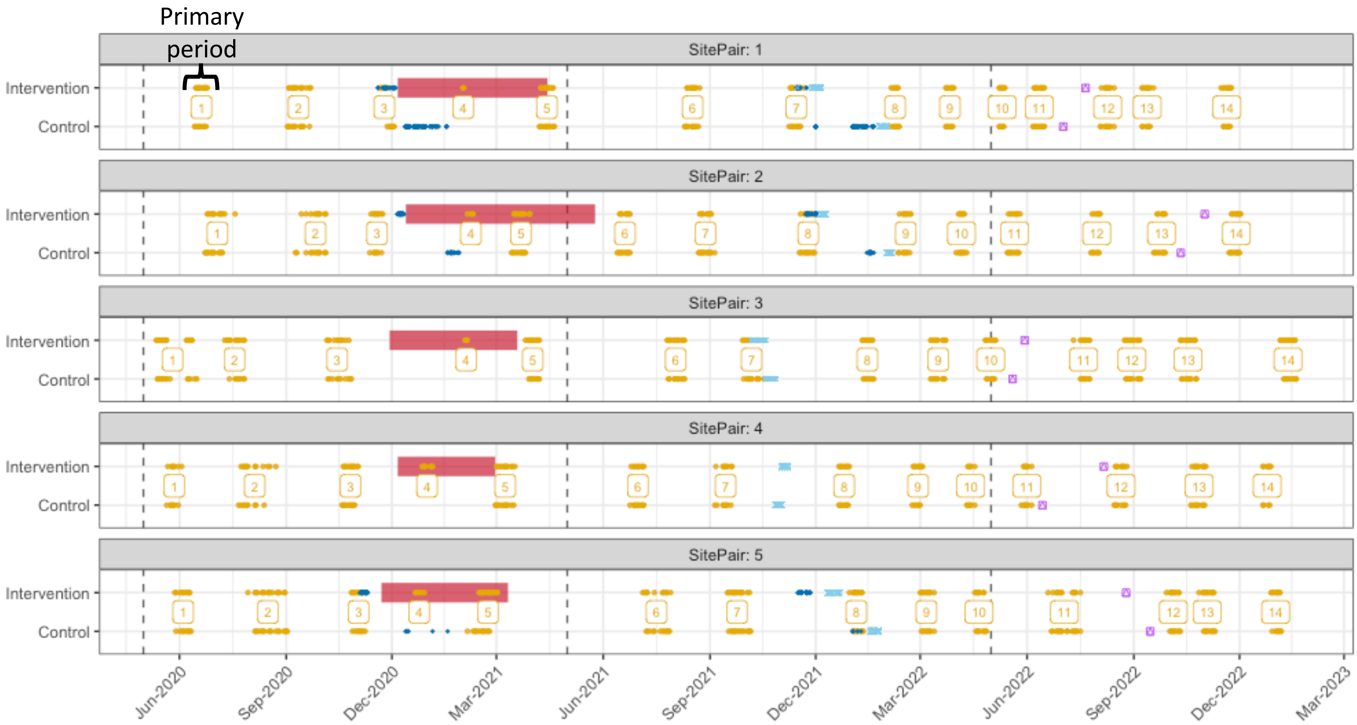


Figure S1. Timeline of study surveys from May 2020 to January 2023. Orange points indicate free-roaming dog (FRD) count secondary surveys and numbers indicate the primary sampling periods. Dark blue indicates community questionnaires and light blue indicates cared-for dog questionnaires. Red blocks indicate the surgical sterilisation intervention period. Mark resight surveys of FRDs are indicated with purple squares. COVID-19 restrictions prevented community questionnaires in site pairs 3 and 4.

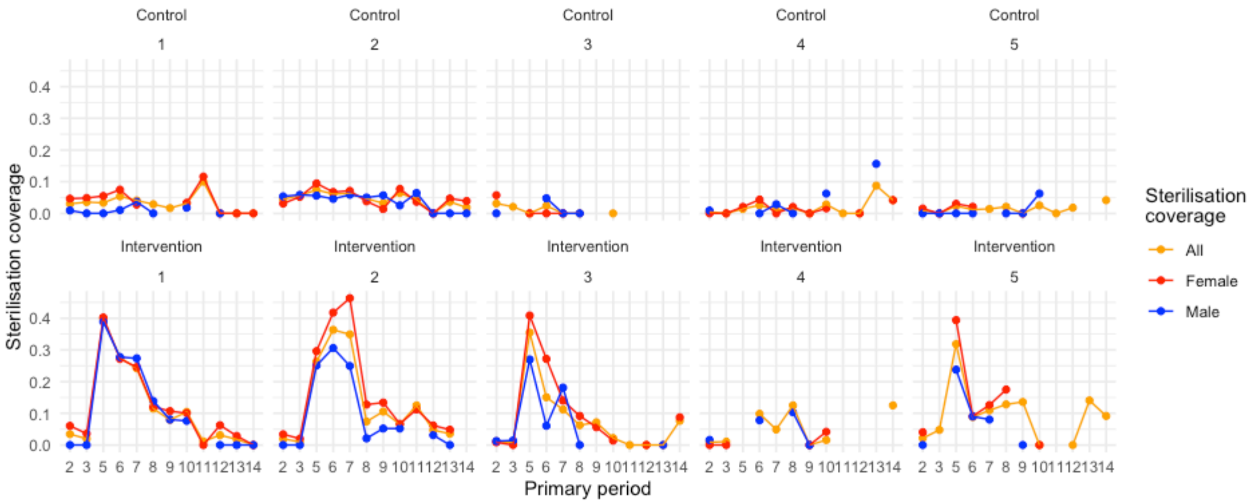


Figure S2. Observed proportions of sterilised juvenile free-roaming dogs for each primary period (survey round) of the study. Points are missing where no juveniles were observed, fluctuations due to low numbers of juveniles sighted.

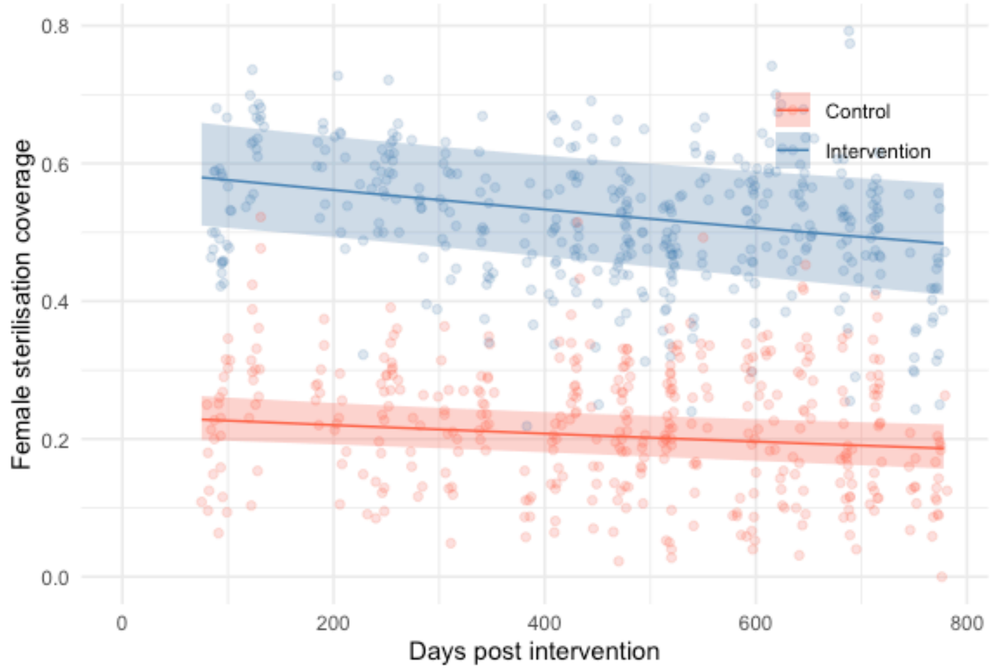


Figure S3. Female sterilisation coverage: the fraction of adult female free-roaming dogs (FRDs) sterilised of all female adult FRDs. Estimated mean proportion (lines) from Model 1 with 95% confidence intervals (shaded areas) for intervention (blue) and control sites (red) over time. Points show observed proportions for each site survey with corresponding colours. Model predictions are based on an average of all random effects.

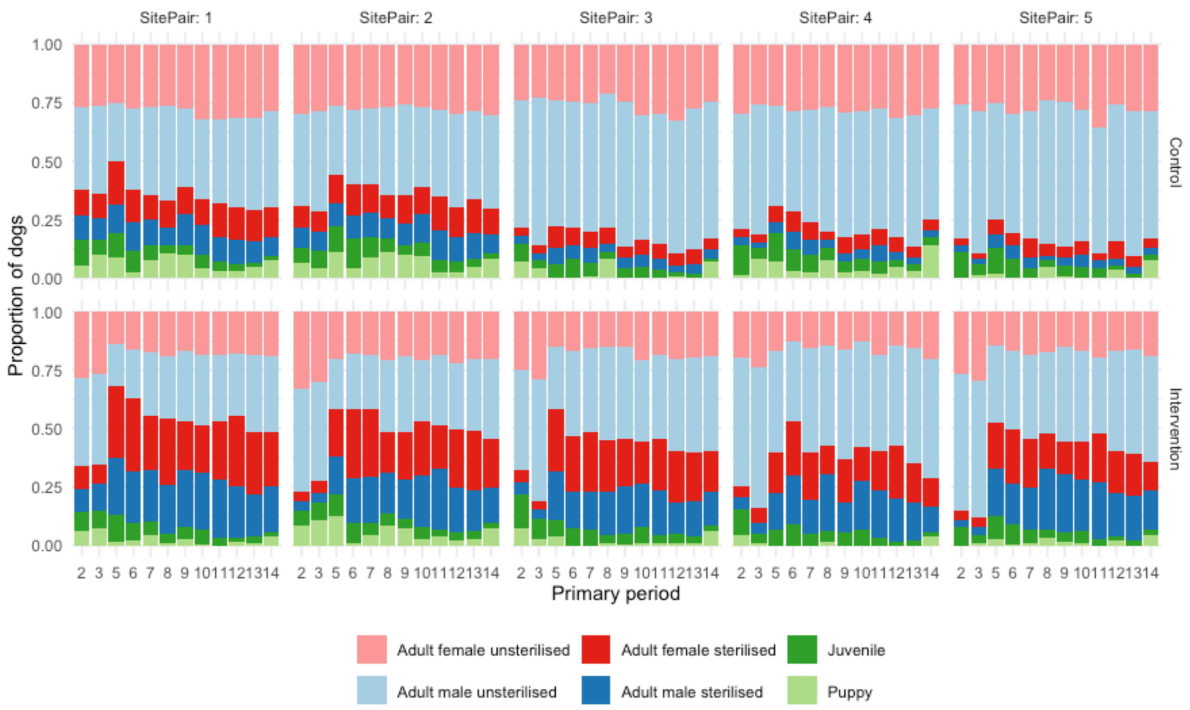


Figure S4. Relative composition of observed free-roaming dog population in all site pairs showing mean fractions of adults by sex (male – blue, female – red) and sterilisation status (sterilised = darker shade, unsterilised = lighter shade) and juveniles (dark green) and puppies (light green) per primary period. The plot shows most of the population is adult dogs, with juveniles and puppies contributing little to population size. An increase in sterilised dogs (dark blue and red) is evident in intervention sites post-intervention (after primary period 3).

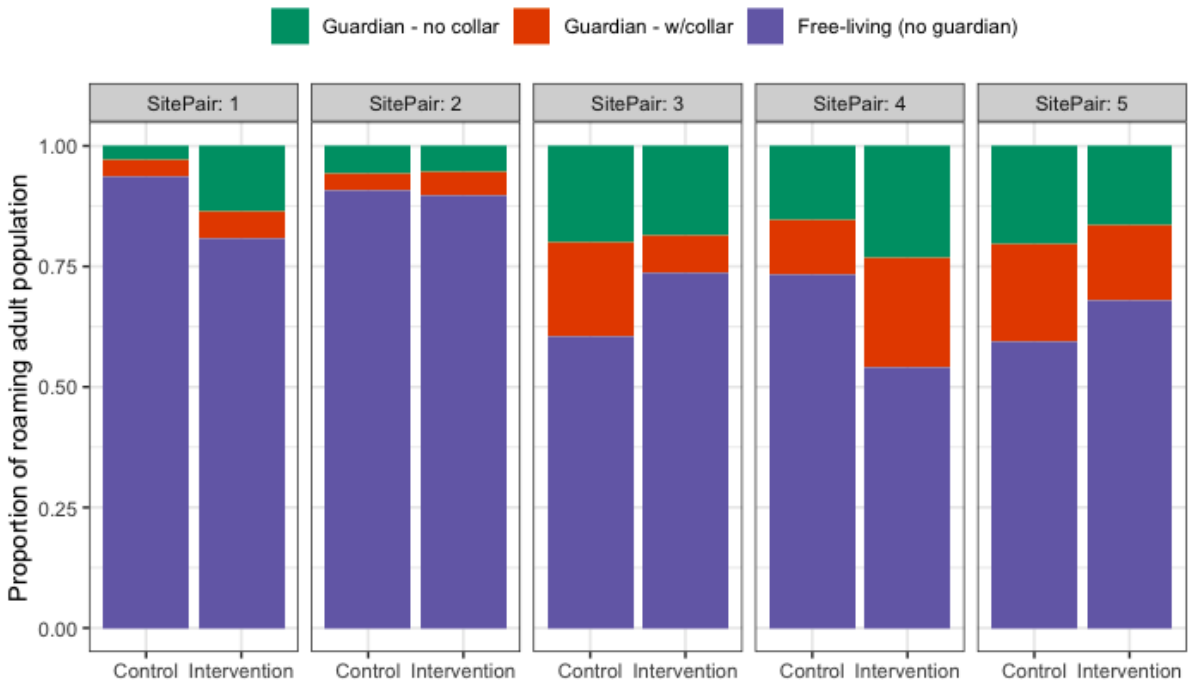


Figure S5. Estimated proportion of dogs with guardians in the free-roaming population. On dog count surveys, we assumed that FRDs with a collar had a guardian/were cared-for, but that a proportion of dogs without a collar may also have a guardian and estimated this proportion from household questionnaires. Mean proportions are shown for entire study period as there was minimal change over time. Intervention sites had significantly higher proportions of dogs with guardians in pairs 1 and 4 and vice versa in pair 3, all other pairs were not significantly different to each other. Sites with higher proportions of dogs with guardians might be expected to have healthier, more fecund females and more puppies abandoned into the FRD population however, there was no evidence in counts to suggest this was the case.

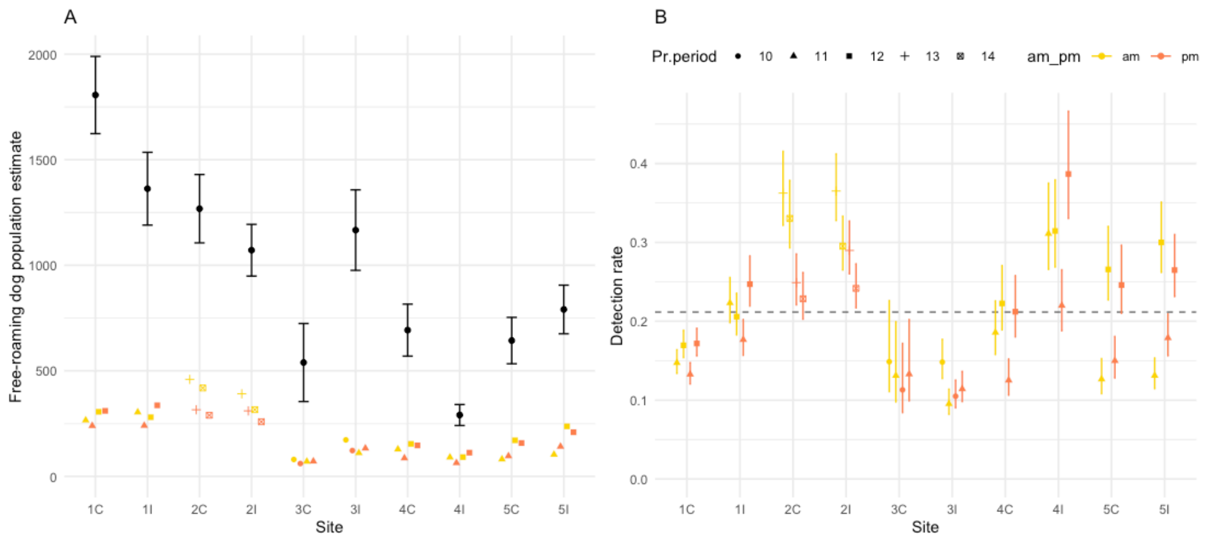


Figure S6. Free-roaming dog (FRD) population estimates and FRD count survey detection rates. A) Population estimates (Chapman method) from mark-resight surveys (points with bars showing 95% confidence intervals) compared to FRD survey counts of FRDs (crosses). B) detection rates calculated from nearest primary sampling period (Pr. period) to the mark-resight survey. Mean detection rate indicated by grey dashed line (0.21). Morning surveys are in yellow and afternoon surveys are in orange.

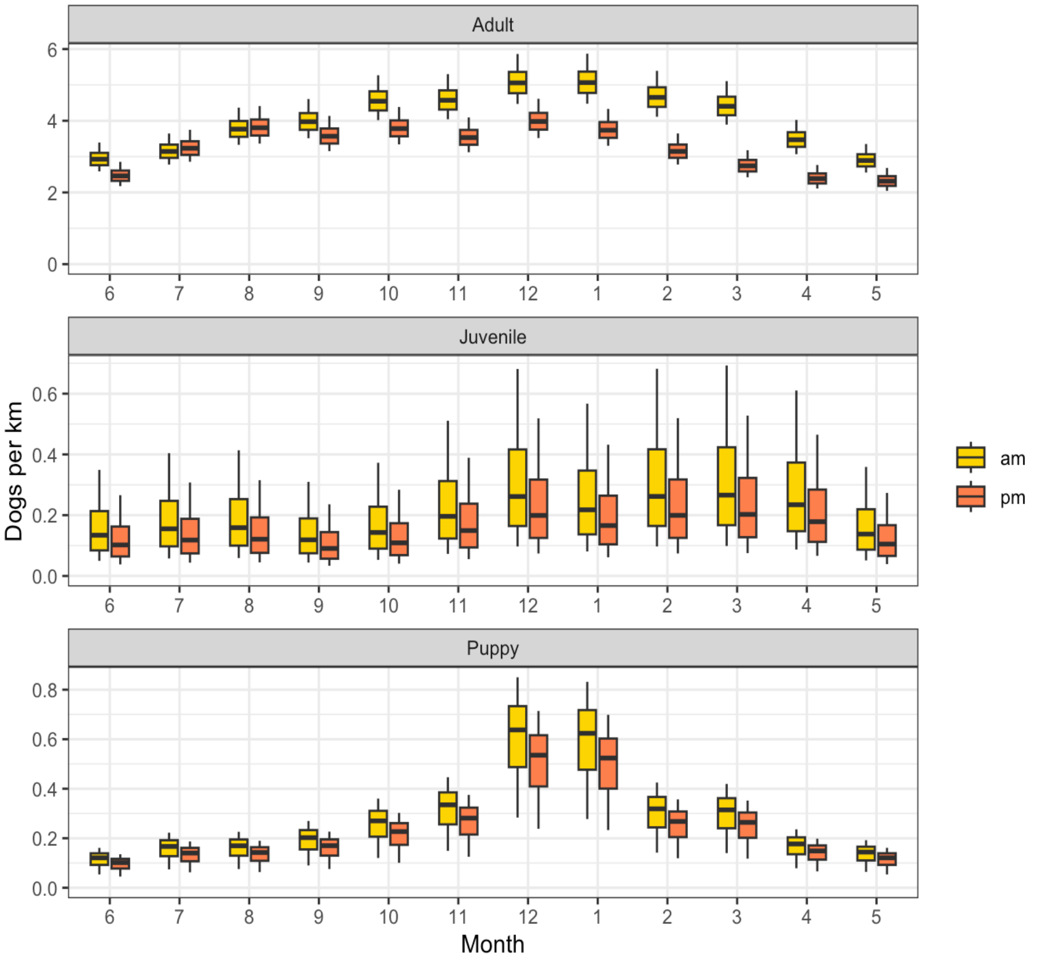


Figure S7. Seasonal patterns in estimated counts of adult (top), juvenile (middle) and puppy (bottom) free-roaming dogs per km by month and time of survey (am – yellow, pm - orange) across all study sites from generalised linear mixed models 1, 2, and 3 (Table S3).

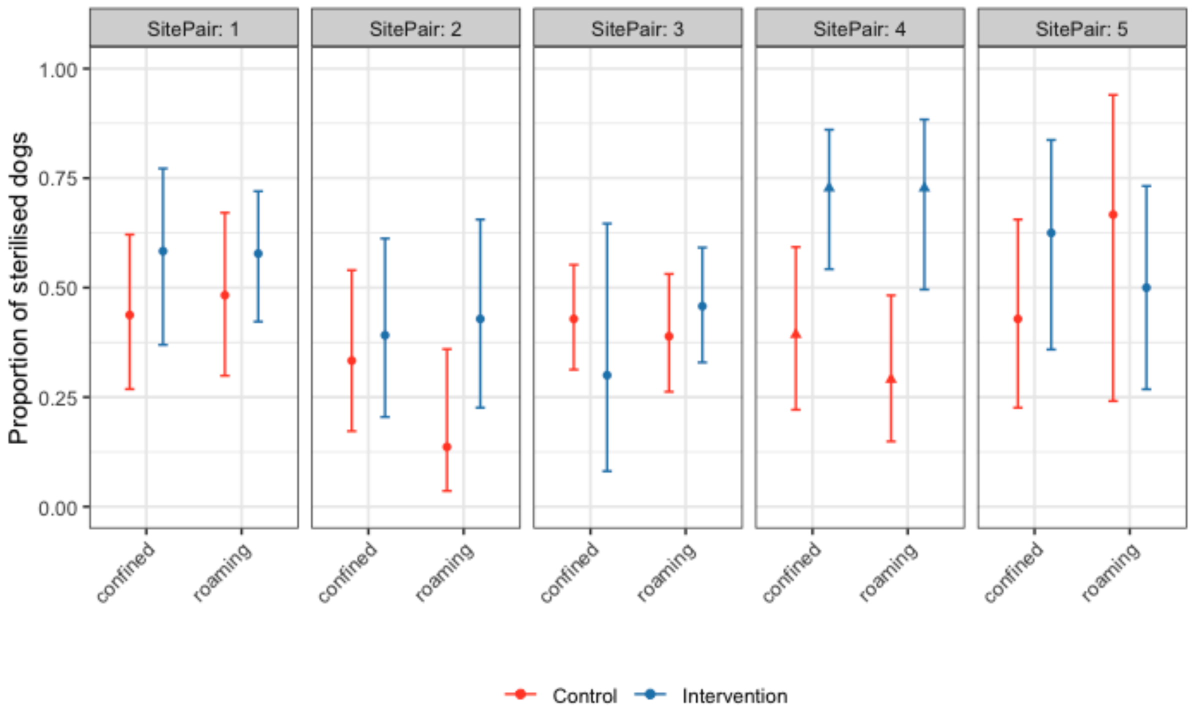


Figure S8. Female sterilisation coverage in confined and roaming populations of cared-for dogs based on questionnaire data after interventions. Triangles denote a statistically significant difference between proportions in intervention and control sites judged on a p-value of < 0.05 (using 2-sample test of equal proportions in base R).

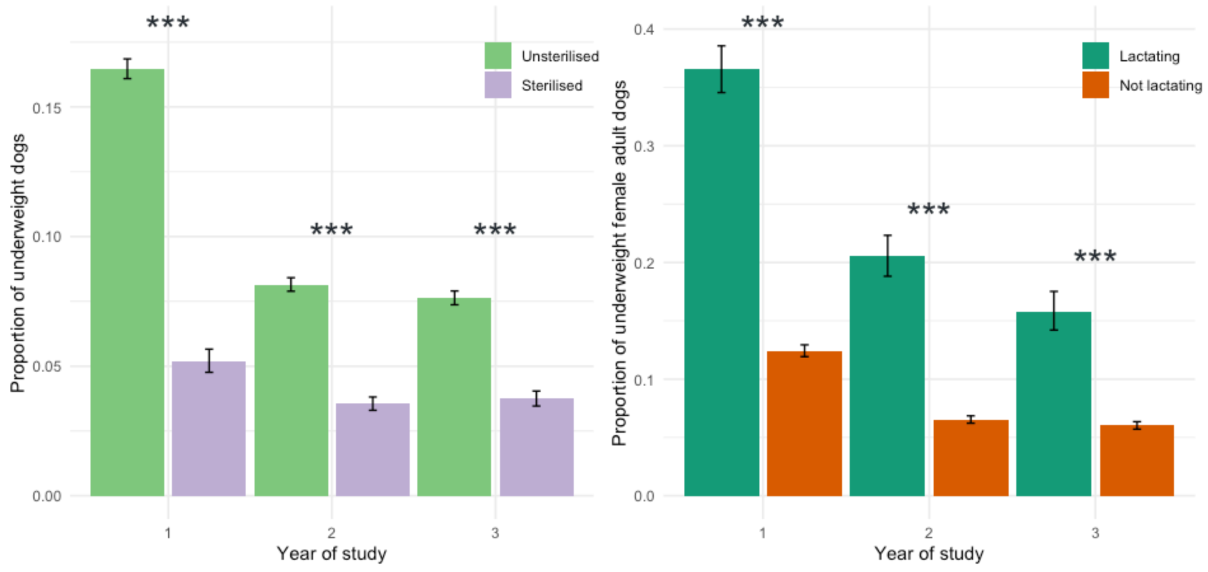


Figure S9. Proportions of underweight and emaciated (body condition score 1 or 2) observations comparing entire and sterilised adult free-roaming dogs of both sexes (left) and female lactating and non-lactating (right) for each year of the study starting in May 2020 (year 1), May 2021 (year 2) and May 2022 to January 2023 when the study finished (year 3). *** denotes a statistically significant difference between proportions with p-value < 0.001.

A

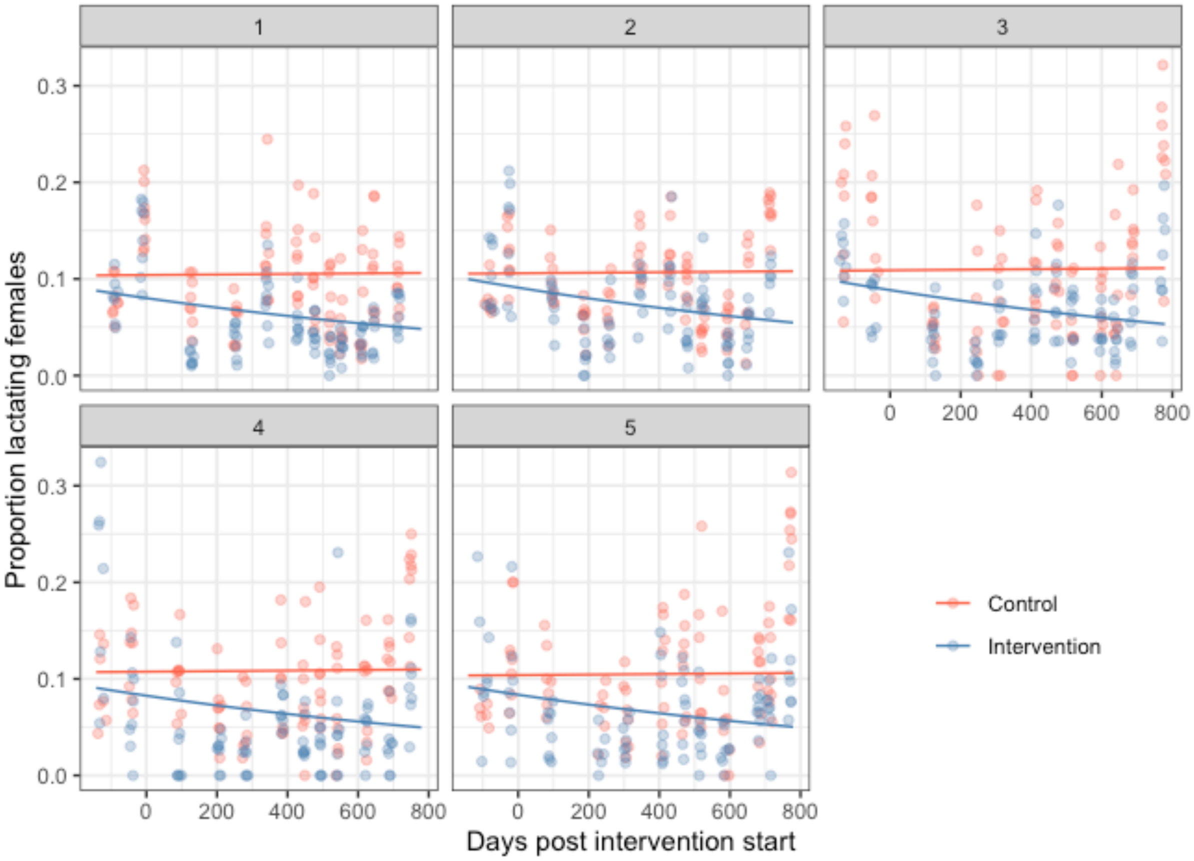

1
2
3
4
5
0.3
0.2
0.1
0.0
0
200
400
600
800
Proportion lactating females
Days post intervention start
Control
Intervention

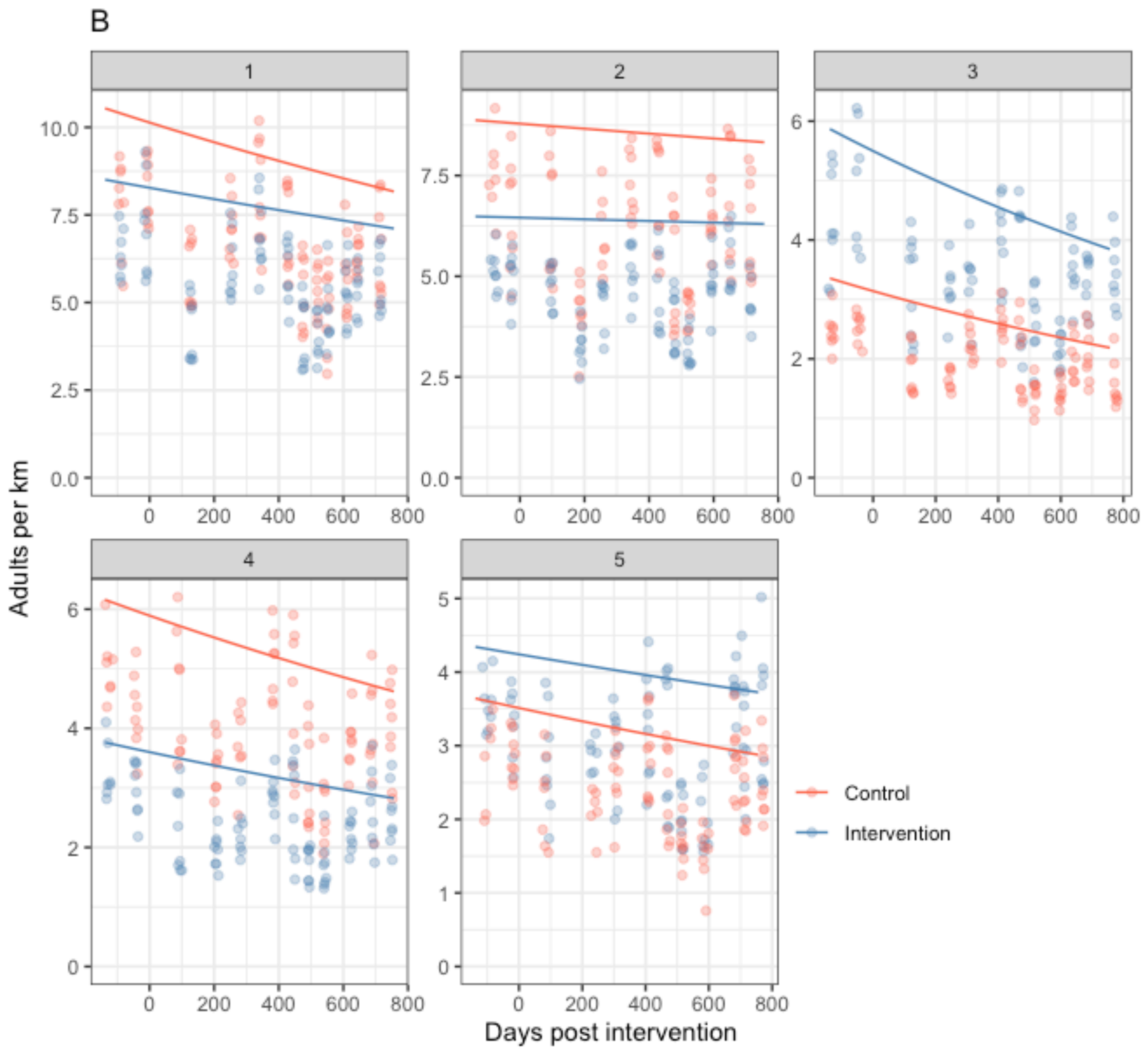
B
1
2
3
4
5
Adults per km
Days post intervention
Control
Intervention

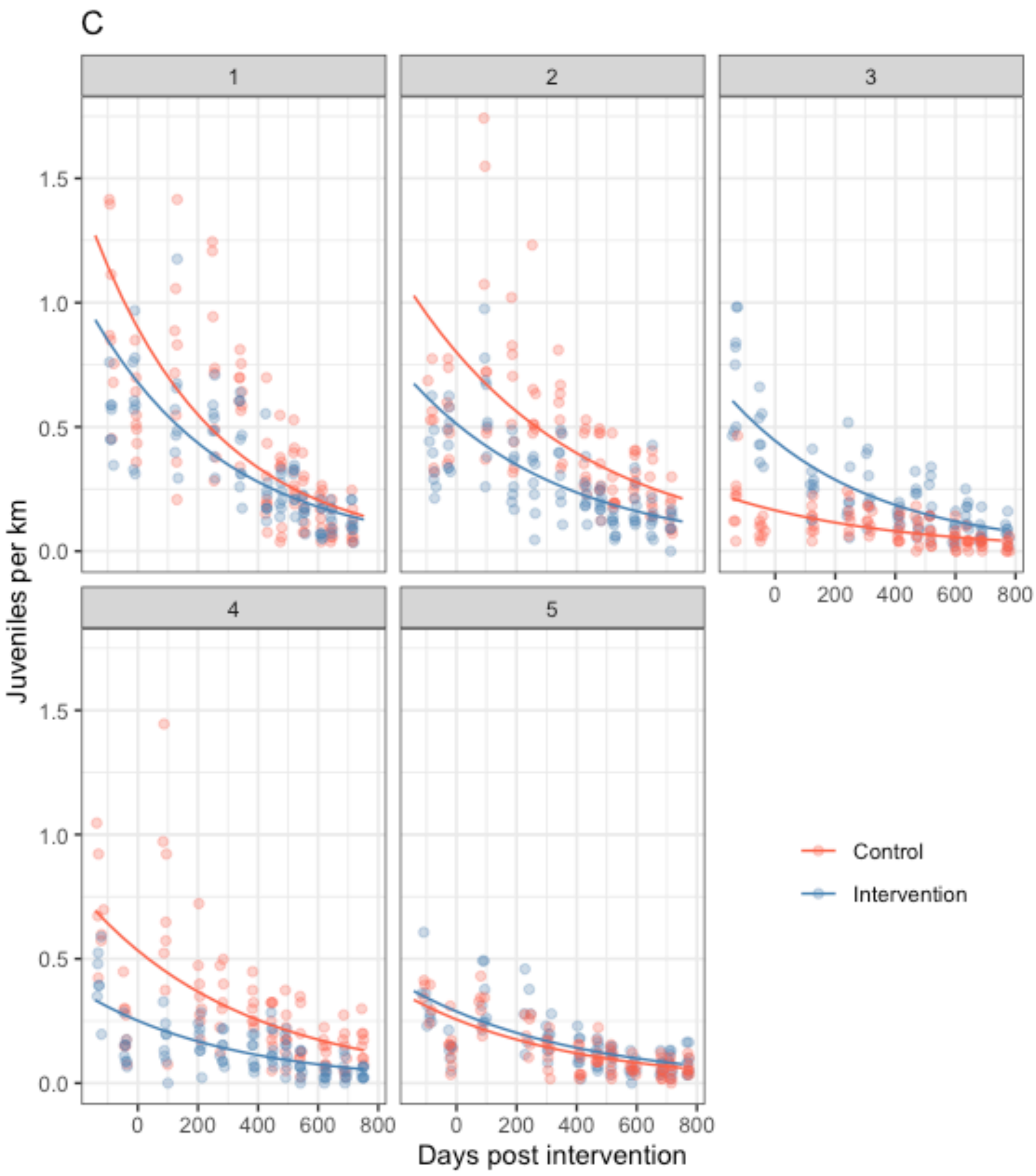

C
1
2
3
4
5
Juveniles per km
0.0
0.5
1.0
1.5
0
200
400
600
800
Days post intervention
Control
Intervention

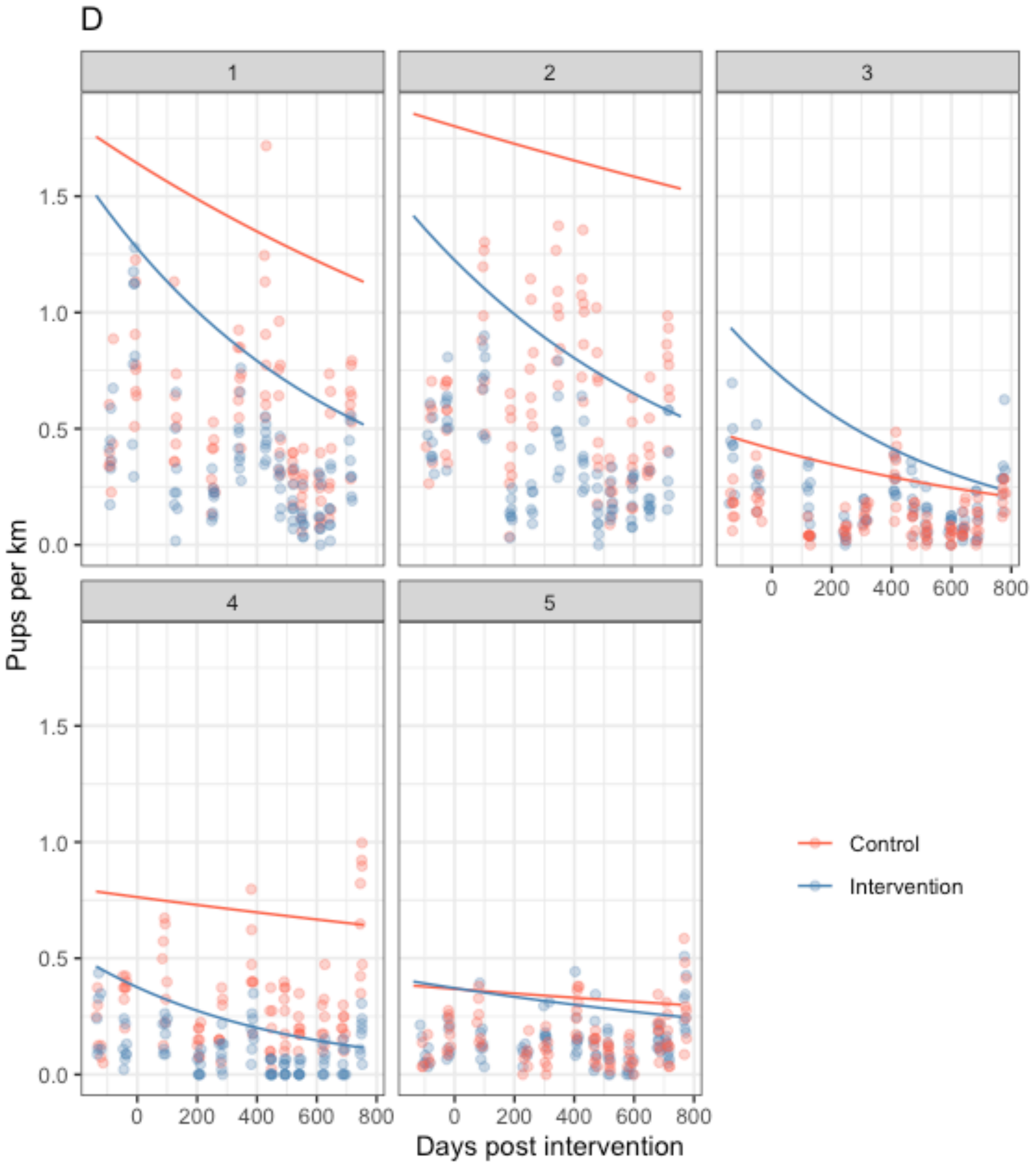

D
1
2
3
4
5
Pups per km
0.0
0.5
1.0
1.5
0
200
400
600
800
Days post intervention
Control
Intervention

E

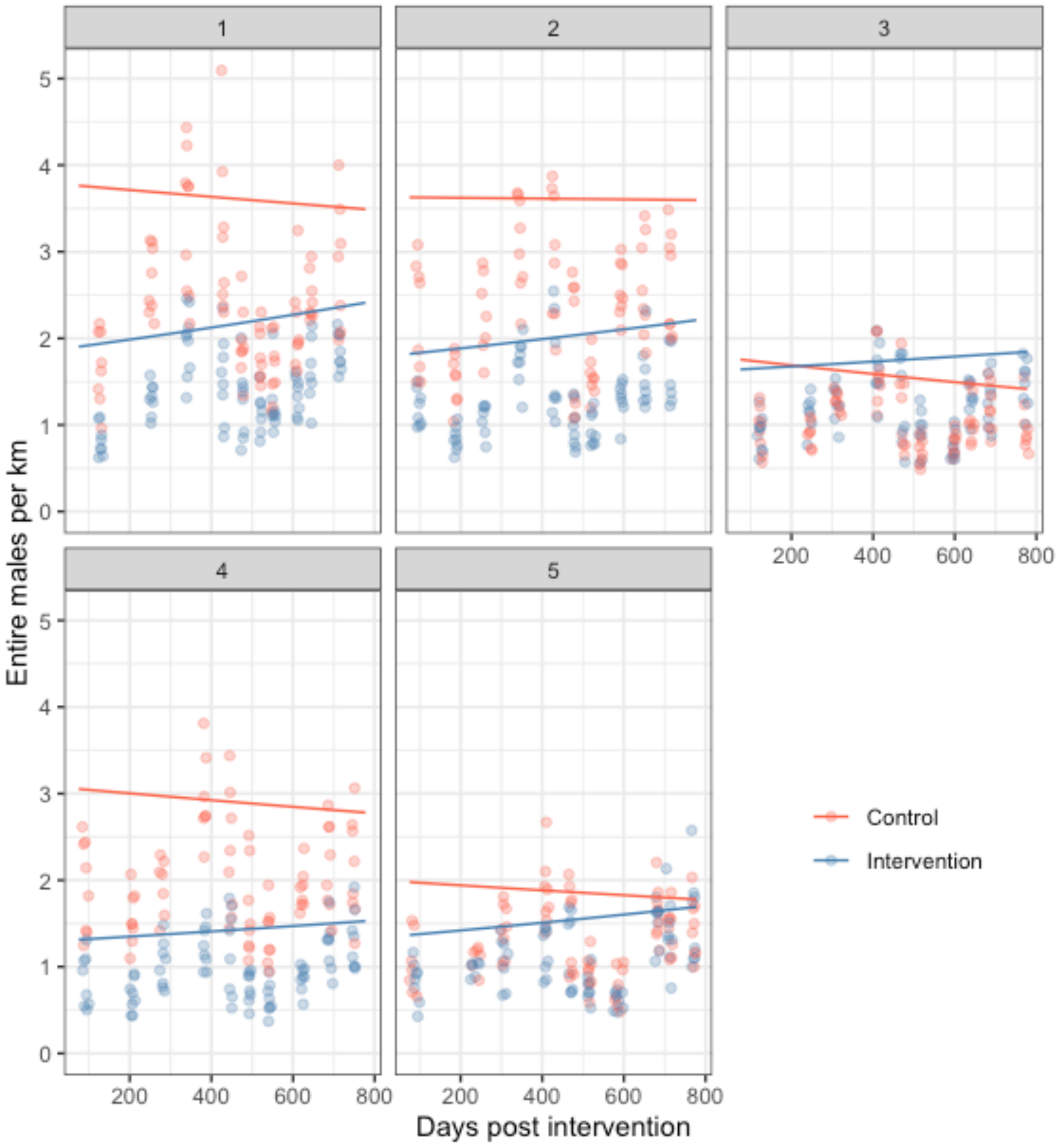

1
2
3
4
5
Entire males per km
Days post intervention
0
1
2
3
4
5
200
400
600
800
Control
Intervention

F

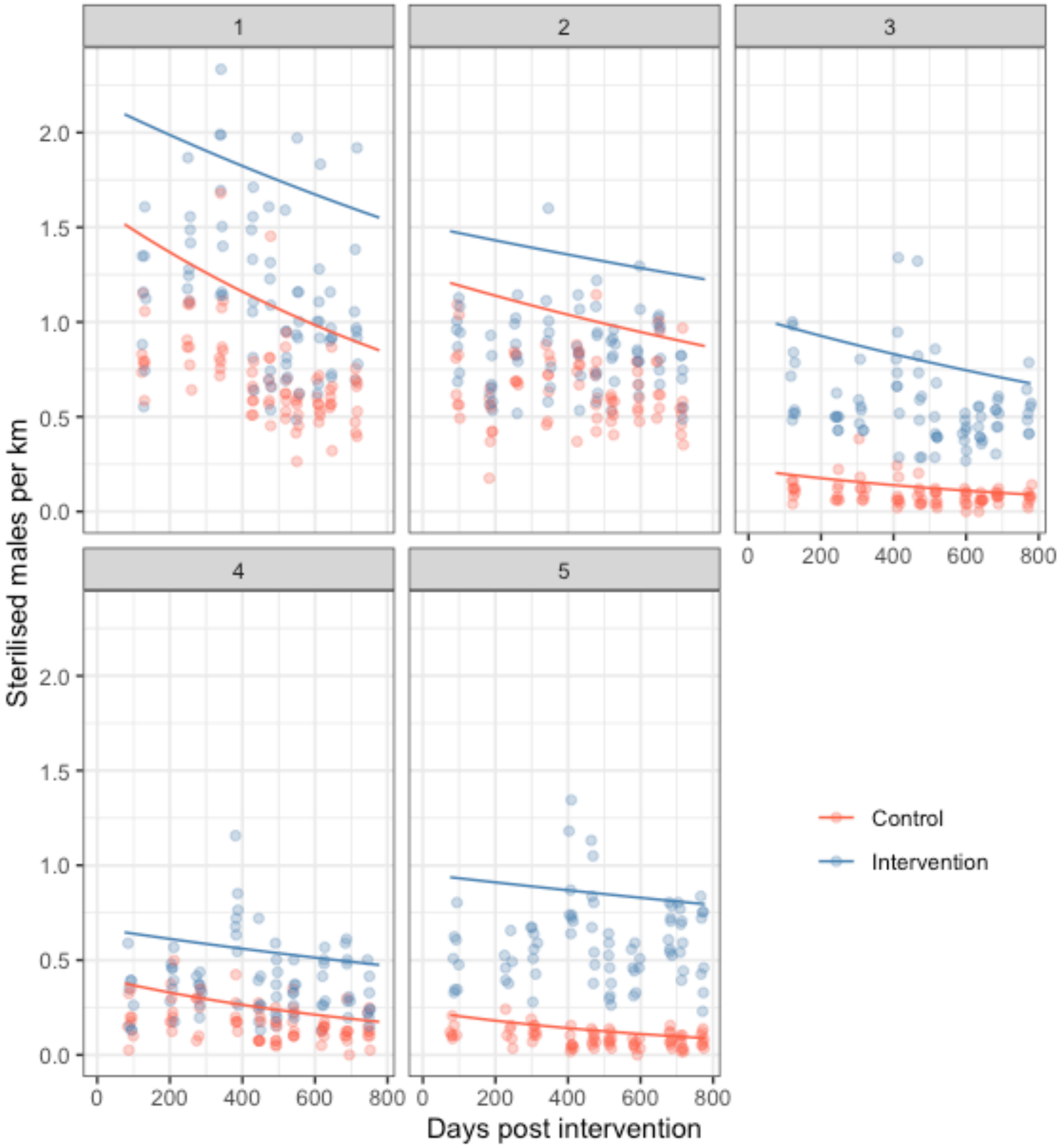

1
2
3
4
5
2.0
1.5
1.0
0.5
0.0
0
200
400
600
800
Sterilised males per km
Days post intervention
Control
Intervention

G

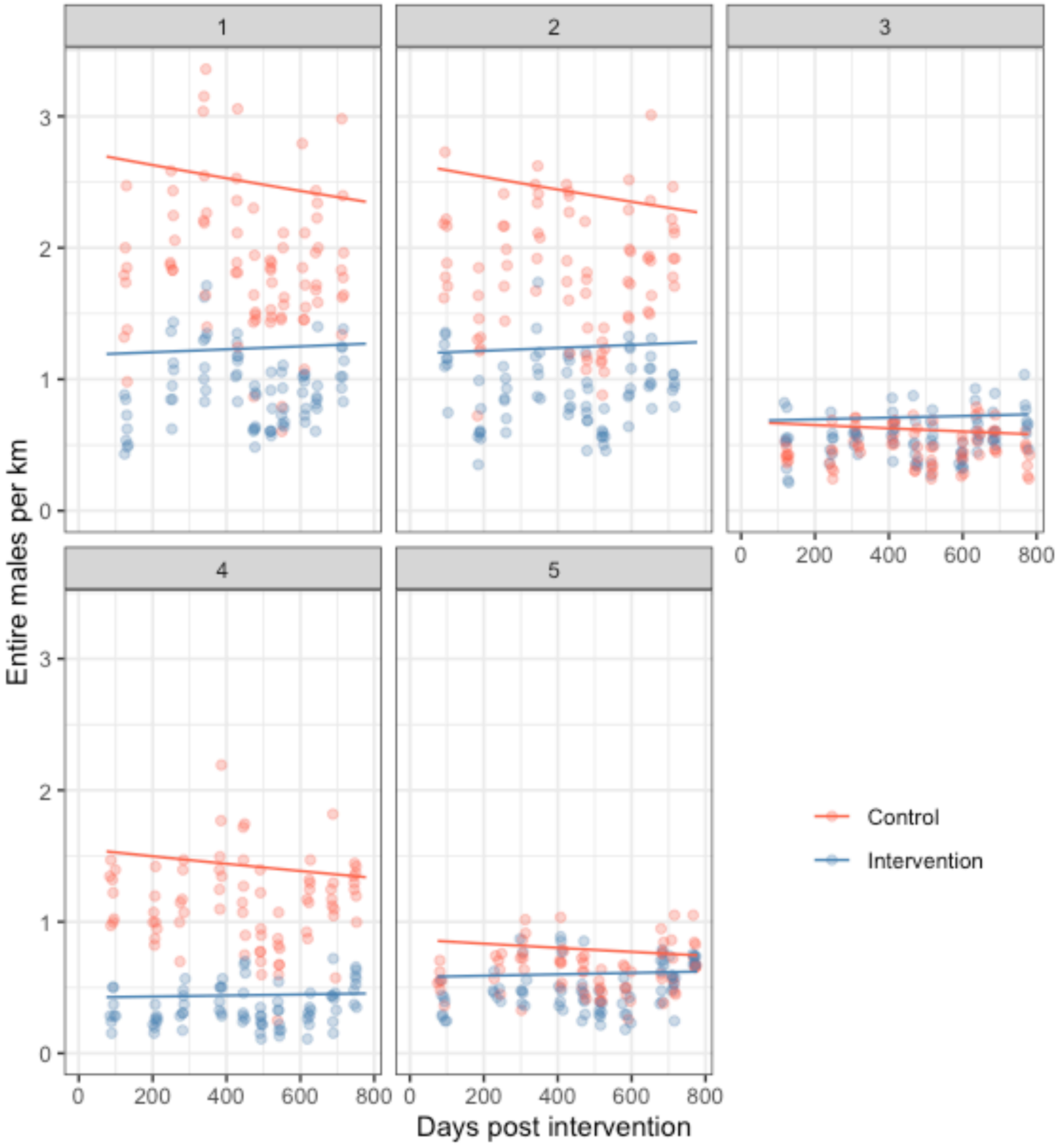

1
2
3
4
5
Entire males per km
Days post intervention
0
1
2
3
0
200
400
600
800
Control
Intervention

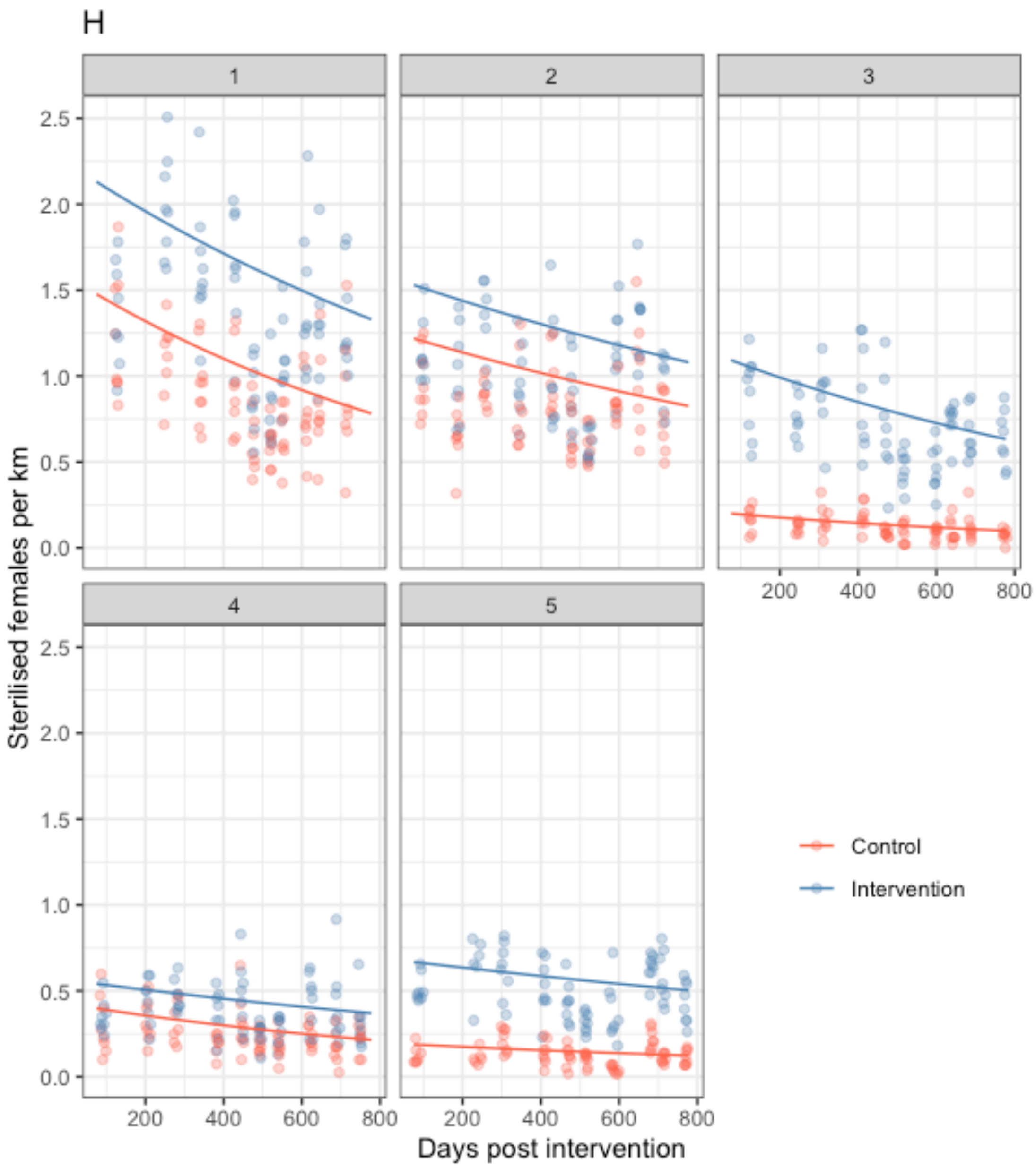


Figure S10. Estimated mean free-roaming dog counts per km and proportions for each site from generalised linear mixed models for intervention (blue line) and control sites (red line) over time post-intervention. A - proportion of lactating females (model 5), B – adult (model 2), C – juvenile (model 3), D – puppy (model 4), E - entire adult male (model 6), F = sterilised adult male (model 7), G - entire adult female (model 8) and H – sterilised adult female (model 9) counts per km of road surveyed. Points show the observed counts for each site survey. Predictions are for morning surveys, without rain or monsoon and in January (where those variables are present in the models, see Table S3 for list of variables).

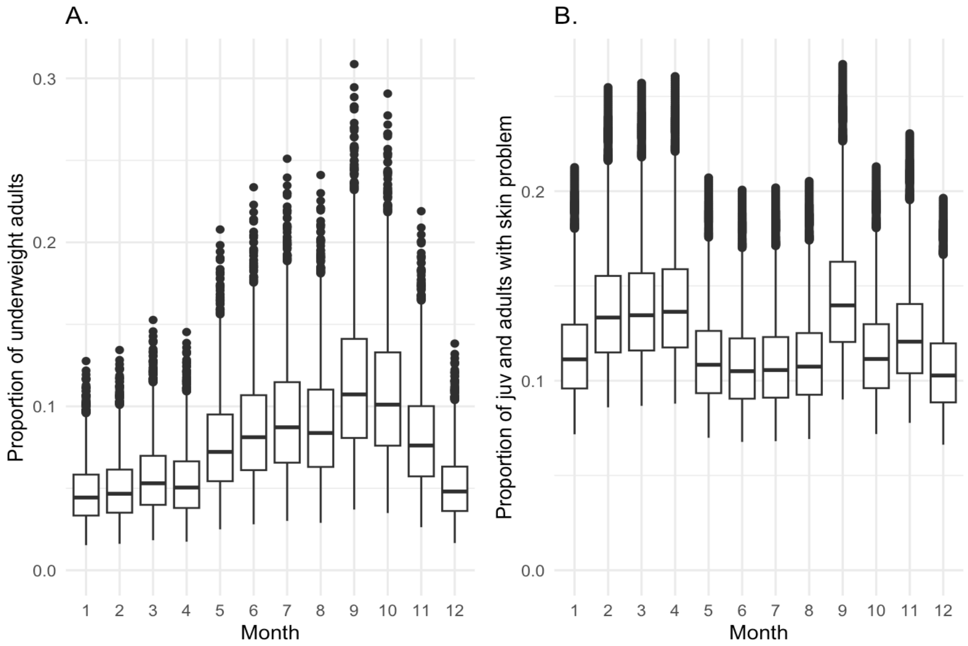


Figure S11. Estimated mean proportions of A) underweight free-roaming dogs and B) free-roaming dogs with a skin problem by month of the year for all sites based on predictions from Models 10 & 11.

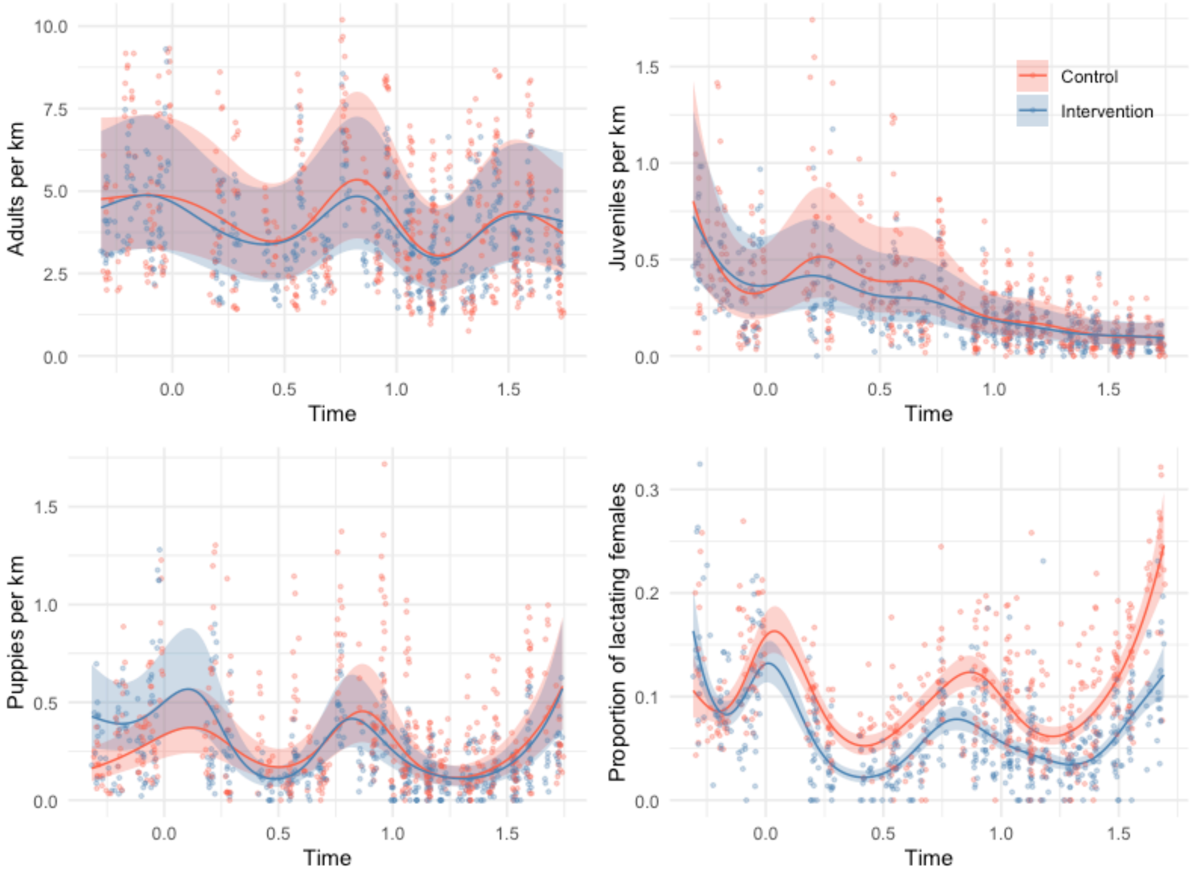


Figure S12. Estimated mean adult, juvenile and puppy counts per km and the proportion of lactating females, from general additive mixed models with 95% confidence intervals for intervention (blue line) and control sites (blue line) over time (0 indicates intervention start point). Points show the observed counts for each site survey. Predictions are based on an average of all random effects for morning surveys, without rain or monsoon and in January (where those variables are present in the models).

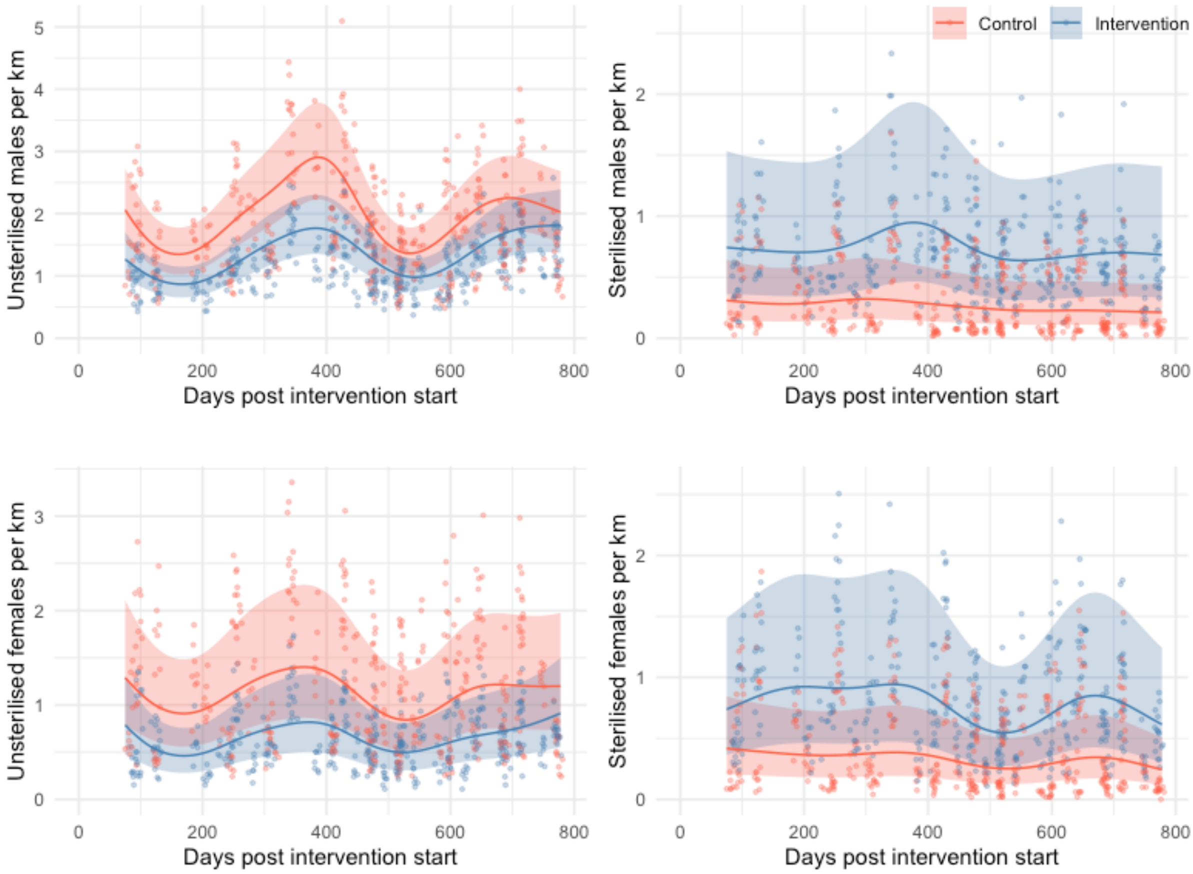


Figure S13. Estimated mean counts per km of adult entire male, sterilised male, entire female and sterilised female from general additive mixed models with 95% confidence intervals for intervention (blue line) and control sites (red line) over time post-intervention. Points show the observed counts for each site survey. Predictions are based on an average of all random effects for morning surveys, without rain or monsoon and in January (where those variables are present in the models).

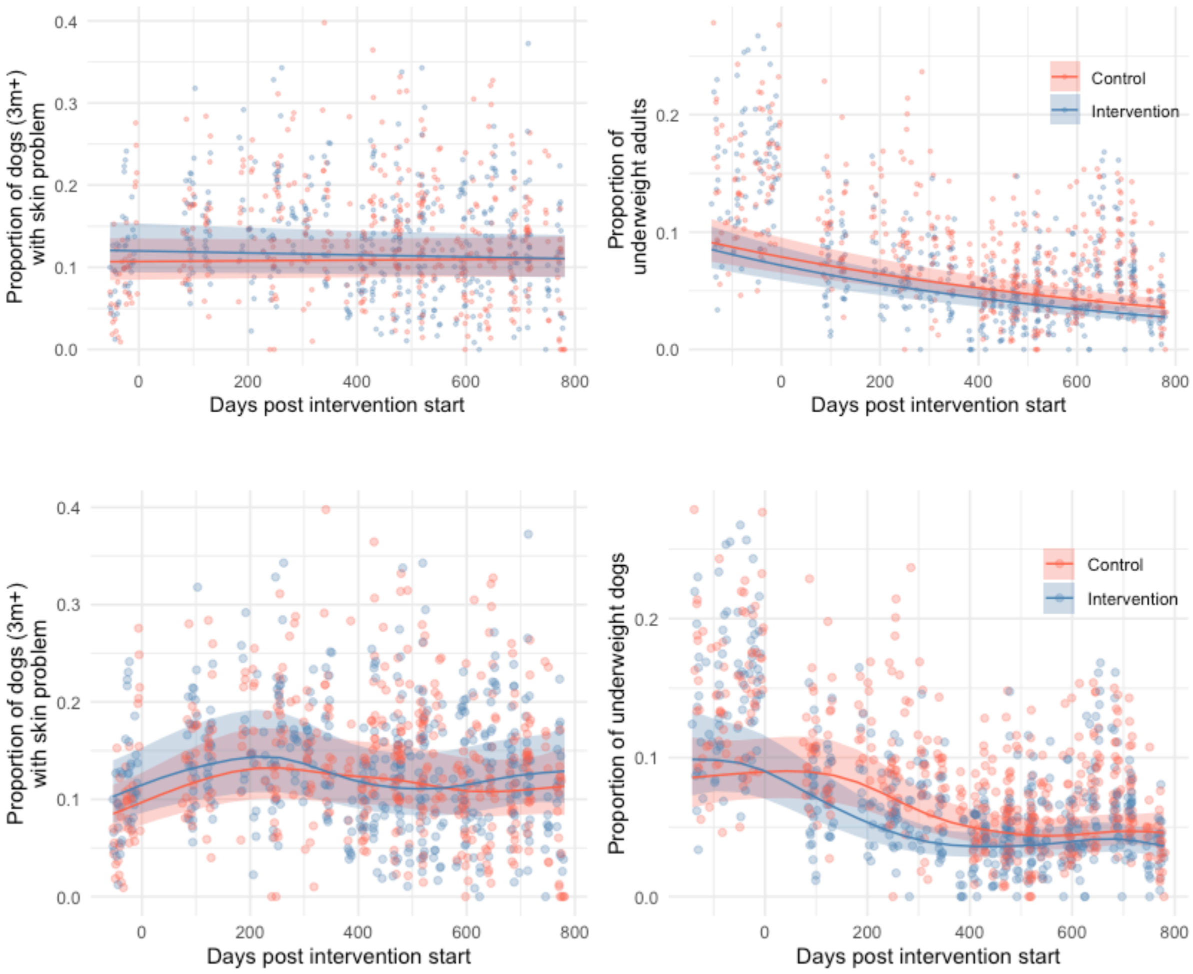


Figure S14. Estimated mean proportion of underweight dogs and those with a skin condition (mild, moderate or severe) from GLMMs 10 and 11 (top panel) and GAMMs (bottom panel) with 95% confidence intervals for intervention (blue line) and control sites (red line) over time. Points show the observed counts for each site survey. Predictions are based on an average of all random effects for morning surveys, not in monsoon season in January (where those variables are present in the models).

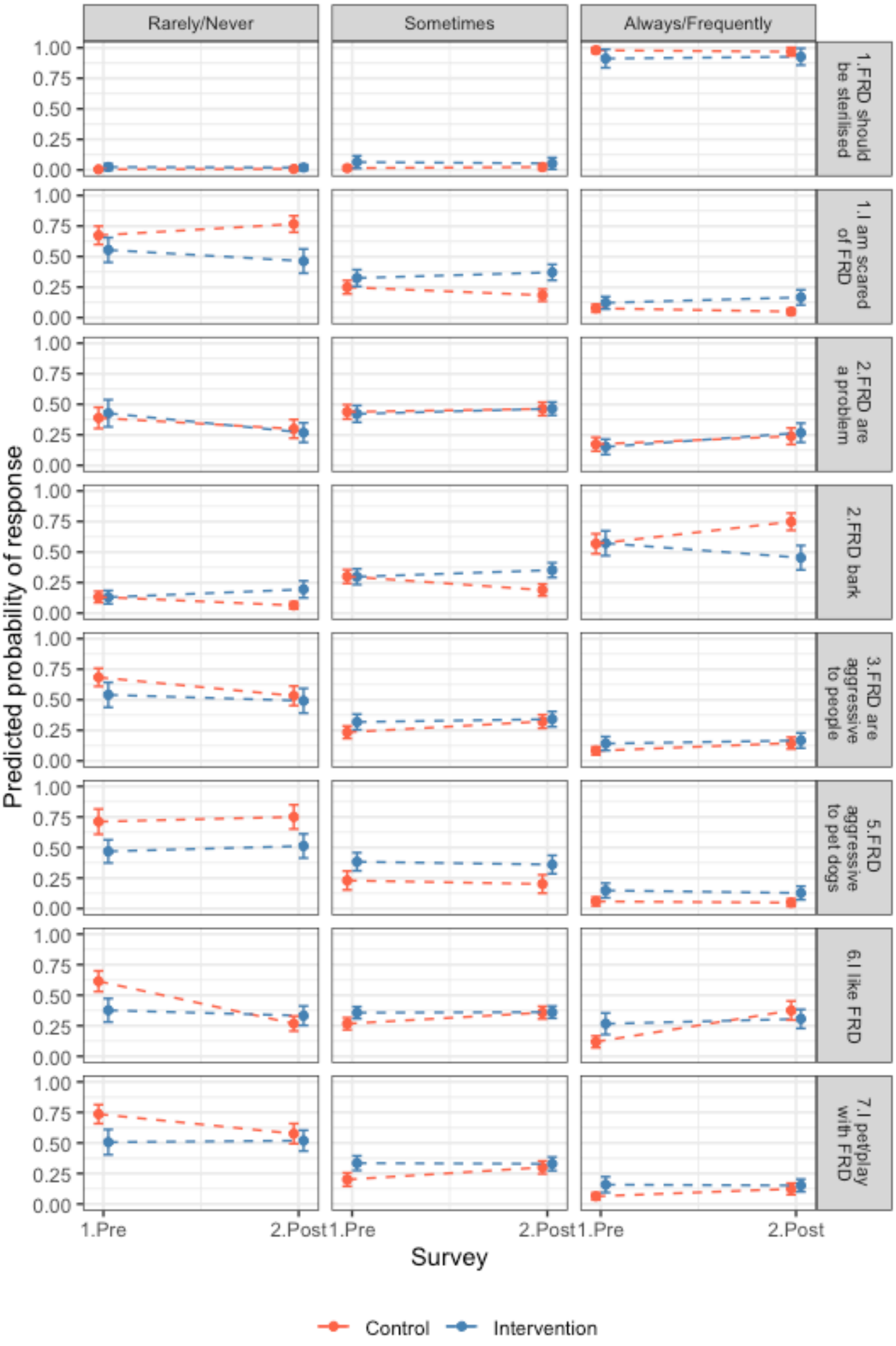
Rarely/Never
Sometimes
Always/Frequently
1.FRD should be sterilised
1.I am scared of FRD
2.FRD are a problem
2.FRD bark
3.FRD are aggressive to people
5.FRD aggressive to pet dogs
6.I like FRD
7.I pet/play with FRD
Predicted probability of response
1.00
0.75
0.50
0.25
0.00
1.Pre
2.Post
Survey
Control
Intervention

Figure S15. Opinions of free-roaming dogs. Responses of residents in intervention (blue) and control (red) sites to questionnaires before and after the intervention (surgical sterilisation) regarding free-roaming dogs (FRDs) in their area. Participants were asked to choose between always, frequently, sometimes, rarely or never regarding eight statements (1 per panel) and these were grouped for modelling. The predicted probability of response from ordinal models for individual questions is shown, e.g. an increase in the predicted probability of response in the 'Always/Frequently' panel for statement 2 from pre to post can be interpreted as more participants answering that they thought FRDs barked always or frequently in their area. Questionnaire counts in Table S12 and model outputs in Table S14.

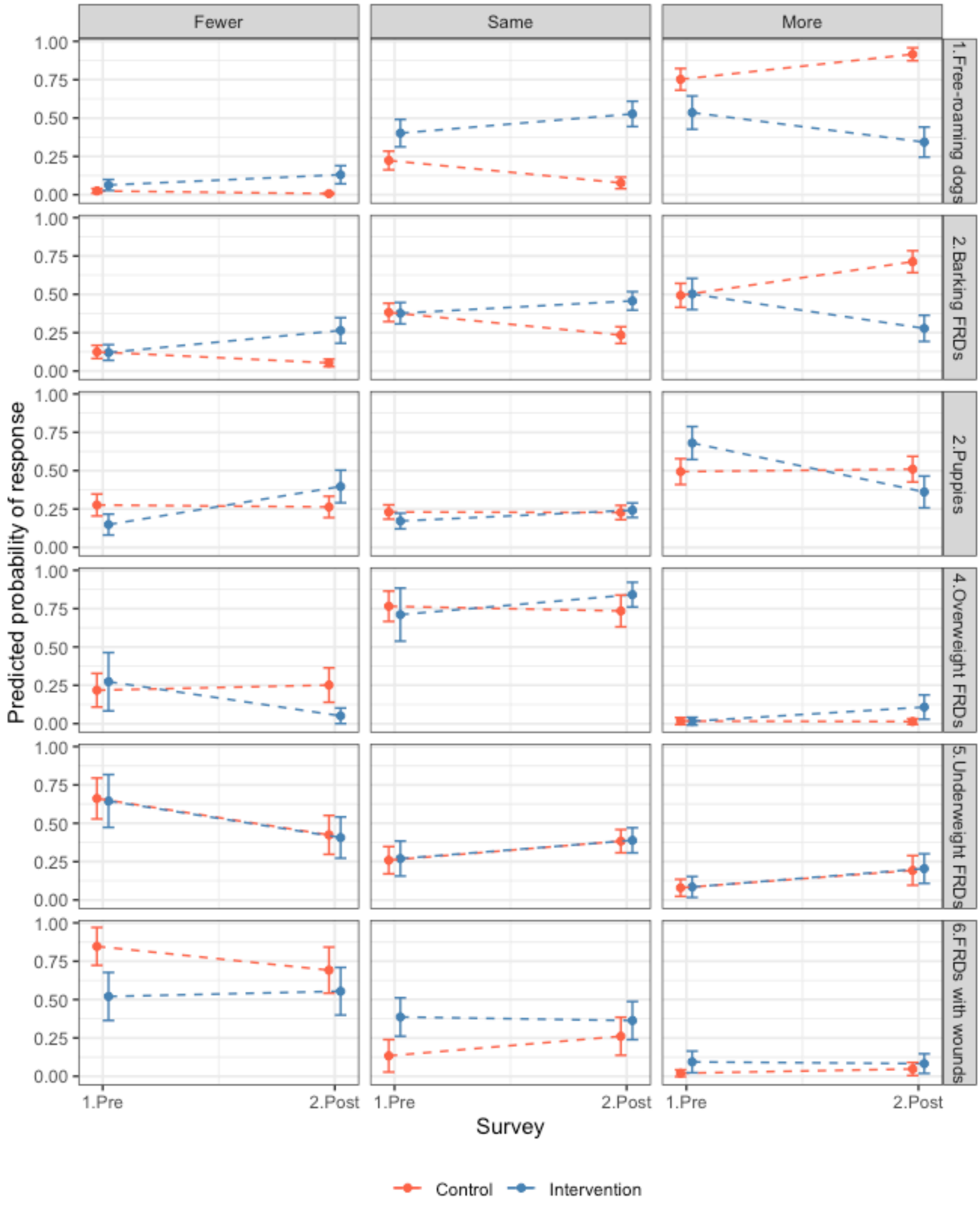
Fewer
Same
More
1.Free-roaming dogs
2.Barking FRDs
2.Puppies
4.Overweight FRDs
5.Underweight FRDs
6.FRDs with wounds
Predicted probability of response
1.00
0.75
0.50
0.25
0.00
1.Pre
2.Post
Survey
Control
Intervention

Figure S16. Community perceptions of free-roaming dog (FRD) numbers. Responses of residents in intervention (blue) and control (red) sites to questionnaires before and after the intervention (surgical sterilisation) regarding FRDs in their area. Questions presented start 'Compared to a year ago, have you noticed fewer, the same or more: e.g. 1. FRDs in your area?' The predicted probability of response from ordinal models for individual questions is shown, e.g. an increase in the predicted probability of response in the question 1 'more' panel from pre to post can be interpreted as more participants answering that they noticed more FRDs. Questionnaire response counts in Table S13 and model outputs in Table S15..

## 4. Supplementary Tables

| Variable | Options |
|---|---|
| Age category | Puppy (3 months or under), juvenile (4-11 months), adult (12 months or older) |
| Sex | Male, Female, Unknown |
| Lactation status (female only) | Lactating (this may include bitches close to parturition), non-lactating, unknown |
| Sterilisation status | Entire, sterilised (based on ear notch and/or presence of testicles) |
| Collar present | Yes, no. If yes, was it a rabies vaccination collar or owner collar. |
| Body condition score – recorded in standing adults only according to ICAM guidelines | 1 – emaciated, 2 – thin, 3 – ideal, 4 – overweight, 5 – obese. |
| Skin condition | None, mild (0-25% body affected by skin disease), moderate (25-50%), severe (>50% of body affected). |
| Any other health problems? | Lameness, wounds (if so, head, neck, backend, other), transmissible venereal tumour |

Table S1. Data collected about each free-roaming dog on count surveys, based on ICAM guidelines [3] and collected in a smartphone app.

| | Female | | Male | | |
|---|---|---|---|---|---|
| **Site code** | **Adult** | **Juvenile** | **Adult** | **Juvenile** | **Total** |
| 1N | 330 | 108 | 290 | 78 | 806 |
| 2N | 412 | 54 | 334 | 72 | 872 |
| 3N | 399 | 55 | 303 | 45 | 802 |
| 4N | 115 | 10 | 128 | 12 | 265 |
| 5N | 185 | 28 | 235 | 29 | 477 |
| Total | 1,441 | 255 | 1,290 | 236 | 3,222 |

Table S2. Number of sterilisations of FRDs performed in intervention sites between November 2020 and May 2021 split by age and sex (excluding cancelled surgeries).

| Model reference | | 1 | 2 | 3 | 4 | 5 | 6 | 7 | 8 | 9 | 10 | 11 | 12 | 13 |
|---|---|---|---|---|---|---|---|---|---|---|---|---|---|---|
| Response variable | | **Sterilised adult female count** | **Adult count** | **Juvenile count** | **Puppy count** | **Lactating female adult count** | **Unsterilised male count** | **Sterilised male count** | **Unsterilised female count** | **Sterilised female count** | **Underweight adult count (BCS 1 or 2)** | **Juvenile and adult with any skin problem count** | **Female juvenile count** | **Female adult count** |
| Offset | | Adult female count | Logged route length (km) | | | Logged adult female count | Logged route length (km) | | | | Adult count | Juvenile and adult count | Logged juvenile count (males + females) | Logged adult count (males + females) |
| Distribution | | Poisson | | | | | | | | | | | | |
| **Fixed variables** | | | | | | | | | | | | | | |
| Days after intervention : Site type (Control/Int) | SiteType (binary factor) | I | I | I | I | I | I | I | I | I | I | I | I | I |
| Time of day (am/pm) | am_pm (binary factor) | C | I | I | I | C | I | I | I | I | I | I | I | I |
| Month of year | month_adj (factor) | C | I | I | I | I | I | I | I | I | I | I | C | I |
| Rain during survey (yes/no) | rain_bin (binary factor) | C | I | I | C | C | I | I | I | I | I | C | C | C |
| Monsoon season (yes/no) | monsoon (binary factor) | C | I | I | C | C | I | I | I | C | I | I | C | C |
| Dogs per km of road surveyed | routeKM (numeric) | I | NA | NA | NA | C | NA | NA | NA | NA | I | I | C | I |
| **Interactions** | | | | | | | | | | | | | | |
| Time of day : month | am_pm* month_adj | NA | I | C | C | NA | I | I | C | C | C | C | C | C |
| Monsoon : rain | monsoon*rain_bin | NA | I | I | C | NA | C | C | C | C | C | C | C | C |

| Model reference | | 1 | 2 | 3 | 4 | 5 | 6 | 7 | 8 | 9 | 10 | 11 | 12 | 13 |
|---|---|---|---|---|---|---|---|---|---|---|---|---|---|---|
| **Random variables** | | | | | | | | | | | | | | |
| Individual level random effect | rpt_id (factor) | I | I | I | I | I | I | I | I | I | I | I | NA (not overdispersed) | I |
| Site number | study_site (factor) | NA | NA | NA | NA | I | NA | NA | I | NA | I | I | I | I |
| Days after intervention : site number | scale_dp* | I | I | I | I | C | I | I | C | I | C | C | C | C |
| | study_site | | | | | | | | | | | | | |
| n | | 787 | 939 | 939 | 939 | 939 | 787 | 787 | 787 | 787 | 939 | 865 | 924 | 939 |
| Primary periods included | | 5 to 14 | 2 to 14 | | | | 5 to 14 | | | | 2 to 14 | 3 to 14 | 2 to 14 | |
| Figure reference | | Fig. S | Fig. 3 (Fig. S10) | | | | | | | | Fig. S11,S14 | | | |
| Table reference | | Table S4 | Table S5 | | | | Table S6 | | | | Table S7 | | Table S8 | |

I – included in final model, C – considered.

Table S3. GLM model specifications including all considered and included variables.

| | Model 1: Adult sterilisation coverage | | |
|---|---|---|---|
| *Predictors* | *Incidence Rate Ratios* | *CI* | *p* |
| Intercept | 0.23 | 0.20 – 0.27 | **<0.001** |
| Days post intervention | 0.87 | 0.79 – 0.95 | **0.003** |
| Site type (Intervention) | 2.53 | 2.08 – 3.08 | **<0.001** |
| Dog density | 1.11 | 1.01 – 1.23 | **0.034** |
| Days post intervention X Site type (Intervention) | 1.02 | 0.90 – 1.15 | 0.799 |
| **Random Effects** | | | |
| $\sigma^2$ | 1.30 | | |
| $\tau_{00\ rpt\_id}$ | 0.01 | | |
| $\tau_{00\ study\_site}$ | 0.02 | | |
| $\tau_{11\ study\_site.scale\_dp}$ | 0.00 | | |
| $\rho_{01\ study\_site}$ | 0.16 | | |
| ICC | 0.02 | | |
| $N_{study\_site}$ | 10 | | |
| $N_{rpt\_id}$ | 787 | | |
| Observations | 787 | | |
| Marginal $R^2$ / Conditional $R^2$ | 0.140 / 0.157 | | |

Table S4. Estimated coefficients and 95% confidence intervals from sterilisation coverage Model 1 with count of sterilised adults as the response variable.

| | **Model 2: Adults per km** | | | **Model 3: Juveniles per km** | | | **Model 4: Puppies per km** | | | **Model 5: Lactating females** | | |
|---|---|---|---|---|---|---|---|---|---|---|---|---|
| *Predictors* | *Incidence Rate Ratios* | *CI* | *p* | *Incidence Rate Ratios* | *CI* | *p* | *Incidence Rate Ratios* | *CI* | *p* | *Incidence Rate Ratios* | *CI* | *p* |
| Intercept | 5.64 | 3.97 – 8.02 | **<0.001** | 0.43 | 0.26 – 0.71 | **0.001** | 0.79 | 0.44 – 1.39 | 0.410 | 0.16 | 0.13 – 0.18 | **<0.001** |
| Days post intervention | 0.88 | 0.83 – 0.94 | **<0.001** | 0.41 | 0.36 – 0.47 | **<0.001** | 0.83 | 0.71 – 0.98 | **0.030** | 1.01 | 0.94 – 1.09 | 0.758 |
| Site type (Intervention) | 0.95 | 0.58 – 1.55 | 0.838 | 0.93 | 0.47 – 1.84 | 0.836 | 0.86 | 0.40 – 1.89 | 0.712 | 0.80 | 0.71 – 0.91 | **0.001** |
| Afternoon survey | 0.74 | 0.68 – 0.80 | **<0.001** | 0.76 | 0.72 – 0.81 | **<0.001** | 0.84 | 0.78 – 0.90 | **<0.001** | | | |
| February | 0.92 | 0.85 – 0.99 | **0.035** | 1.20 | 0.98 – 1.48 | 0.077 | 0.51 | 0.42 – 0.63 | **<0.001** | 0.65 | 0.54 – 0.77 | **<0.001** |
| March | 0.87 | 0.81 – 0.94 | **<0.001** | 1.22 | 1.01 – 1.48 | **0.041** | 0.50 | 0.42 – 0.61 | **<0.001** | 0.61 | 0.51 – 0.73 | **<0.001** |
| April | 0.69 | 0.64 – 0.74 | **<0.001** | 1.08 | 0.89 – 1.30 | 0.439 | 0.28 | 0.24 – 0.34 | **<0.001** | 0.44 | 0.37 – 0.52 | **<0.001** |
| May | 0.57 | 0.51 – 0.64 | **<0.001** | 0.63 | 0.47 – 0.85 | **0.003** | 0.23 | 0.18 – 0.30 | **<0.001** | 0.45 | 0.36 – 0.56 | **<0.001** |
| June | 0.58 | 0.52 – 0.64 | **<0.001** | 0.62 | 0.46 – 0.83 | **0.001** | 0.19 | 0.15 – 0.24 | **<0.001** | 0.33 | 0.27 – 0.42 | **<0.001** |
| July | 0.62 | 0.57 – 0.68 | **<0.001** | 0.71 | 0.55 – 0.93 | **0.012** | 0.27 | 0.22 – 0.32 | **<0.001** | 0.44 | 0.37 – 0.53 | **<0.001** |
| August | 0.74 | 0.68 – 0.81 | **<0.001** | 0.73 | 0.56 – 0.95 | **0.021** | 0.27 | 0.23 – 0.33 | **<0.001** | 0.45 | 0.38 – 0.53 | **<0.001** |
| September | 0.78 | 0.72 – 0.86 | **<0.001** | 0.55 | 0.42 – 0.72 | **<0.001** | 0.32 | 0.27 – 0.39 | **<0.001** | 0.51 | 0.43 – 0.61 | **<0.001** |
| October | 0.90 | 0.83 – 0.97 | **0.005** | 0.66 | 0.53 – 0.81 | **<0.001** | 0.43 | 0.36 – 0.52 | **<0.001** | 0.67 | 0.56 – 0.79 | **<0.001** |
| November | 0.90 | 0.84 – 0.97 | **0.004** | 0.90 | 0.74 – 1.09 | 0.286 | 0.54 | 0.45 – 0.64 | **<0.001** | 0.86 | 0.74 – 1.01 | 0.059 |
| December | 1.00 | 0.90 – 1.10 | 0.976 | 1.20 | 0.91 – 1.58 | 0.191 | 1.02 | 0.78 – 1.34 | 0.873 | 0.89 | 0.71 – 1.12 | 0.311 |
| Rain on survey | 0.97 | 0.91 – 1.03 | 0.270 | 1.33 | 1.09 – 1.63 | **0.005** | | | | | | |
| Monsoon season | 1.10 | 1.04 – 1.16 | **<0.001** | 1.71 | 1.44 – 2.02 | **<0.001** | | | | | | |
| Days post intervention X Site type (Intervention) | 1.02 | 0.93 – 1.11 | 0.661 | 0.97 | 0.81 – 1.16 | 0.728 | 0.72 | 0.57 – 0.90 | **0.004** | 0.73 | 0.66 – 0.82 | **<0.001** |

| | Model 2: Adults per km | | | Model 3: Juveniles per km | | | Model 4: Puppies per km | | | Model 5: Lactating females | | |
|---|---|---|---|---|---|---|---|---|---|---|---|---|
| Afternoon X February | 0.92 | 0.82 – 1.02 | 0.107 | | | | | | | | | |
| Afternoon X March | 0.84 | 0.76 – 0.93 | **0.001** | | | | | | | | | |
| Afternoon X April | 0.93 | 0.84 – 1.03 | 0.164 | | | | | | | | | |
| Afternoon X May | 1.09 | 0.95 – 1.24 | 0.217 | | | | | | | | | |
| Afternoon X June | 1.14 | 1.02 – 1.28 | **0.026** | | | | | | | | | |
| Afternoon X July | 1.39 | 1.26 – 1.54 | **<0.001** | | | | | | | | | |
| Afternoon X August | 1.37 | 1.25 – 1.50 | **<0.001** | | | | | | | | | |
| Afternoon X September | 1.22 | 1.10 – 1.34 | **<0.001** | | | | | | | | | |
| Afternoon X October | 1.13 | 1.02 – 1.24 | **0.018** | | | | | | | | | |
| Afternoon X November | 1.05 | 0.96 – 1.15 | 0.327 | | | | | | | | | |
| Afternoon X December | 1.07 | 0.93 – 1.22 | 0.344 | | | | | | | | | |
| Rain on survey X Monsoon season | 0.92 | 0.86 – 0.99 | **0.024** | 0.69 | 0.55 – 0.86 | **0.001** | | | | | | |
| **Random Effects** | | | | | | | | | | | | |
| $\sigma^2$ | 0.24 | | | 2.65 | | | 2.05 | | | 2.78 | | |
| $\tau_{00}$ | 0.02 rpt_id | | | 0.13 rpt_id | | | 0.19 rpt_id | | | 0.09 rpt_id | | |
| | 0.16 study_site | | | 0.30 study_site | | | 0.39 study_site | | | 0.00 study_site | | |
| $\tau_{11}$ | 0.00 study_site.scale_dp | | | 0.01 study_site.scale_dp | | | 0.03 study_site.scale_dp | | | | | |
| $\rho_{01}$ | 0.43 study_site | | | -0.49 study_site | | | 0.01 study_site | | | | | |
| ICC | 0.43 | | | 0.09 | | | 0.17 | | | 0.00 | | |
| N | 10 study_site | | | 10 study_site | | | 10 study_site | | | 939 rpt_id | | |
| | 939 rpt_id | | | 939 rpt_id | | | 939 rpt_id | | | 10 study_site | | |

| | Model 2: Adults per km | Model 3: Juveniles per km | Model 4: Puppies per km | Model 5: Lactating females |
|---|---|---|---|---|
| Observations | 939 | 939 | 939 | 939 |
| Marginal $R^2$ / Conditional $R^2$ | 0.099 / 0.485 | 0.102 / 0.182 | 0.099 / 0.252 | 0.054 / 0.055 |

Table S5. Estimated coefficients and 95% confidence intervals for four GLMMs (Models 2-5) assessing the counts per km of adults, juveniles and puppies and the proportion of lactating females with site as a random effect.

| | Model 6: Unsterilised males per km | | | Model 7: Sterilised males per km | | | Model 8: Unsterilised females per km | | | Model 9: Sterilised females per km | | |
|---|---|---|---|---|---|---|---|---|---|---|---|---|
| *Predictors* | *Incidence Rate Ratios* | *CI* | *p* | *Incidence Rate Ratios* | *CI* | *p* | *Incidence Rate Ratios* | *CI* | *p* | *Incidence Rate Ratios* | *CI* | *p* |
| Intercept | 2.72 | 2.16 – 3.44 | **<0.001** | 0.52 | 0.29 – 0.96 | **0.036** | 1.45 | 0.93 – 2.27 | 0.104 | 0.51 | 0.27 – 0.97 | **0.040** |
| Days post intervention | 0.93 | 0.87 – 1.00 | **0.048** | 0.62 | 0.53 – 0.72 | **<0.001** | 0.91 | 0.85 – 0.97 | **0.004** | 0.67 | 0.59 – 0.77 | **<0.001** |
| Site type (Intervention) | 0.57 | 0.42 – 0.78 | **<0.001** | 2.20 | 0.96 – 5.05 | 0.062 | 0.51 | 0.28 – 0.96 | **0.036** | 2.15 | 0.87 – 5.30 | 0.097 |
| Afternoon survey | 0.73 | 0.66 – 0.80 | **<0.001** | 0.62 | 0.53 – 0.73 | **<0.001** | 0.87 | 0.84 – 0.90 | **<0.001** | 0.89 | 0.85 – 0.92 | **<0.001** |
| February | 0.91 | 0.82 – 1.01 | 0.064 | 0.73 | 0.62 – 0.85 | **<0.001** | 0.86 | 0.78 – 0.94 | **0.002** | 0.94 | 0.83 – 1.06 | 0.312 |
| March | 0.84 | 0.76 – 0.92 | **<0.001** | 0.82 | 0.71 – 0.95 | **0.009** | 0.79 | 0.72 – 0.87 | **<0.001** | 0.75 | 0.66 – 0.84 | **<0.001** |
| April | 0.60 | 0.55 – 0.66 | **<0.001** | 0.73 | 0.63 – 0.84 | **<0.001** | 0.66 | 0.60 – 0.72 | **<0.001** | 0.74 | 0.66 – 0.84 | **<0.001** |
| May | 0.50 | 0.43 – 0.58 | **<0.001** | 0.63 | 0.51 – 0.78 | **<0.001** | 0.56 | 0.48 – 0.65 | **<0.001** | 0.61 | 0.53 – 0.71 | **<0.001** |
| June | 0.50 | 0.44 – 0.58 | **<0.001** | 0.56 | 0.45 – 0.69 | **<0.001** | 0.58 | 0.50 – 0.67 | **<0.001** | 0.76 | 0.66 – 0.88 | **<0.001** |
| July | 0.53 | 0.47 – 0.61 | **<0.001** | 0.60 | 0.49 – 0.73 | **<0.001** | 0.72 | 0.63 – 0.83 | **<0.001** | 0.92 | 0.81 – 1.04 | 0.194 |
| August | 0.65 | 0.58 – 0.74 | **<0.001** | 0.62 | 0.51 – 0.75 | **<0.001** | 0.81 | 0.71 – 0.93 | **0.003** | 1.06 | 0.93 – 1.20 | 0.378 |
| September | 0.76 | 0.66 – 0.87 | **<0.001** | 0.64 | 0.52 – 0.79 | **<0.001** | 0.85 | 0.74 – 0.97 | **0.018** | 1.15 | 1.01 – 1.32 | **0.039** |
| October | 0.88 | 0.79 – 0.97 | **0.011** | 0.85 | 0.72 – 1.01 | 0.063 | 0.93 | 0.84 – 1.03 | 0.187 | 1.17 | 1.04 – 1.33 | **0.011** |
| November | 0.94 | 0.85 – 1.03 | 0.172 | 0.77 | 0.67 – 0.89 | **0.001** | 0.94 | 0.86 – 1.03 | 0.171 | 0.94 | 0.84 – 1.06 | 0.336 |
| December | 1.02 | 0.90 – 1.16 | 0.709 | 0.87 | 0.70 – 1.08 | 0.208 | 1.08 | 0.95 – 1.23 | 0.215 | 0.86 | 0.72 – 1.02 | 0.083 |
| Rain on survey | 0.92 | 0.88 – 0.96 | **<0.001** | 0.90 | 0.84 – 0.96 | **0.003** | 0.92 | 0.87 – 0.97 | **0.002** | 0.90 | 0.85 – 0.97 | **0.003** |
| Monsoon season | 1.09 | 1.01 – 1.17 | **0.026** | 1.11 | 1.00 – 1.25 | 0.055 | 1.10 | 1.00 – 1.20 | **0.040** | | | |
| Days post intervention X Site type (Intervention) | 1.22 | 1.11 – 1.35 | **<0.001** | 1.33 | 1.09 – 1.63 | **0.004** | 1.15 | 1.05 – 1.26 | **0.002** | 1.11 | 0.93 – 1.32 | 0.239 |

| | Model 6: Unsterilised males per km | | | Model 7: Sterilised males per km | | | Model 8: Unsterilised females per km | | | Model 9: Sterilised females per km | | |
|---|---|---|---|---|---|---|---|---|---|---|---|---|
| Afternoon X February | 0.88 | 0.77 – 1.01 | 0.067 | 1.11 | 0.89 – 1.37 | 0.357 | | | | | | |
| Afternoon X March | 0.79 | 0.69 – 0.90 | **<0.001** | 0.93 | 0.75 – 1.14 | 0.475 | | | | | | |
| Afternoon X April | 0.91 | 0.80 – 1.03 | 0.127 | 1.06 | 0.86 – 1.30 | 0.581 | | | | | | |
| Afternoon X May | 1.11 | 0.94 – 1.31 | 0.235 | 1.19 | 0.93 – 1.52 | 0.157 | | | | | | |
| Afternoon X June | 1.08 | 0.93 – 1.26 | 0.286 | 1.29 | 1.03 – 1.63 | **0.029** | | | | | | |
| Afternoon X July | 1.30 | 1.14 – 1.49 | **<0.001** | 1.60 | 1.29 – 1.99 | **<0.001** | | | | | | |
| Afternoon X August | 1.27 | 1.12 – 1.44 | **<0.001** | 1.68 | 1.37 – 2.05 | **<0.001** | | | | | | |
| Afternoon X September | 1.14 | 1.00 – 1.31 | 0.054 | 1.55 | 1.25 – 1.92 | **<0.001** | | | | | | |
| Afternoon X October | 1.12 | 0.98 – 1.28 | 0.105 | 1.19 | 0.94 – 1.51 | 0.153 | | | | | | |
| Afternoon X November | 1.04 | 0.91 – 1.17 | 0.584 | 1.25 | 1.03 – 1.53 | **0.025** | | | | | | |
| Afternoon X December | 0.95 | 0.80 – 1.12 | 0.512 | 1.60 | 1.20 – 2.14 | **0.001** | | | | | | |
| **Random Effects** | | | | | | | | | | | | |
| $\sigma^2$ | 0.52 | | | 0.93 | | | 0.87 | | | 1.00 | | |
| $\tau_{00}$ | 0.02 rpt_id | | | 0.04 rpt_id | | | 0.04 rpt_id | | | 0.05 rpt_id | | |
| | 0.06 study_site | | | 0.44 study_site | | | 0.25 study_site | | | 0.52 study_site | | |
| $\tau_{11}$ | 0.00 study_site.scale_dp | | | 0.02 study_site.scale_dp | | | | | | 0.01 study_site.scale_dp | | |
| $\rho_{01}$ | 0.44 study_site | | | 0.58 study_site | | | | | | -0.10 study_site | | |
| ICC | 0.12 | | | 0.37 | | | 0.22 | | | 0.34 | | |
| N | 10 study_site | | | 10 study_site | | | 10 study_site | | | 10 study_site | | |
| | 787 rpt_id | | | 787 rpt_id | | | 787 rpt_id | | | 787 rpt_id | | |
| Observations | 787 | | | 787 | | | 787 | | | 787 | | |

| | Model 6: Unsterilised males per km | Model 7: Sterilised males per km | Model 8: Unsterilised females per km | Model 9: Sterilised females per km |
|---|---|---|---|---|
| Marginal $R^2$ / Conditional $R^2$ | 0.158 / 0.263 | 0.182 / 0.484 | 0.086 / 0.288 | 0.128 / 0.426 |

Table S6. Estimated coefficients and 95% confidence intervals for four GLMMs (6-9) assessing counts per km of entire adult males, sterilised adult males, entire adult females and sterilised adult females.

| | Model 10: Underweight adults | | | Model 11: Adults and juveniles with a skin condition | | |
|---|---|---|---|---|---|---|
| *Predictors* | *Incidence Rate Ratios* | *CI* | *p* | *Incidence Rate Ratios* | *CI* | *p* |
| Intercept | 0.07 | 0.06 – 0.09 | **<0.001** | 0.10 | 0.08 – 0.12 | **<0.001** |
| Days post intervention | 0.63 | 0.59 – 0.67 | **<0.001** | 1.02 | 0.93 – 1.11 | 0.688 |
| Site type (Intervention) | 0.91 | 0.76 – 1.08 | 0.287 | 1.12 | 0.86 – 1.46 | 0.407 |
| February | 1.05 | 0.88 – 1.26 | 0.572 | 1.20 | 1.01 – 1.42 | **0.038** |
| March | 1.20 | 1.01 – 1.42 | **0.039** | 1.21 | 1.03 – 1.42 | **0.022** |
| April | 1.14 | 0.96 – 1.35 | 0.130 | 1.23 | 1.05 – 1.43 | **0.011** |
| May | 1.63 | 1.26 – 2.11 | **<0.001** | 0.97 | 0.75 – 1.26 | 0.843 |
| June | 1.83 | 1.42 – 2.35 | **<0.001** | 0.94 | 0.74 – 1.21 | 0.645 |
| July | 1.97 | 1.57 – 2.46 | **<0.001** | 0.95 | 0.76 – 1.19 | 0.652 |
| August | 1.89 | 1.51 – 2.36 | **<0.001** | 0.97 | 0.77 – 1.20 | 0.756 |
| September | 2.42 | 1.93 – 3.03 | **<0.001** | 1.26 | 1.00 – 1.58 | 0.052 |
| October | 2.28 | 1.92 – 2.70 | **<0.001** | 1.00 | 0.85 – 1.18 | 0.983 |
| November | 1.72 | 1.46 – 2.01 | **<0.001** | 1.08 | 0.93 – 1.27 | 0.312 |
| December | 1.08 | 0.85 – 1.38 | 0.522 | 0.92 | 0.74 – 1.16 | 0.491 |
| Afternoon survey | 1.09 | 1.03 – 1.16 | **0.002** | 1.07 | 1.00 – 1.13 | **0.036** |
| Monsoon season | 0.79 | 0.69 – 0.91 | **0.001** | 1.15 | 0.99 – 1.33 | 0.068 |
| Rain on survey | 0.92 | 0.85 – 0.99 | **0.033** | | | |
| Dogs per km (scaled) | 1.15 | 1.06 – 1.26 | **0.001** | 1.23 | 1.09 – 1.40 | **0.001** |
| Days post intervention X Site type (Intervention) | 0.91 | 0.83 – 1.00 | **0.049** | 0.93 | 0.83 – 1.06 | 0.277 |
| **Random Effects** | | | | | | |
| $\sigma^2$ | 2.85 | | | 2.39 | | |
| $\tau_{00}$ | 0.09 $_{rpt\_id}$ | | | 0.15 $_{rpt\_id}$ | | |
| | 0.01 $_{study\_site}$ | | | 0.04 $_{study\_site}$ | | |
| ICC | 0.01 | | | 0.01 | | |
| N | 10 $_{study\_site}$ | | | 10 $_{study\_site}$ | | |
| | 939 $_{rpt\_id}$ | | | 865 $_{rpt\_id}$ | | |
| Observations | 939 | | | 865 | | |
| Marginal $R^2$ / Conditional $R^2$ | 0.065 / 0.070 | | | 0.024 / 0.038 | | |

Table S7. Estimated coefficients and 95% confidence intervals from two GLMMs for underweight dogs (Model 10) and dogs with a skin condition (Model 11) as response variables

| | Model 12: Juvenile females | | | Model 13: Adult females | | |
|---|---|---|---|---|---|---|
| *Predictors* | *Incidence Rate Ratios* | *CI* | *p* | *Incidence Rate Ratios* | *CI* | *p* |
| Intercept | 0.54 | 0.51 – 0.58 | **<0.001** | 0.35 | 0.32 – 0.37 | **<0.001** |
| Days post intervention | 1.07 | 1.00 – 1.14 | **0.039** | 1.03 | 1.01 – 1.05 | **0.009** |
| Site type (Intervention) | 0.96 | 0.89 – 1.05 | 0.391 | 1.08 | 0.99 – 1.18 | 0.076 |
| Afternoon survey | 1.05 | 1.00 – 1.10 | 0.070 | 1.07 | 1.05 – 1.08 | **<0.001** |
| Days post intervention X Site type (Intervention) | 0.96 | 0.88 – 1.05 | 0.431 | 0.99 | 0.97 – 1.02 | 0.654 |
| February | | | | 1.07 | 1.02 – 1.13 | **0.006** |
| March | | | | 1.07 | 1.02 – 1.13 | **0.004** |
| April | | | | 1.13 | 1.08 – 1.18 | **<0.001** |
| May | | | | 1.02 | 0.96 – 1.08 | 0.605 |
| June | | | | 1.12 | 1.06 – 1.18 | **<0.001** |
| July | | | | 1.11 | 1.06 – 1.17 | **<0.001** |
| August | | | | 1.12 | 1.07 – 1.17 | **<0.001** |
| September | | | | 1.08 | 1.03 – 1.13 | **0.001** |
| October | | | | 1.02 | 0.98 – 1.07 | 0.338 |
| November | | | | 1.02 | 0.97 – 1.07 | 0.401 |
| December | | | | 1.00 | 0.93 – 1.08 | 0.940 |
| Dogs per km (scaled) | | | | 1.12 | 1.07 – 1.17 | **<0.001** |
| **Random Effects** | | | | | | |
| $\sigma^2$ | 1.03 | | | 0.00 | | |
| $\tau_{00}$ | 0.00 study_site | | | 0.00 rpt_id | | |
| | | | | 0.00 study_site | | |
| ICC | 0.00 | | | 0.59 | | |
| N | 10 study_site | | | 10 study_site | | |
| | | | | 939 rpt_id | | |
| Observations | 924 | | | 939 | | |
| Marginal $R^2$ / Conditional $R^2$ | 0.002 / 0.003 | | | 0.680 / 0.868 | | |

Table S8. Estimated coefficients and 95% confidence intervals from Model 12 female juveniles and Model 13 female adults as response variables.

| | GAMM 1: Adults per km | | | GAMM 2: Juveniles per km | | | GAMM 3: Puppies per km | | | GAMM 4: Proportion lactating females | | |
|---|---|---|---|---|---|---|---|---|---|---|---|---|
| *Predictors* | *Incidence Rate Ratios* | *CI* | *p* | *Incidence Rate Ratios* | *CI* | *p* | *Incidence Rate Ratios* | *CI* | *p* | *Incidence Rate Ratios* | *CI* | *p* |
| Intercept | 4.05 | 2.72 – 6.03 | **<0.001** | 0.23 | 0.13 – 0.38 | **<0.001** | 0.29 | 0.16 – 0.52 | **<0.001** | 0.09 | 0.09 – 0.10 | **<0.001** |
| Site type (Intervention) | 0.97 | 0.55 – 1.70 | 0.904 | 0.92 | 0.45 – 1.90 | 0.825 | 0.66 | 0.29 – 1.50 | 0.319 | 0.61 | 0.54 – 0.69 | **<0.001** |
| Monsoon season | 1.09 | 1.04 – 1.14 | **<0.001** | 1.04 | 0.92 – 1.18 | 0.536 | | | | | | |
| Rain on survey | 0.91 | 0.89 – 0.94 | **<0.001** | 0.91 | 0.84 – 0.98 | **0.019** | | | | | | |
| Afternoon survey | 0.81 | 0.79 – 0.83 | **<0.001** | 0.77 | 0.73 – 0.82 | **<0.001** | 0.82 | 0.78 – 0.87 | **<0.001** | | | |
| Days post neutering X Site type (Control) | | | **<0.001** | | | **<0.001** | | | **<0.001** | | | **<0.001** |
| Days post neutering X Site type (Neuter) | | | **<0.001** | | | **<0.001** | | | **<0.001** | | | **<0.001** |
| Study site | | | **<0.001** | | | **<0.001** | | | **<0.001** | | | **<0.001** |
| Observations | 939 | | | 939 | | | 939 | | | 939 | | |
| $R^2$ | 0.897 | | | 0.720 | | | 0.743 | | | 0.748 | | |

Table S9. Estimated coefficients and 95% confidence intervals for four GAMMs assessing the counts per km of adults, juveniles and puppies and the proportion of lactating females with site as a random effect.

| | GAMM 1: Unsterilised males per km | | | GAMM 2: Sterilised males per km | | | GAMM 3: Unsterilised females per km | | | GAMM 4: Sterilised females per km | | |
|---|---|---|---|---|---|---|---|---|---|---|---|---|
| *Predictors* | *Incidence Rate Ratios* | *CI* | *p* | *Incidence Rate Ratios* | *CI* | *p* | *Incidence Rate Ratios* | *CI* | *p* | *Incidence Rate Ratios* | *CI* | *p* |
| Intercept | 1.93 | 1.49 – 2.48 | **<0.001** | 0.26 | 0.13 – 0.51 | **<0.001** | 1.10 | 0.69 – 1.77 | 0.679 | 0.32 | 0.16 – 0.63 | **0.001** |
| Site type (Intervention) | 0.69 | 0.48 – 0.98 | **0.040** | 2.85 | 1.06 – 7.65 | **0.038** | 0.58 | 0.30 – 1.14 | 0.113 | 2.33 | 0.90 – 6.06 | 0.082 |
| Afternoon survey | 0.75 | 0.73 – 0.77 | **<0.001** | 0.78 | 0.75 – 0.81 | **<0.001** | 0.86 | 0.83 – 0.89 | **<0.001** | 0.88 | 0.84 – 0.91 | **<0.001** |
| Days post neutering X Site type (Control) | | | **<0.001** | | | **<0.001** | | | **<0.001** | | | **<0.001** |
| Days post neutering X Site type (Neuter) | | | **<0.001** | | | **<0.001** | | | **<0.001** | | | **<0.001** |
| Study site | | | **<0.001** | | | **<0.001** | | | **<0.001** | | | **<0.001** |
| Observations | 787 | | | 787 | | | 787 | | | 787 | | |
| $R^2$ | 0.819 | | | 0.812 | | | 0.852 | | | 0.831 | | |

Table S10. Estimated coefficients and 95% confidence intervals for four GAMMs assessing the counts per km of unsterilised males, sterilised males, unsterilised females and sterilised females with site as a random effect.

| Model | Residual degrees of freedom | Residual deviance | Degrees of freedom | Deviance | *p*-value |
|---|---|---|---|---|---|
| **Adult GAM with site type** | 901.917585 | 4974.66 | NA | NA | NA |
| **Adult GAM without site type** | 913.9255923 | 5052.107651 | -12.01 | -77.45 | 0.2941 |
| **Juvenile GAM with site type** | 899.9949961 | 2381.895472 | NA | NA | NA |
| **Juvenile GAM without site type** | 912.0083006 | 2431.898918 | -12.01 | -50.00 | 0.0788 |
| **Puppy GAM with site type** | 894.9211472 | 3004.84247 | NA | NA | NA |
| **Puppy GAM without site type** | 909.3797142 | 3303.263113 | -14.46 | -298.42 | **1.60E-13** |
| **Lactating female GAM with site type** | 906.9725103 | 1347.277293 | NA | NA | NA |
| **Lactating female GAM without site type** | 917.8321715 | 1452.469677 | -10.86 | -105.19 | **2.16E-11** |
| **Unsterilised adult male GAM with site type** | 757.9463846 | 2438.814657 | NA | NA | NA |
| **Unsterilised adult male GAM without site type** | 766.9411653 | 2573.313334 | -8.99 | -134.50 | **3.83E-06** |
| **Sterilised adult male GAM with site type** | 759.7563308 | 2007.585785 | NA | NA | NA |
| **Sterilised adult male GAM without site type** | 767.8796549 | 2068.808236 | -8.12 | -61.22 | **0.0043** |
| **Unsterilised adult female GAM with site type** | 757.9677861 | 2135.184597 | NA | NA | NA |
| **Unsterilised adult female GAM without site type** | 766.92 | 2172.720212 | -8.95 | -37.54 | 0.1409 |
| **Sterilised adult female GAM with site type** | 761.7599816 | 2237.973116 | NA | NA | NA |
| **Sterilised adult female GAM without site type** | 768.8990383 | 2268.992831 | -7.14 | -31.02 | 0.1680 |

Table S11. Analysis of variance in residual deviance between generalised additive mixed models (GAMMs) with and without site type to test significance of the intervention and control sites using a $Chi^2$ distribution. *P*-values below 0.05 indicate site type was significant in the model and suggest a difference between dog counts in control and intervention sites, as demonstrated in the puppies, lactating females, unsterilised and sterilised male GAMMs.

| | Control | | | | Neuter | | | | | |
|---|---|---|---|---|---|---|---|---|---|---|
| | Pre-sterilisation | | Post-sterilisation | | Pre-sterilisation | | Post-sterilisation | | | |
| | n | Proportion | n | Proportion | n | Proportion | n | Proportion | | |
| Variable | n_Control_1 | prop_Control_1 | n_Control_2 | prop_Control_2 | n_Neuter_1 | prop_Neuter_1 | n_Neuter_2 | prop_Neuter_2 | n | Proportion (95% CI) |
| **1.I am scared by stray dogs** | | | | | | | | | | |
| Always | 19 | 0.10 | 3 | 0.02 | 14 | 0.12 | 17 | 0.14 | 53 | 0.08 (0.06-0.11) |
| Frequently | 4 | 0.02 | 2 | 0.01 | 7 | 0.06 | 8 | 0.07 | 21 | 0.03 (0.02-0.05) |
| Sometimes | 45 | 0.23 | 51 | 0.26 | 31 | 0.26 | 37 | 0.31 | 164 | 0.26 (0.22-0.29) |
| Rarely | 41 | 0.21 | 64 | 0.32 | 15 | 0.13 | 14 | 0.12 | 134 | 0.21 (0.18-0.24) |
| Never | 90 | 0.45 | 79 | 0.40 | 52 | 0.44 | 43 | 0.36 | 264 | 0.42 (0.38-0.45) |
| **2.The stray dogs in this area are a problem** | | | | | | | | | | |
| Always | 17 | 0.09 | 19 | 0.10 | 18 | 0.15 | 22 | 0.18 | 76 | 0.12 (0.1-0.15) |
| Frequently | 21 | 0.11 | 29 | 0.15 | 10 | 0.08 | 20 | 0.17 | 80 | 0.13 (0.1-0.15) |
| Sometimes | 91 | 0.46 | 96 | 0.48 | 39 | 0.33 | 36 | 0.30 | 262 | 0.41 (0.37-0.45) |
| Rarely | 51 | 0.26 | 45 | 0.23 | 18 | 0.15 | 18 | 0.15 | 132 | 0.21 (0.18-0.24) |
| Never | 19 | 0.10 | 10 | 0.05 | 34 | 0.29 | 23 | 0.19 | 86 | 0.14 (0.11-0.16) |
| **3.Stray dogs bark in this area** | | | | | | | | | | |

| | Control | | | | Neuter | | | | | |
|---|---|---|---|---|---|---|---|---|---|---|
| | Pre-sterilisation | | Post-sterilisation | | Pre-sterilisation | | Post-sterilisation | | | |
| | n | Proportion | n | Proportion | n | Proportion | n | Proportion | | |
| Variable | n_Control_1 | prop_Control_1 | n_Control_2 | prop_Control_2 | n_Neuter_1 | prop_Neuter_1 | n_Neuter_2 | prop_Neuter_2 | n | Proportion (95% CI) |
| Always | 106 | 0.53 | 121 | 0.61 | 57 | 0.48 | 36 | 0.30 | 320 | 0.5 (0.46-0.54) |
| Frequently | 11 | 0.06 | 17 | 0.09 | 9 | 0.08 | 17 | 0.14 | 54 | 0.08 (0.06-0.11) |
| Sometimes | 39 | 0.20 | 50 | 0.25 | 34 | 0.29 | 40 | 0.34 | 163 | 0.26 (0.22-0.29) |
| Rarely | 12 | 0.06 | 8 | 0.04 | 12 | 0.10 | 13 | 0.11 | 45 | 0.07 (0.05-0.09) |
| Never | 31 | 0.16 | 3 | 0.02 | 7 | 0.06 | 13 | 0.11 | 54 | 0.08 (0.06-0.11) |
| **4.Stray dogs in this area are aggressive to people** | | | | | | | | | | |
| Always | 9 | 0.05 | 19 | 0.10 | 16 | 0.13 | 18 | 0.15 | 62 | 0.1 (0.08-0.12) |
| Frequently | 6 | 0.03 | 20 | 0.10 | 7 | 0.06 | 7 | 0.06 | 40 | 0.06 (0.05-0.09) |
| Sometimes | 57 | 0.29 | 53 | 0.27 | 31 | 0.26 | 34 | 0.29 | 175 | 0.28 (0.24-0.31) |
| Rarely | 40 | 0.20 | 61 | 0.31 | 17 | 0.14 | 26 | 0.22 | 144 | 0.23 (0.19-0.26) |
| Never | 87 | 0.44 | 46 | 0.23 | 48 | 0.40 | 34 | 0.29 | 215 | 0.34 (0.3-0.38) |
| **5.Stray dogs in this area are aggressive to other stray dogs** | | | | | | | | | | |
| Always | 47 | 0.24 | 24 | 0.12 | 25 | 0.21 | 13 | 0.11 | 109 | 0.17 (0.14-0.2) |

| | Control | | | | Neuter | | | | | |
|---|---|---|---|---|---|---|---|---|---|---|
| | Pre-sterilisation | | Post-sterilisation | | Pre-sterilisation | | Post-sterilisation | | | |
| | n | Proportion | n | Proportion | n | Proportion | n | Proportion | | |
| Variable | n_Control_1 | prop_Control_1 | n_Control_2 | prop_Control_2 | n_Neuter_1 | prop_Neuter_1 | n_Neuter_2 | prop_Neuter_2 | n | Proportion (95% CI) |
| Frequently | 19 | 0.10 | 40 | 0.20 | 14 | 0.12 | 20 | 0.17 | 93 | 0.15 (0.12-0.18) |
| Sometimes | 101 | 0.51 | 115 | 0.58 | 57 | 0.48 | 58 | 0.49 | 331 | 0.52 (0.48-0.56) |
| Rarely | 27 | 0.14 | 19 | 0.10 | 16 | 0.13 | 20 | 0.17 | 82 | 0.13 (0.1-0.16) |
| Never | 5 | 0.03 | 1 | 0.01 | 7 | 0.06 | 8 | 0.07 | 21 | 0.03 (0.02-0.05) |
| **6.Stray dogs in this area are aggressive to pet dogs** | | | | | | | | | | |
| Always | 2 | 0.01 | 2 | 0.01 | 6 | 0.05 | 12 | 0.10 | 22 | 0.03 (0.02-0.05) |
| Frequently | 2 | 0.01 | 5 | 0.03 | 7 | 0.06 | 8 | 0.07 | 22 | 0.03 (0.02-0.05) |
| Sometimes | 19 | 0.10 | 30 | 0.15 | 42 | 0.35 | 36 | 0.30 | 127 | 0.2 (0.17-0.23) |
| Rarely | 28 | 0.14 | 38 | 0.19 | 16 | 0.13 | 28 | 0.24 | 110 | 0.17 (0.14-0.21) |
| Never | 27 | 0.14 | 124 | 0.62 | 30 | 0.25 | 35 | 0.29 | 216 | 0.34 (0.3-0.38) |
| Unknown | 121 | 0.61 | 0 | 0.00 | 18 | 0.15 | 0 | 0.00 | 139 | 0.22 (0.19-0.25) |
| **7.I like stray dogs** | | | | | | | | | | |
| Always | 22 | 0.11 | 21 | 0.11 | 31 | 0.26 | 28 | 0.24 | 102 | 0.16 (0.13-0.19) |

| | Control | | | | Neuter | | | | | |
|---|---|---|---|---|---|---|---|---|---|---|
| | Pre-sterilisation | | Post-sterilisation | | Pre-sterilisation | | Post-sterilisation | | | |
| | n | Proportion | n | Proportion | n | Proportion | n | Proportion | | |
| Variable | n_Control_1 | prop_Control_1 | n_Control_2 | prop_Control_2 | n_Neuter_1 | prop_Neuter_1 | n_Neuter_2 | prop_Neuter_2 | n | Proportion (95% CI) |
| Frequently | 4 | 0.02 | 26 | 0.13 | 8 | 0.07 | 15 | 0.13 | 53 | 0.08 (0.06-0.11) |
| Sometimes | 42 | 0.21 | 106 | 0.53 | 26 | 0.22 | 30 | 0.25 | 204 | 0.32 (0.28-0.36) |
| Rarely | 97 | 0.49 | 42 | 0.21 | 28 | 0.24 | 30 | 0.25 | 197 | 0.31 (0.27-0.35) |
| Never | 34 | 0.17 | 4 | 0.02 | 26 | 0.22 | 16 | 0.13 | 80 | 0.13 (0.1-0.15) |
| **8.I pet or play with stray dogs** | | | | | | | | | | |
| Always | 7 | 0.04 | 7 | 0.04 | 12 | 0.10 | 11 | 0.09 | 37 | 0.06 (0.04-0.08) |
| Frequently | 1 | 0.01 | 8 | 0.04 | 7 | 0.06 | 8 | 0.07 | 24 | 0.04 (0.02-0.06) |
| Sometimes | 34 | 0.17 | 56 | 0.28 | 32 | 0.27 | 38 | 0.32 | 160 | 0.25 (0.22-0.29) |
| Rarely | 51 | 0.26 | 51 | 0.26 | 17 | 0.14 | 16 | 0.13 | 135 | 0.21 (0.18-0.25) |
| Never | 106 | 0.53 | 77 | 0.39 | 51 | 0.43 | 46 | 0.39 | 280 | 0.44 (0.4-0.48) |
| **9.Stray dogs should be sterilised** | | | | | | | | | | |
| Always | 187 | 0.94 | 173 | 0.87 | 96 | 0.81 | 94 | 0.79 | 550 | 0.86 (0.84-0.89) |
| Frequently | 3 | 0.02 | 11 | 0.06 | 5 | 0.04 | 7 | 0.06 | 26 | 0.04 (0.03-0.06) |

| | Control | | | | Neuter | | | | | |
|---|---|---|---|---|---|---|---|---|---|---|
| | Pre-sterilisation | | Post-sterilisation | | Pre-sterilisation | | Post-sterilisation | | | |
| | n | Proportion | n | Proportion | n | Proportion | n | Proportion | | |
| Variable | n_Control_1 | prop_Control_1 | n_Control_2 | prop_Control_2 | n_Neuter_1 | prop_Neuter_1 | n_Neuter_2 | prop_Neuter_2 | n | Proportion (95% CI) |
| Sometimes | 3 | 0.02 | 13 | 0.07 | 8 | 0.07 | 16 | 0.13 | 40 | 0.06 (0.05-0.09) |
| Rarely | 3 | 0.02 | 1 | 0.01 | 3 | 0.03 | 0 | 0.00 | 7 | 0.01 (0-0.02) |
| Never | 3 | 0.02 | 1 | 0.01 | 7 | 0.06 | 2 | 0.02 | 13 | 0.02 (0.01-0.04) |

Table S12. Opinions on FRDs: Community questionnaire response counts and proportions.

| | Control | | | | Neuter | | | | | |
|---|---|---|---|---|---|---|---|---|---|---|
| | Pre-sterilisation | | Post-sterilisation | | Pre-sterilisation | | Post-sterilisation | | | |
| | n | Proportion | n | Proportion | n | Proportion | n | Proportion | | |
| Variable | n_Control_1 | prop_Control_1 | n_Control_2 | prop_Control_2 | n_Neuter_1 | prop_Neuter_1 | n_Neuter_2 | prop_Neuter_2 | n | Proportion (95% CI) |
| **1.All dogs** | | | | | | | | | | |
| fewer | 7 | 0.04 | 2 | 0.01 | 9 | 0.08 | 17 | 0.15 | 35 | 0.06 (0.04-0.08) |
| same | 48 | 0.24 | 19 | 0.10 | 39 | 0.34 | 55 | 0.48 | 161 | 0.26 (0.22-0.29) |
| more | 140 | 0.71 | 176 | 0.89 | 57 | 0.50 | 36 | 0.31 | 409 | 0.65 (0.61-0.69) |
| none | 2 | 0.01 | 0 | 0.00 | 8 | 0.07 | 4 | 0.03 | 14 | 0.02 (0.01-0.04) |
| not_sure | 1 | 0.01 | 1 | 0.01 | 2 | 0.02 | 3 | 0.03 | 7 | 0.01 (0-0.02) |
| **2.Puppies** | | | | | | | | | | |
| fewer | 54 | 0.27 | 54 | 0.27 | 19 | 0.17 | 45 | 0.39 | 172 | 0.27 (0.24-0.31) |
| same | 34 | 0.17 | 44 | 0.22 | 15 | 0.13 | 19 | 0.17 | 112 | 0.18 (0.15-0.21) |
| more | 82 | 0.41 | 83 | 0.42 | 62 | 0.54 | 32 | 0.28 | 259 | 0.41 (0.38-0.45) |
| none | 26 | 0.13 | 2 | 0.01 | 17 | 0.15 | 12 | 0.10 | 57 | 0.09 (0.07-0.12) |
| not_sure | 2 | 0.01 | 15 | 0.08 | 2 | 0.02 | 7 | 0.06 | 26 | 0.04 (0.03-0.06) |
| **3.Barking dogs** | | | | | | | | | | |

| | Control | | | | Neuter | | | | | |
|---|---|---|---|---|---|---|---|---|---|---|
| | Pre-sterilisation | | Post-sterilisation | | Pre-sterilisation | | Post-sterilisation | | | |
| | n | Proportion | n | Proportion | n | Proportion | n | Proportion | | |
| Variable | n_Control_1 | prop_Control_1 | n_Control_2 | prop_Control_2 | n_Neuter_1 | prop_Neuter_1 | n_Neuter_2 | prop_Neuter_2 | n | Proportion (95% CI) |
| fewer | 36 | 0.18 | 10 | 0.05 | 12 | 0.10 | 34 | 0.30 | 92 | 0.15 (0.12-0.18) |
| same | 59 | 0.30 | 53 | 0.27 | 46 | 0.40 | 43 | 0.37 | 201 | 0.32 (0.28-0.36) |
| more | 103 | 0.52 | 134 | 0.68 | 51 | 0.44 | 35 | 0.30 | 323 | 0.52 (0.48-0.56) |
| not_sure | 0 | 0.00 | 1 | 0.01 | 2 | 0.02 | 3 | 0.03 | 6 | 0.01 (0-0.02) |
| none | 0 | 0.00 | 0 | 0.00 | 4 | 0.03 | 0 | 0.00 | 4 | 0.01 (0-0.02) |
| **4.Overweight dogs** | | | | | | | | | | |
| fewer | 43 | 0.22 | 60 | 0.30 | 26 | 0.23 | 7 | 0.06 | 136 | 0.22 (0.19-0.25) |
| same | 89 | 0.45 | 121 | 0.61 | 36 | 0.31 | 64 | 0.56 | 310 | 0.5 (0.46-0.54) |
| more | 2 | 0.01 | 3 | 0.02 | 5 | 0.04 | 11 | 0.10 | 21 | 0.03 (0.02-0.05) |
| none | 58 | 0.29 | 2 | 0.01 | 41 | 0.36 | 28 | 0.24 | 129 | 0.21 (0.18-0.24) |
| not_sure | 6 | 0.03 | 12 | 0.06 | 7 | 0.06 | 5 | 0.04 | 30 | 0.05 (0.03-0.07) |
| **5.Underweight dogs** | | | | | | | | | | |
| fewer | 72 | 0.36 | 88 | 0.44 | 42 | 0.37 | 29 | 0.25 | 231 | 0.37 (0.33-0.41) |

| | Control | | | | Neuter | | | | | |
|---|---|---|---|---|---|---|---|---|---|---|
| | Pre-sterilisation | | Post-sterilisation | | Pre-sterilisation | | Post-sterilisation | | | |
| | n | Proportion | n | Proportion | n | Proportion | n | Proportion | | |
| Variable | n_Control_1 | prop_Control_1 | n_Control_2 | prop_Control_2 | n_Neuter_1 | prop_Neuter_1 | n_Neuter_2 | prop_Neuter_2 | n | Proportion (95% CI) |
| same | 45 | 0.23 | 56 | 0.28 | 20 | 0.17 | 29 | 0.25 | 150 | 0.24 (0.21-0.28) |
| more | 4 | 0.02 | 36 | 0.18 | 11 | 0.10 | 14 | 0.12 | 65 | 0.1 (0.08-0.13) |
| none | 73 | 0.37 | 5 | 0.03 | 31 | 0.27 | 37 | 0.32 | 146 | 0.23 (0.2-0.27) |
| not_sure | 4 | 0.02 | 13 | 0.07 | 11 | 0.10 | 6 | 0.05 | 34 | 0.05 (0.04-0.08) |
| **6.Dogs with wounds** | | | | | | | | | | |
| fewer | 38 | 0.19 | 84 | 0.42 | 30 | 0.26 | 36 | 0.31 | 188 | 0.3 (0.26-0.34) |
| same | 6 | 0.03 | 30 | 0.15 | 23 | 0.20 | 25 | 0.22 | 84 | 0.13 (0.11-0.16) |
| more | 2 | 0.01 | 2 | 0.01 | 7 | 0.06 | 3 | 0.03 | 14 | 0.02 (0.01-0.04) |
| none | 149 | 0.75 | 58 | 0.29 | 45 | 0.39 | 46 | 0.40 | 298 | 0.48 (0.44-0.52) |
| not_sure | 3 | 0.02 | 24 | 0.12 | 10 | 0.09 | 5 | 0.04 | 42 | 0.07 (0.05-0.09) |

Table S13. Changes in FRD numbers: Community questionnaire response counts and proportions.

| term | OddsRatio | std.error | statistic | p.value | conf.low | conf.high | coef.type |
|---|---|---|---|---|---|---|---|
| **1.I am scared by stray dogs** | | | | | | | |
| disagree\|neutral | 2.0661 | 0.1744 | 4.1598 | 0.0000 | 1.4678 | 2.9084 | intercept |
| neutral\|agree | 12.0265 | 0.2318 | 10.7273 | 0.0000 | 7.6346 | 18.9447 | intercept |
| survey_number2 | 0.6300 | 0.2282 | -2.0246 | 0.0429 | 0.4028 | 0.9853 | location |
| SiteTypeNeuter | 1.6630 | 0.2670 | 1.9054 | 0.0567 | 0.9855 | 2.8063 | location |
| survey_number2:SiteTypeNeuter | 2.2865 | 0.3510 | 2.3562 | 0.0185 | 1.1492 | 4.5494 | location |
| **2.The stray dogs in this area are a problem** | | | | | | | |
| disagree\|neutral | 0.5179 | 0.1579 | -4.1653 | 0.0000 | 0.3800 | 0.7059 | intercept |
| neutral\|agree | 4.0070 | 0.1764 | 7.8706 | 0.0000 | 2.8360 | 5.6616 | intercept |
| survey_number2 | 1.5009 | 0.1926 | 2.1077 | 0.0351 | 1.0289 | 2.1894 | location |
| SiteTypeNeuter | 0.8516 | 0.2438 | -0.6590 | 0.5099 | 0.5280 | 1.3733 | location |
| aware_opsnot_sure | 0.5586 | 0.2281 | -2.5523 | 0.0107 | 0.3572 | 0.8736 | location |
| aware_opsyes | 0.9907 | 0.3179 | -0.0295 | 0.9765 | 0.5313 | 1.8472 | location |
| survey_number2:SiteTypeNeuter | 1.3679 | 0.3355 | 0.9337 | 0.3504 | 0.7087 | 2.6405 | location |
| **3.Stray dogs bark in this area** | | | | | | | |
| disagree\|neutral | 0.1525 | 0.2059 | -9.1330 | 0.0000 | 0.1018 | 0.2283 | intercept |
| neutral\|agree | 0.7612 | 0.1700 | -1.6055 | 0.1084 | 0.5455 | 1.0621 | intercept |
| survey_number2 | 2.2565 | 0.2287 | 3.5586 | 0.0004 | 1.4414 | 3.5326 | location |
| SiteTypeNeuter | 1.0168 | 0.2667 | 0.0625 | 0.9502 | 0.6029 | 1.7149 | location |
| survey_number2:SiteTypeNeuter | 0.2755 | 0.3521 | -3.6615 | 0.0003 | 0.1382 | 0.5493 | location |
| **4.Stray dogs in this area are aggressive to people** | | | | | | | |
| disagree\|neutral | 2.1586 | 0.1743 | 4.4154 | 0.0000 | 1.5341 | 3.0375 | intercept |
| neutral\|agree | 11.1333 | 0.2273 | 10.6038 | 0.0000 | 7.1313 | 17.3811 | intercept |
| survey_number2 | 1.8965 | 0.2147 | 2.9815 | 0.0029 | 1.2452 | 2.8885 | location |
| SiteTypeNeuter | 1.8379 | 0.2679 | 2.2721 | 0.0231 | 1.0872 | 3.1069 | location |

| term | OddsRatio | std.error | statistic | p.value | conf.low | conf.high | coef.type |
|---|---|---|---|---|---|---|---|
| survey_number2:SiteTypeNeuter | 0.6397 | 0.3388 | -1.3186 | 0.1873 | 0.3293 | 1.2427 | location |
| **5.Stray dogs in this area are aggressive to pet dogs** | | | | | | | |
| disagree\|neutral | 2.4663 | 0.2571 | 3.5105 | 0.0004 | 1.4899 | 4.0824 | intercept |
| neutral\|agree | 16.0743 | 0.3331 | 8.3383 | 0.0000 | 8.3680 | 30.8773 | intercept |
| survey_number2 | 0.8207 | 0.3578 | -0.5522 | 0.5808 | 0.4071 | 1.6548 | location |
| SiteTypeNeuter | 2.7990 | 0.3231 | 3.1852 | 0.0014 | 1.4857 | 5.2730 | location |
| survey_number2:SiteTypeNeuter | 1.0220 | 0.4488 | 0.0486 | 0.9612 | 0.4241 | 2.4630 | location |
| **6.I like stray dogs** | | | | | | | |
| disagree\|neutral | 2.4278 | 0.1762 | 5.0349 | 0.0000 | 1.7189 | 3.4290 | intercept |
| neutral\|agree | 12.2387 | 0.2181 | 11.4861 | 0.0000 | 7.9823 | 18.7648 | intercept |
| survey_number2 | 4.7991 | 0.2141 | 7.3273 | 0.0000 | 3.1547 | 7.3006 | location |
| SiteTypeNeuter | 2.8175 | 0.2504 | 4.1375 | 0.0000 | 1.7249 | 4.6022 | location |
| aware_opsnot_sure | 0.6597 | 0.2225 | -1.8694 | 0.0616 | 0.4266 | 1.0204 | location |
| aware_opsyes | 1.8872 | 0.3051 | 2.0814 | 0.0374 | 1.0378 | 3.4321 | location |
| dog_owneryes | 1.8875 | 0.1725 | 3.6830 | 0.0002 | 1.3461 | 2.6467 | location |
| survey_number2:SiteTypeNeuter | 0.2562 | 0.3434 | -3.9656 | 0.0001 | 0.1307 | 0.5022 | location |
| **7.I pet or play with stray dogs** | | | | | | | |
| disagree\|neutral | 5.1768 | 0.1989 | 8.2666 | 0.0000 | 3.5056 | 7.6447 | intercept |
| neutral\|agree | 28.3925 | 0.2442 | 13.7018 | 0.0000 | 17.5927 | 45.8221 | intercept |
| survey_number2 | 2.1080 | 0.2302 | 3.2389 | 0.0012 | 1.3424 | 3.3102 | location |
| SiteTypeNeuter | 2.8263 | 0.2572 | 4.0395 | 0.0001 | 1.7072 | 4.6789 | location |
| aware_opsnot_sure | 1.1501 | 0.2264 | 0.6177 | 0.5368 | 0.7380 | 1.7924 | location |
| aware_opsyes | 1.9058 | 0.2944 | 2.1903 | 0.0285 | 1.0702 | 3.3938 | location |
| dog_owneryes | 1.8837 | 0.1741 | 3.6376 | 0.0003 | 1.3392 | 2.6497 | location |
| survey_number2:SiteTypeNeuter | 0.4508 | 0.3595 | -2.2158 | 0.0267 | 0.2228 | 0.9121 | location |
| **8.Stray dogs should be sterilised** | | | | | | | |

| term | OddsRatio | std.error | statistic | p.value | conf.low | conf.high | coef.type |
|---|---|---|---|---|---|---|---|
| disagree\|neutral | 0.0052 | 0.7052 | -7.4556 | 0.0000 | 0.0013 | 0.0207 | intercept |
| neutral\|agree | 0.0205 | 0.5971 | -6.5106 | 0.0000 | 0.0064 | 0.0661 | intercept |
| survey_number2 | 0.6148 | 0.4512 | -1.0780 | 0.2810 | 0.2539 | 1.4887 | location |
| SiteTypeNeuter | 0.2118 | 0.5039 | -3.0803 | 0.0021 | 0.0789 | 0.5686 | location |
| survey_number2:SiteTypeNeuter | 1.9899 | 0.5996 | 1.1475 | 0.2512 | 0.6144 | 6.4448 | location |

b)

| Model | Control | Neuter |
|---|---|---|
| 1.I am scared of FRD | 398 | 238 |
| 2.FRD are a problem | 398 | 238 |
| 3.FRD bark | 398 | 238 |
| 8.FRD should be sterilised | 398 | 238 |
| 6.I like FRD | 398 | 238 |
| 7.I pet/play with FRD | 398 | 238 |
| 4.FRD aggressive to people | 398 | 238 |
| 5.FRD aggressive to pet dogs | 156 | 202 |

Table S14. Opinions on FRDs: Odds ratios and 95% confidence intervals from ordinal models for questions from community questionnaires and table showing sample sizes for each question. 'Agree' includes always and frequently, 'neutral' is sometimes, and 'disagree' is rarely and never.

| term | OddsRatio | std.error | statistic | p.value | conf.low | conf.high | coef.type |
|---|---|---|---|---|---|---|---|
| **1.All dogs** | | | | | | | |
| fewer\|same | 0.0310 | 0.3287 | -10.5711 | 0.0000 | 0.0163 | 0.0590 | intercept |
| same\|more | 0.4064 | 0.2030 | -4.4341 | 0.0000 | 0.2730 | 0.6051 | intercept |
| survey_number2 | 3.6182 | 0.2957 | 4.3491 | 0.0000 | 2.0268 | 6.4592 | location |
| SiteTypeNeuter | 0.3757 | 0.2973 | -3.2927 | 0.0010 | 0.2098 | 0.6728 | location |
| dog_owneryes | 1.5598 | 0.2302 | 1.9316 | 0.0534 | 0.9935 | 2.4490 | location |
| survey_number2:SiteTypeNeuter | 0.1239 | 0.4235 | -4.9308 | 0.0000 | 0.0540 | 0.2841 | location |
| **2.Puppies** | | | | | | | |
| fewer\|same | 0.4873 | 0.1889 | -3.8064 | 0.0001 | 0.3365 | 0.7056 | intercept |
| same\|more | 1.3321 | 0.1823 | 1.5730 | 0.1157 | 0.9319 | 1.9041 | intercept |
| survey_number2 | 1.0674 | 0.2218 | 0.2943 | 0.7685 | 0.6912 | 1.6486 | location |
| SiteTypeNeuter | 2.2086 | 0.3070 | 2.5806 | 0.0099 | 1.2099 | 4.0316 | location |
| dog_owneryes | 1.6847 | 0.2147 | 2.4287 | 0.0152 | 1.1059 | 2.5663 | location |
| survey_number2:SiteTypeNeuter | 0.2438 | 0.4001 | -3.5278 | 0.0004 | 0.1113 | 0.5340 | location |
| **3.Barking dogs** | | | | | | | |
| fewer\|same | 0.1423 | 0.2005 | -9.7233 | 0.0000 | 0.0960 | 0.2108 | intercept |
| same\|more | 1.0266 | 0.1594 | 0.1647 | 0.8692 | 0.7512 | 1.4031 | intercept |
| survey_number2 | 2.5477 | 0.2225 | 4.2038 | 0.0000 | 1.6474 | 3.9401 | location |
| SiteTypeNeuter | 1.0356 | 0.2574 | 0.1358 | 0.8920 | 0.6253 | 1.7150 | location |
| survey_number2:SiteTypeNeuter | 0.1499 | 0.3613 | -5.2522 | 0.0000 | 0.0738 | 0.3044 | location |
| **4.Overweight dogs** | | | | | | | |
| fewer\|same | 0.3989 | 0.2402 | -3.8255 | 0.0001 | 0.2491 | 0.6388 | intercept |
| same\|more | 110.7563 | 0.5623 | 8.3710 | 0.0000 | 36.7879 | 333.4507 | intercept |
| survey_number2 | 0.8171 | 0.2834 | -0.7126 | 0.4761 | 0.4689 | 1.4240 | location |
| SiteTypeNeuter | 0.6859 | 0.3907 | -0.9648 | 0.3346 | 0.3190 | 1.4752 | location |
| dog_owneryes | 0.5364 | 0.2813 | -2.2141 | 0.0268 | 0.3090 | 0.9310 | location |
| aware_opsnot_sure | 2.9985 | 0.5842 | 1.8797 | 0.0602 | 0.9542 | 9.4230 | location |
| aware_opsyes | 3.4683 | 0.9478 | 1.3122 | 0.1894 | 0.5412 | 22.2258 | location |
| survey_number2:SiteTypeNeuter | 9.8487 | 0.7898 | 2.8961 | 0.0038 | 2.0946 | 46.3079 | location |
| SiteTypeNeuter:aware_opsnot_sure | 0.7250 | 1.1576 | -0.2778 | 0.7811 | 0.0750 | 7.0094 | location |
| SiteTypeNeuter:aware_opsyes | 2.0490 | 1.3043 | 0.5500 | 0.5823 | 0.1590 | 26.4082 | location |
| **5.Underweight dogs** | | | | | | | |
| fewer\|same | 2.0213 | 0.2295 | 3.0657 | 0.0022 | 1.2889 | 3.1697 | intercept |
| same\|more | 12.8956 | 0.3119 | 8.1982 | 0.0000 | 6.9979 | 23.7638 | intercept |
| survey_number2 | 2.8589 | 0.2882 | 3.6454 | 0.0003 | 1.6252 | 5.0289 | location |
| SiteTypeNeuter | 1.0801 | 0.3881 | 0.1986 | 0.8426 | 0.5048 | 2.3112 | location |
| dog_owneryes | 1.5861 | 0.2625 | 1.7570 | 0.0789 | 0.9481 | 2.6533 | location |

| | | | | | | | |
|---|---|---|---|---|---|---|---|
| aware_opsnot_sure | 0.3753 | 0.4400 | -2.2270 | 0.0259 | 0.1584 | 0.8891 | location |
| aware_opsyes | 1.2450 | 0.5329 | 0.4112 | 0.6809 | 0.4381 | 3.5383 | location |
| survey_number2:SiteTypeNeuter | 1.0001 | 0.5353 | 0.0002 | 0.9999 | 0.3502 | 2.8556 | location |
| **6.Dogs with wounds** | | | | | | | |
| fewer\|same | 5.5295 | 0.4853 | 3.5240 | 0.0004 | 2.1361 | 14.3137 | intercept |
| same\|more | 49.4190 | 0.5851 | 6.6663 | 0.0000 | 15.6991 | 155.5657 | intercept |
| survey_number2 | 2.4631 | 0.6039 | 1.4926 | 0.1355 | 0.7541 | 8.0452 | location |
| SiteTypeNeuter | 5.1016 | 0.5807 | 2.8063 | 0.0050 | 1.6347 | 15.9213 | location |
| survey_number2:SiteTypeNeuter | 0.3537 | 0.7520 | -1.3819 | 0.1670 | 0.0810 | 1.5446 | location |

b)

| Model | Control | Intervention |
|---|---|---|
| 1.All dogs | 388 | 196 |
| 3.Barking dogs | 394 | 212 |
| 2.Puppies | 310 | 164 |
| 4.Overweight dogs | 254 | 98 |
| 5.Underweight dogs | 226 | 96 |
| 6.Dogs with wounds | 68 | 76 |

Table S15. Changes in FRDs: Odds ratios and 95% confidence intervals from ordinal models for responses to questions ‘Compared to last year, I have noticed…e.g. fewer puppies’ and table showing sample sizes for each question.

# 5. Appendix

## 5.1 Age assessment of dogs

Surveyors used these photos of known age dogs to estimate the age of observed dogs.

| Under 3 months | 3 months | 4-11 months | 12 months + |
|---|---|---|---|
| 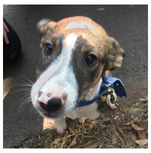 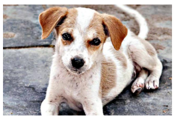 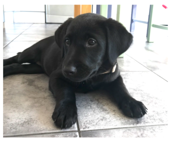 | 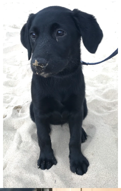 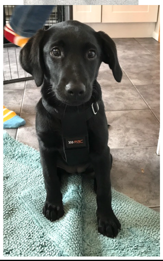 | 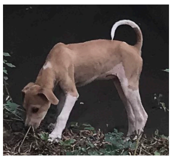 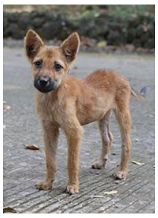 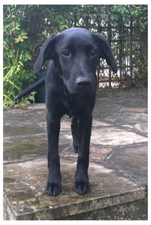 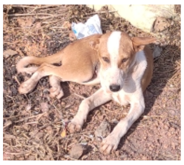 | 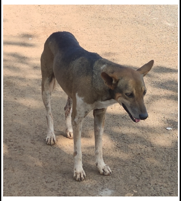 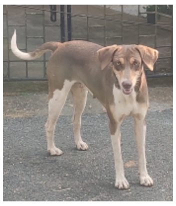 |

## 5.2 Skin condition

| Healthy skin | Mild (0-25% body coverage) | Moderate (26-50% body coverage) | Severe (>50% body coverage) |
|---|---|---|---|
| • Smooth coat all over | • Single patch of affected skin (e.g. hair loss, thickened skin) | • Affects more than one body area or is diffuse<br>• Less than 50% coverage | • More than 50% affected<br>• Severe hair loss/thickened skin |
| 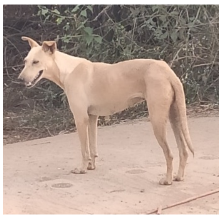 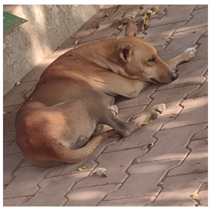 | 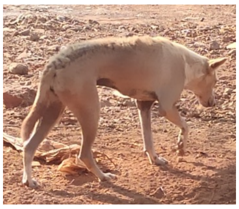 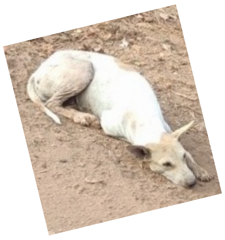 | 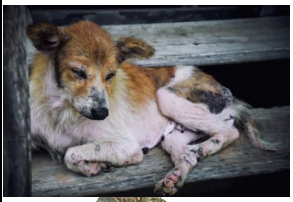 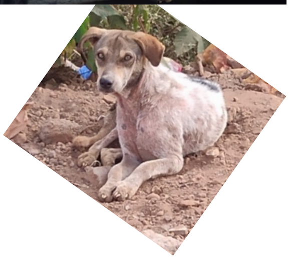 | 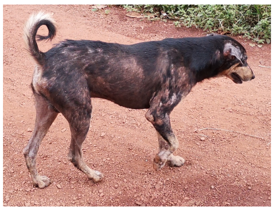 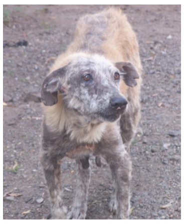 |

## 5.3 Body condition scoring

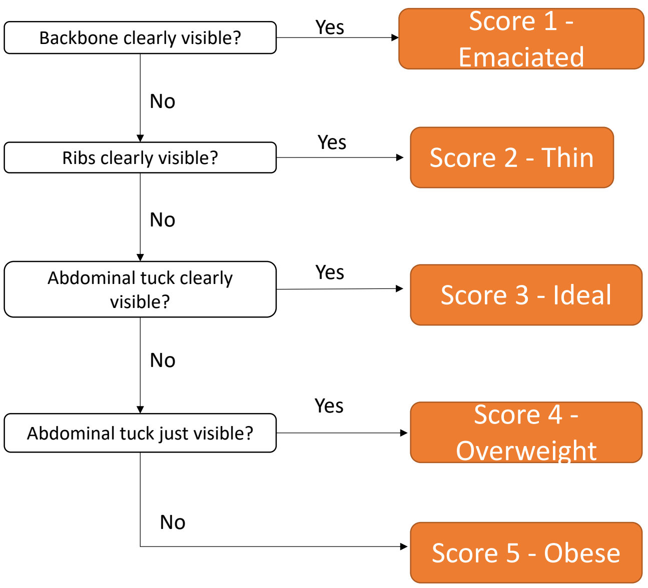


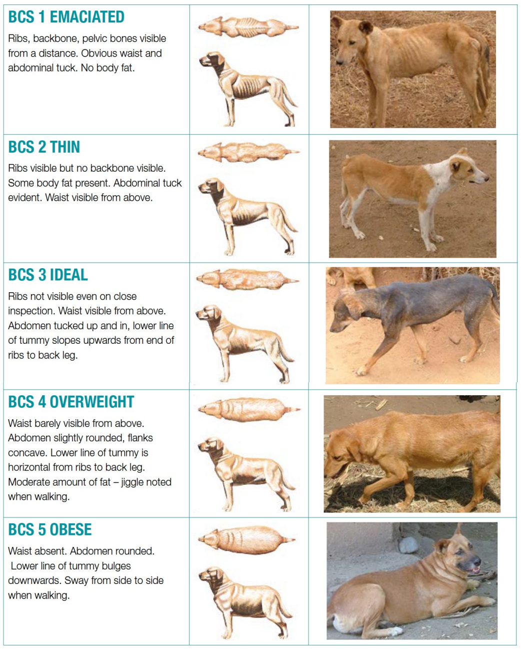


Resources developed from 'Are we making a difference?' report (International companion animal management coalition, 2015,right panel). ICAM acknowledgments: Descriptors for 5-point body condition score amended to be observation only without palpation from Food For Thought™ Technical Bulletin No. 77R; Innovative Research in Dog and Cat Nutrition™. Refinement of terms in descriptions and illustrations from Nestle Purina Body Condition System. Photos courtesy of Professor Darryn Knobel.

## 5.4 Community perceptions questionnaire

Any statements in *orange italics* will be read out in full to participants in their preferred language (Konkani, Hindi, English).

*'Good morning/afternoon/evening, my name is ….. and I work on the X project run by X. We would like to ask you some questions about your dogs and about stray dogs. The questions will take 5-10 minutes to complete.*

*Your answers, and those of other people we ask will help us better understand stray dogs in Goa. The DogPop project is a collaboration between X, X and X. Your participation is anonymous and voluntary, and you may skip any question you do not wish to answer. The data that you provide will be used for research purposes. By choosing to continue, you are consenting to participate, are you happy to continue?'*

If no…. *Thank you very much for your time. Have a great day!*

**Dog ownership**

1. Do you own a dog?

a. Yes
b. No

*-----Dog link form – complete one for each dog in household -----*

2. What sex is your dog?
3. If female, is she lactating?
4. Has she had puppies in the last 12 months?
5. How many litters of puppies has she ever had?
6. If she has had puppies, what happened to them?
7. How old is your dog?
    a. Puppy (0-3m)
    b. Juvenile (4-11m)
    c. Adult (12m+)
    d. Unknown
8. What breed is your dog?
    a. Indy dog/mixed breed
    b. Pure-bred/Pedigree
9. Is your dog neutered?
    a. Yes
    b. No
    c. I don't know
10. Does your dog wear a collar?
11. What is your dog's body condition? (If puppy/juvenile – can you see the dog's ribs?)
    a. Emaciated
    b. Thin
    c. Ideal
    d. Overweight
    e. Obese
12. Does your dog have a skin condition?
13. Does your dog have any current health problems?
14. If your dog has wounds, where are they located?
15. What kind of a place did you get your dog from?
    a. Adopted from street
    b. Adopted from shelter
    c. Home-bred
    d. Purchased from breeder
    e. Gift
    f. Other (please state)
16. Where did your dog come from?
    a. This household (one of my other dog's puppies)
    b. Local area (within 1km of the house)
    c. Over 1km away but within my panchayat/municipality
    d. North Goa (outside my local area)
    e. South Goa (outside my local area)
    f. Outside Goa (another state or country)
17. Does your dog ever leave the property?
    a. Yes
    b. Yes but only on a leash/lead/chain
    c. No
18. When is your dog allowed to leave the property?
    a. Day
    b. Night
    c. Day and night
    d. Morning
    e. Evening
    f. Other
19. Approximately how long is your dog allowed to leave the property per day?
    a. Less than 1 hr
    b. 1-5 hours
    c. Over 5 hours
    d. Unknown

---

*'For the purpose of this questionnaire, by stray dog, we mean a dog that is unconfined for significant time periods and allowed to interact freely with other unconfined dogs.*

*Sterilisation is an operation which prevents dogs (females and males) from breeding and/or having puppies. This is different from a vaccination, which protects the dog from a disease.'*

*The next section is about stray dogs in the area you live. I will read out a series of statements to which you can respond: strongly agree, agree, neither agree nor disagree, disagree, strongly disagree. There are no right or wrong answers, please answer truthfully.'*

1. I like stray dogs
2. Stray dogs should be sterilised (operation to stop breeding/having puppies)
3. I am scared by stray dogs
4. Stray dogs bark in this area
5. I like to pet/play stray dogs
6. The stray dogs in this area are a problem
7. Stray dogs in this area are aggressive to people
8. Stray dogs in this area are aggressive to pet dogs
9. Stray dogs in this area are aggressive with each other

1. Are you aware of any large-scale dog sterilisation activities in this area (this does NOT include vaccination OR surveys)?
    a. Yes
    b. No
    c. I'm not sure
2. If yes, how do you feel about this?
    a. Strongly disagree
    b. Disagree
    c. Neither agree nor disagree
    d. Agree
    **e.** Strongly agree
3. If yes, why do you think this is happening?
    a. Government programme
    b. Charity programme
    c. Local community action
    d. Other (please state)
    e. Don't know
4. If charity programme, which one?
    a. X
    b. X
    c. Other (please state)
    d. Don't know

**Dog health**

5. Compared to a year ago, I have noticed
    a. More stray dogs
    b. Around the same number of stray dogs
    c. Fewer stray dogs
    d. No stray dogs
    e. I don't know
6. Compared to a year ago, I have noticed
    a. More puppies
    b. Around the same number of puppies
    c. Fewer puppies
    d. No puppies
    e. I don't know
7. Compared to a year ago, I have noticed
    a. More wounds on stray dogs
    b. Around the same number of wounds on stray dogs
    c. Fewer wounds on stray dogs
    d. No wounds on stray dogs
    e. I don't know
8. Compared to a year ago, I have noticed
    a. More fat stray dogs
    b. The same number of fat stray dogs
    c. Fewer fat stray dogs
    d. No fat stray dogs
    e. I don't know
9. Compared to a year ago, I have noticed
    a. More thin stray dogs
    b. The same number of thin stray dogs
    c. Fewer thin stray dogs
    d. No thin stray dogs
    e. I don't know
10. Compared to a year ago, I have noticed
    a. More dogs barking
    b. The same amount of barking
    c. Less barking
    d. No barking

e. I don't know

*Do not read out the answers to the following questions, allow the person to answer.*

11. Where do new stray dogs or puppies come from in your opinion? (multiple entries can be selected)
    a. Existing stray dog population giving birth
    b. Owned dogs giving birth - unwanted puppies dumped by owners
    c. New dogs coming in from other areas
    d. People bringing in new dogs
    e. Other (please state)
12. Where do you think stray dogs get their food? (multiple entries can be selected)
    a. Predation of livestock or wildlife
    b. Garbage dumps
    c. Restaurants/meat shop/market
    d. Fed by residents or dog feeders
    e. Fed by tourists
    f. Other (please state)

**Contact with stray dogs**

13. Do you ever feed stray dogs?
    a. Yes
    b. No
14. If yes, how often do you feed stray dogs? (do not read out answers)
    a. Daily
    b. A few times a week
    c. A few times a month
    d. Less than a few times a month
    e. I don't know

**Demographic**

*'We will now ask some questions about you – any information you give is entirely anonymous and will not be disclosed to anyone else.'*

1. What age category are you in?
   a. 18-25
   b. 26-35
   c. 36-45
   d. 46-55
   e. 56-70
   f. Over 70
   g. Prefer not to say
2. What is your gender?
   a. Male
   b. Female
   c. Other?
   d. Prefer not to say
3. What is your religion?
   a. Hinduism
   b. Christianity
   c. Islam
   d. Buddhism
   e. Sikhism
   f. Jainism
   g. None
   h. Other
   i. Prefer not to say
4. What is the highest level of education you have achieved?
   a. No education
   b. Some primary
   c. Complete primary
   d. Some secondary
   e. Complete secondary
   f. College education
   g. Higher
   h. Prefer not to say

5. What is your average monthly household income (in Indian Rupees)?
    a. Under 12,500/-
    b. 12,500/- to 28,000/-
    c. 29,000/- to 60,000/-
    d. 61,000/- to 140,000/-
    e. Over 141,000
    f. Unknown
    g. Prefer not to say

*'Thank you so much for your participation and time. As part of the study, it would help us greatly if we can ask you some similar questions next year…*

6. Are you happy to be contacted again?
    a. Yes
    b. No

## 6. SI References


[1] A. D. Gibson *et al.*, "Vaccinate-assess-move method of mass canine rabies vaccination utilising mobile technology data collection in Ranchi, India," *BMC Infect Dis*, vol. 15, p. 589, 2015, doi: 10.1186/s12879-015-1320-2.

[2] H. R. Fielding *et al.*, "Capturing free-roaming dogs for sterilisation: A multi-site study in Goa , India," *Prev Vet Med*, vol. 218, no. August, p. 105996, 2023, doi: 10.1016/j.prevetmed.2023.105996.

[3] International companion animal management coalition, "Are We Making a Difference?," 2015. doi: 10.1089/jpm.2016.0425.

[4] E. Hiby and L. Hiby, "Direct observation of dog density and composition during street counts as a resource efficient method of measuring variation in roaming dog populations over time and between locations," *Animals*, vol. 7, no. 8, 2017, doi: 10.3390/ani7080057.

[5] QGIS Development team, *QGIS*. Open Source Geospatial Foundation, 2019.

[6] R Core Team, *R: A Language and Environment for Statistical Computing.* Vienna, Austria: R Foundation for Statistical Computing, 2021. [Online]. Available: https://www.r-project.org/

[7] D. Bates, M. Mächler, B. Bolker, and S. Walker, "Fitting Linear Mixed-Effects Models Using lme4," *J Stat Softw*, vol. 67, no. 1, pp. 1–48, 2015, doi: 10.18637/jss.v067.i01.

[8] D. Lüdecke, "sjPlot: Data Visualization for Statistics in Social Science," 2023, R package version 2.8.15.

[9] R. M. Fewster, S. T. Buckland, G. M. Siriwardena, S. R. Baillie, and J. D. Wilson, "Analysis of population trends for farmland birds using generalized additive models," *Ecology*, vol. 81, no. 7, pp. 1970–1984, 2000, doi: 10.1890/0012-9658(2000)081[1970:AOPTFF]2.0.CO;2.

[10] A. F. Zuur, E. N. Ieno, N. J. Walker, A. A. Saveliev, and G. M. Smith, *Mixed Effects Models and Extensions in Ecology with R*. New York: Springer, 2009.

[11] S. N. Wood, "Fast stable restricted maximum likelihood and marginal likelihood estimation of semiparametric generalized linear models," *Journal of the Royal Statistical Society (B)*, vol. 73, no. 1, pp. 3–36, 2011, doi: 10.1111/j.1467-9868.2010.00749.x.

[12] D. G. Chapman, "Some properties of the hypergeometric distribution with applications to zoological sample censuses," *University of California Publications in Statistics*, vol. 1, no. 7, pp. 60–131, 1951.

[13] R. H. B. Christensen, *ordinal---Regression Models for Ordinal Data*, R package. 2015. [Online]. Available: http://www.cran.r-project.org/package=ordinal/
